# Which Factors Determine Spatial Segregation in the South American Opossums (*Didelphis aurita* and *D*. *albiventris*)? An Ecological Niche Modelling and Geometric Morphometrics Approach

**DOI:** 10.1371/journal.pone.0157723

**Published:** 2016-06-23

**Authors:** Nilton Carlos Cáceres, Marcelo de Moraes Weber, Geruza Leal Melo, Carlo Meloro, Jonas Sponchiado, Renan dos Santos Carvalho, Jamile de Moura Bubadué

**Affiliations:** 1 Laboratório de Ecologia e Biogeografia, Departamento de Ecologia e Evolução, Universidade Federal de Santa Maria, Santa Maria, Brazil; 2 Laboratório de Ecologia Teórica e Síntese, Instituto de Ciências Biológicas, Universidade Federal de Goiás, Goiânia, Brazil; 3 Programa de Pós-Graduação em Ecologia e Conservação, CCBS, Universidade Federal do Mato Grosso do Sul, Campo Grande, Brazil; 4 Research Centre in Evolutionary Anthropology and Palaeoecology, School of Natural Sciences and Psychology, Liverpool John Moores University, Liverpool, England; 5 Programa de Pós-Graduação em Biodiversidade Animal, CCNE, Universidade Federal de Santa Maria, Santa Maria, Brazil; University of Naples, ITALY

## Abstract

*Didelphis albiventris* and *D*. *aurita* are Neotropical marsupials that share a unique evolutionary history and both are largely distributed throughout South America, being primarily allopatric throughout their ranges. In the Araucaria moist forest of Southern Brazil these species are sympatric and they might potentially compete having similar ecology. For this reason, they are ideal biological models to address questions about ecological character displacement and how closely related species might share their geographic space. Little is known about how two morphologically similar species of marsupials may affect each other through competition, if by competitive exclusion and competitive release. We combined ecological niche modeling and geometric morphometrics to explore the possible effects of competition on their distributional ranges and skull morphology. Ecological niche modeling was used to predict their potential distribution and this method enabled us to identify a case of biotic exclusion where the habit generalist *D*. *albiventris* is excluded by the presence of the specialist *D*. *aurita*. The morphometric analyses show that a degree of shape discrimination occurs between the species, strengthened by allometric differences, which possibly allowed them to occupy marginally different feeding niches supplemented by behavioral shift in contact areas. Overlap in skull morphology is shown between sympatric and allopatric specimens and a significant, but weak, shift in shape occurs only in *D*. *aurita* in sympatric areas. This could be a residual evidence of a higher past competition between both species, when contact zones were possibly larger than today. Therefore, the specialist *D*. *aurita* acts a biotic barrier to *D*. *albiventris* when niche diversity is not available for coexistence. On the other hand, when there is niche diversification (e.g. habitat mosaic), both species are capable to coexist with a minimal competitive effect on the morphology of *D*. *aurita*.

## Introduction

Congeneric similar species generally occur allopatrically (= in separate geographic areas), or at least parapatrically (= in adjacent geographic space). However, the range of these species in the past could have been different from the current one if speciation events took place, originating ecologically similar competing species. Thus, competitive exclusion or competitive release could have taken place between such species given sufficient time [1,2,3]. Morphologically similar species must meet some requirements to permit testing geographic predictions on competitive exclusion and competitive release. Focal species should not co-occur broadly in sympatry, but rather show narrow contact zones, providing support for competition as main factor influencing geographic manifestations in their realized distributions. Also, the environmental tolerances of the focal species should differ significantly but show partial overlap, providing some regions of potential sympatry where competitive exclusion could occur [4].

Clear distributional predictions of competitive release exist for species pairs with similar ecological requirements that do not exist in broad zones of sympatry [1,2,3,5]. If one species excludes another from areas of potential overlap, the inferior competitor would be predicted to inhabit suboptimal environmental conditions in biogeographic regions where the other species is not present–in comparison with the conditions it inhabits in regions where both species exist [4]. The method of ecological niche modeling (ENM) has proved to be useful for predicting speciation mechanisms of ecologically similar related species [3,4,5].

When two or more related species overlap in their distributions, changes in animal shape due to character displacement are quite common [6]. This is true, for example, in carnivores, where sympatric species differ usually in tooth morphology, which is thought to be linked to an improved feeding efficiency [7,8]. The leaf-specialist southern howler monkey (*Alouatta guariba*) represents another similar case of morphological change in response to a competitive pressure of the larger (ecologically similar) *Brachyteles* [9]. Irrespective of diet, character displacement is clearly found in many mammalian groups including bats [10], rodents [11], and insectivores [12].

In South America, large marsupial species of the genus *Didelphis*, the White-eared Opossum (*Didelphis albiventris*) and the Brazilian Common Opossum (*D*. *aurita*) diverge by 5.7% in molecular terms [13]. As expected due to morphological and molecular proximity, these species are primarily allopatric in their geographical ranges [14,15], and even locally [16]. Based on their current distributions, *D*. *albiventris* can be classified as a more versatile species, adapted to areas of more seasonal climate, such as the Cerrado and Pampas biomes, but also occurring in forest areas with relatively large body weight (500–2700 g) [14,17,18,19]. On the other hand, *D*. *aurita* is a smaller taxon (body weight: 670-1800g) and a typically forest dweller species living in the Atlantic forest [14,19,20,21]. Still on a broad geographical scale (i.e., South America or the Araucaria moist forest biome), these two species show several spatial overlapping areas [13,22].

The Araucaria moist forest is typical of uplands in southern Brazil, showing grasslands as predominant vegetation and scattered forest patches. It is colder in relation to the rest of the Atlantic forest, creating an ecotonal region between cold and hot weather which is altitude dependent. In this region, when in sympatry, *D*. *aurita* and *D*. *albiventris* can share the same physical space, with the former occupying forested areas and the latter occupying open and forest edge areas [16]. Furthermore, *D*. *albiventris* is an opossum species more adapted to environmental disturbance than *D*. *aurita* [23,24].

One study in a region of sympatry involving these two species in the Araucaria moist forest shows that the territorial behavior of *D*. *aurita* females directly displaces *D*. *albiventris* females from remnants interior and stream sides, which are the richest part of the patches [16]. Except for this study that shows potential conflict between both species, nothing is known about their potential distributions. Since these species are similar both morphologically and genetically [13], we have no evidence of how each species would be distributed in South America regardless the presence of a congeneric competitor as a barrier.

The aim of this study is to evaluate how the two opossum species *D*. *albiventris* and *D*. *aurita* occupy the geographical space, based on their niche and morphological features by combining two approaches: ecological niche modeling and geometric morphometrics. Specifically, the goals of the ecological niche modeling are: to characterize the niche spatial similarity between the two species at three geographic scales [continental (South America), biome (Cerrado and Atlantic forest), and ecoregion (Araucaria moist forest)]; and to estimate how a species may be interfering in the distribution of the other, limiting its occurrence in environmentally suitable areas for occupation. A previous study using ENM has shown that one species of the mouse opossum (*Marmosa xerophila*) limit the distribution of its close relative (*M*. *robinsoni*) in northwestern Venezuela (an area climatically suitable for both of them) possibly due to direct competition [3]. This might similarly apply to the members of the genus *Didelphis*. At a broad geographical scale, *D*. *albiventris* should be potentially distributed extensively within the range of *D*. *aurita*, but mainly in marginal, dry-forest areas, given its ecological generalism, whereas *D*. *aurita* should maintain its typical distribution along the Atlantic forest. For the Araucaria moist forest, where there is co-occurrence of forests and grasslands [25], our hypothesis is that *D*. *aurita* might potentially occur in forested areas in the east and the west, where continuous forest patches predominate. On the other hand, *D*. *albiventris* should occur mainly in central areas and in the south of Araucaria, where open habitats connected to the Pampas prevail [22].

Using geometric morphometrics, we test if the skull size and shape of both species changes when in sympatry. We expect that morphological changes will occur if competition is strong when species overlap extensively in range [26]. Cranial muscles involved in the bite force should be theoretically improved in the species that is ecologically more specialized (*D*. *aurita*) to counterbalance the more environmental flexible species, *D*. *albiventris*. Also when in sympatry the two species should exhibit greater cranial differences as compared to allopatric specimens [27].

## Materials and Methods

### Ecological Niche Modeling

Occurrence data for *Didelphis aurita* and *Didelphis albiventris* were gathered through 1) literature review (see the reference list in S1 Table), 2) online databases Global Biodiversity Information Facility (GBIF) (http://www.gbif.org/) and Mammal Networked Information System (MaNIS) (http://manisnet.org/), 3) specimens deposited in museums in Brazil and 4) personal records of the species in the field made by the authors (NCC, JS, and GLM)–approved by the Instituto Chico Mendes de Conservação da Biodiversidade (ICMBio) (protocols 30808–2, 19661–1, 2205–1, 1131–1, 1401–1, 2203–1, 2383–8, 24558–1 and 11504–1). All records were georeferenced and for those that did not contain locality information we used central coordinates of the municipality of collection. We also determined the collection year for each specimen.

**Fig 1:**
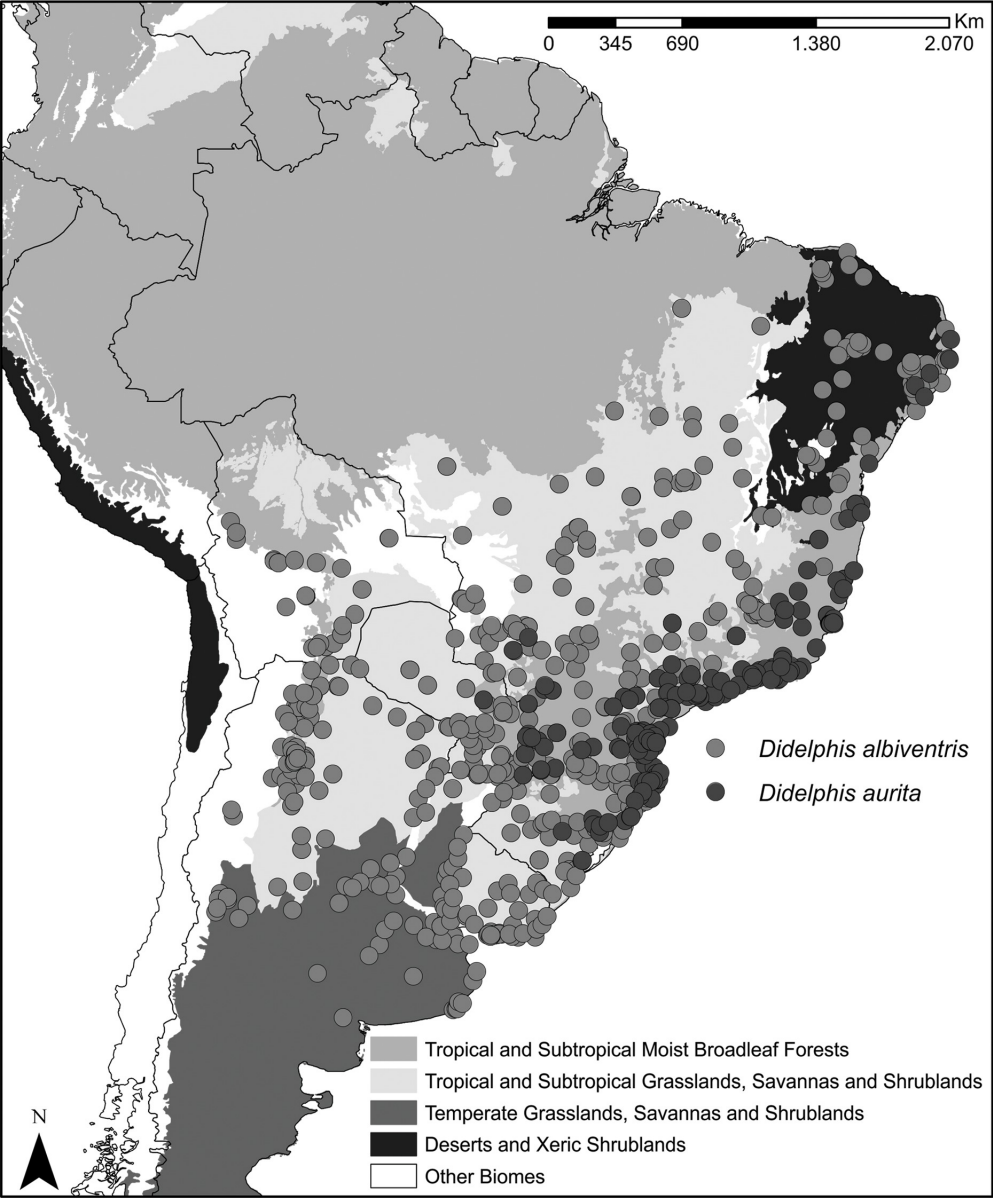
Map showing the localities where *Didelphis aurita* and *Didelphis albiventris* have been recorded in South America. **The publicly available map layer was obtained from http://www.arcgis.com/features/features.html and the image prepared with the ArcMap 10 (ESRI Inc.).** Sampling localities of different species are shown by different symbols.

Our sampling included 577 occurrence records for *Didelphis albiventris* and 197 for *D*. *aurita*, covering the whole species range (Fig 1).

Before running ENMs, we removed duplicated records for each species. Species occurrence data are generally spatially biased and therefore environmental bias is likely to occur as well. Such biases can lead to inaccurate niche models with an over-representation of environmental conditions associated to regions with higher sampling effort [28]. Furthermore, occurrence points close to each other may not be considered spatially independent. In order to account for such non-independent and spatially biased data, we used the spatial thinning that results in species occurrence data that yield better performing ENMs [29,30]. We removed occurrence records so that two geographical points are not closer than 12 km in a linear distance resulting in a minimum nearest neighbor distance (NND) greater than or equal to 12 km. We chose this threshold based on the widest home range linear extent recorded for the species set (*D*. *aurita*'s home range is 11 km and *D*. *albiventris'* home range is 6 km, [31,32]). Thus 12 km will assure independent occurrence data for both species. To retain the greatest amount of niche information, records should be thinned such that the largest possible number of occurrence points is retained. Since there are several occurrence data combinations that yield the largest possible number of occurrences, we ran the spatial thinning using 100 replicates as the maximum number of files with occurrence that mitigates the effects of biased sampling. Spatial thinning yielded 10 replicates for *D*. *aurita* and four for *D*. *albiventris*. We used each replicate to build a niche model. We ran spatial thinning using the *spThin* package [29] in the R environment [33].

Climatic data were obtained from WorldClim [34] with a spatial resolution of 30” (~ 1 km) to model species niche under current climatic conditions. We extracted values of 19 bioclimatic variables at the localities where species have been recorded. After that, we portrayed all variables in a correlation matrix, selecting only those that presented correlation values lower than 0.7 [35]. Same set of variables was used to model both species niche, as follows: BIO2 (Mean Diurnal Range), BIO4 (Temperature Seasonality), BIO5 (Maximum Temperature of Warmest Month), BIO8 (Mean Temperature of Wettest Quarter), BIO9 (Mean Temperature of Driest Quarter), BIO12 (Annual Precipitation), BIO13 (Precipitation of Wettest Month), BIO15 (Precipitation Seasonality), BIO18 (Precipitation of Warmest Quarter), BIO19 (Precipitation of Coldest Quarter).

For each *Didelphis* species we used three different algorithms to model their climatic niches: Euclidean Distance [36,37,38], Maximum Entropy (Maxent, [39]) and SVM (Support Vector Machine, [35,40,41]). Maxent was run in Maxent 3.3.3.k [39], while SVM and Euclidean Distance algorithms were run in Open Modeller [40]. Occurrence data were divided into two subsets, train and test, which should be preferably independent [42]. In order to account for independent subsets of occurrence data, we assigned those occurrence points recorded from 2000–2010 as test data and the remaining occurrence points as train data. Training data consisted most of presence records (429 to *D*. *albiventris* and 139 to *D*. *aurita*) and they were used to adjust the model to the data. Testing data consisted of a smaller sample of presence data (75 to *D*. *albiventris* and 27 to *D*. *aurita*). We used both the area under the ROC curve (AUC) and the Boyce Index (BI) to measure the accuracy of the models because relying only on the AUC values has been criticized [43,44]. AUC values near 0.5 indicate the model has no predictive capacity, while AUC values near 1 indicate the model had good to excellent performance and we can discriminate properly between climatically suitable and unsuitable areas [45]. BI varies from -1 to 1, where positive values indicate the model is consistent with the presence data and has good predictive capacity and negative values indicate an incorrect model, predicting poor suitable areas where species is more frequent. Values close to zero indicate the model is no better than random [46]. Models with AUC ≥ 0.75 and BI ≥ 0.25 were considered as having good performance [47]. Models with AUC<0.75 and BI<0.25 were excluded from the analysis. Since we have 10 replicates of occurrence data after thinning for *D*. *aurita* and four for *D*. *albiventris*, we yielded 30 niche models for *D*. *aurita* (3 algorithms x 10 replicates) and 12 for *D*. *albiventris* (3 algorithms x 4 replicates).

After building the niche models for each species, we created a consensus model (ensemble). Ensemble consists in combining different results from different algorithms and/or climatic scenarios to generate a consensus model among them [48,49] in order to prevent possible idiosyncrasies inherent to the algorithms used. To construct a consensus model, we averaged all models for each species, hence obtaining a single ecological niche model for each species.

### Ecological Niche Similarity Analysis

The consensus models for *D*. *aurita* and *D*. *albiventris* were overlapped to calculate the level of ecological niche similarity between species. The niche similarity calculation was made through the difference between the environmental suitability values in each pixel within the model. Thus, we used the equation $S=1-\sqrt{{\left(xi-yi\right)}^{2}}$, where S is the niche similarity level in pixel *i*, *x*_*i*_ is the environmental suitability value for the species *x* in the pixel *i* and *y*_*i*_ is the environmental suitability value for the species *y* in pixel *i*. Values near 1 indicate high level of niche similarity while values near zero indicate low level of niche similarity. Therefore, for each pixel it is possible to visualize the level of ecological niche similarity through geographic space. The niche similarity analyses were performed under three geographic scales: continental (South America); biome (Cerrado and Atlantic forest); and ecoregion (Araucaria moist forest). To estimate the level of niche similarity between *Didelphis* species at those geographic scales described above, we ran the Schoener’s *D* similarity statistic [50] representing the similarity of the two distributions. *D* statistic ranges from zero (no similarity) to one (maximum similarity). Niche similarity test addresses whether the environmental suitability occupied in one species range is more similar (or different) to the one occupied in the other species range than expected by chance [51]. Rejection of the null hypothesis indicates that one species occupies environments in both ranges that are more similar to each other than expected by chance [50,51]. We followed [52] approach to calculate the *D* similarity index. We calculated *D* similarity statistic and its significance (*P*<0.05) as well as the Boyce Index using the package *ecospat* [53] in the R environment.

### Morphological Analyses

**Fig 2:**
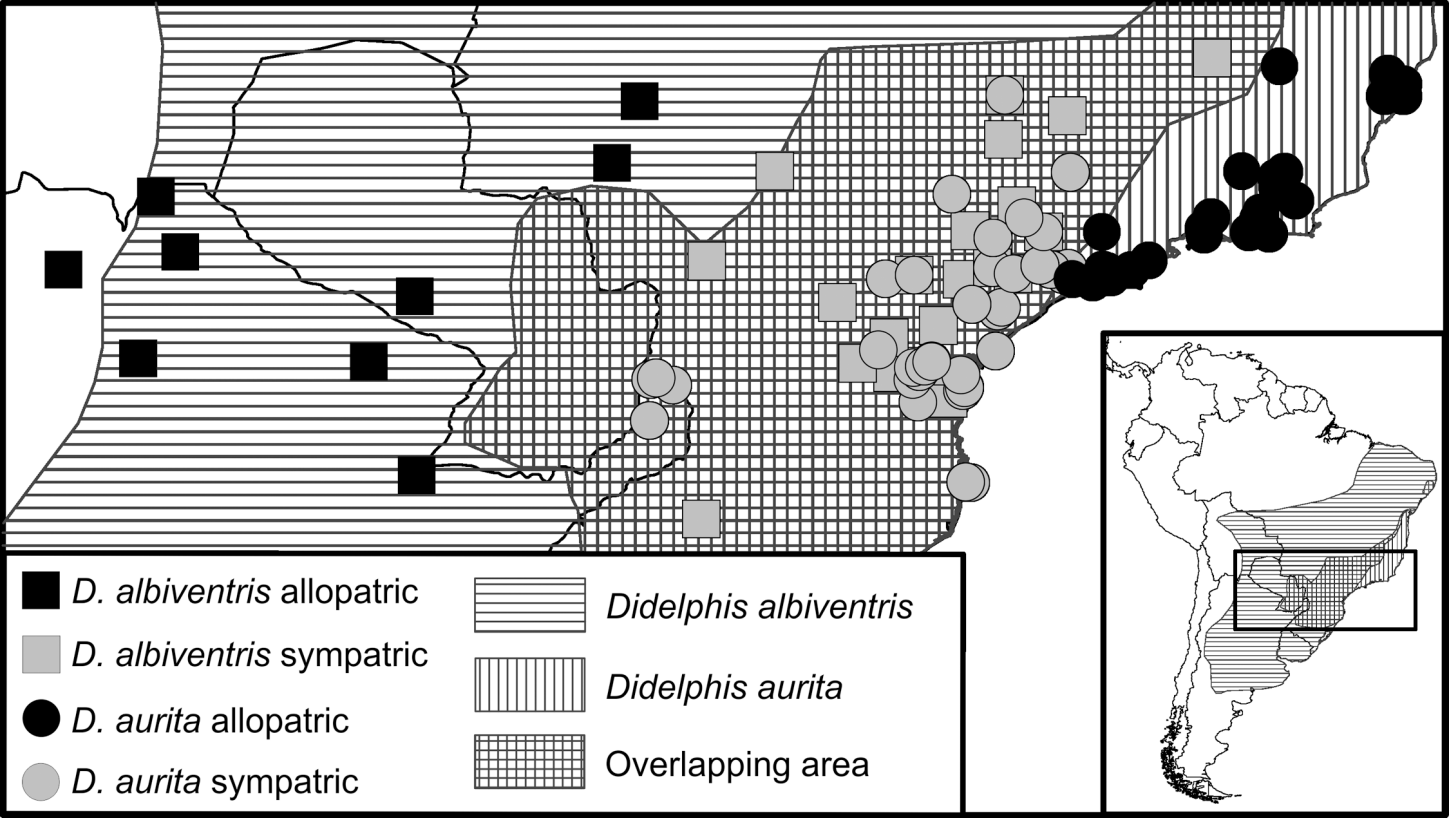
Map showing the geographic distribution of our samples used for geometric morphometrics analyses. **The publicly available map layer was obtained from http://www.arcgis.com/features/features.html and the image prepared with the ArcMap 10 (ESRI Inc.).** The horizontal and vertical lines correspond to the IUCN map distribution for *Didelphis albiventris* and *D*. *aurita* used to categorize the specimens as sympatric/allopatric. Sampling localities of different species and different local categories (allopatric and sympatric) are shown by different symbols.

**Fig 3:**
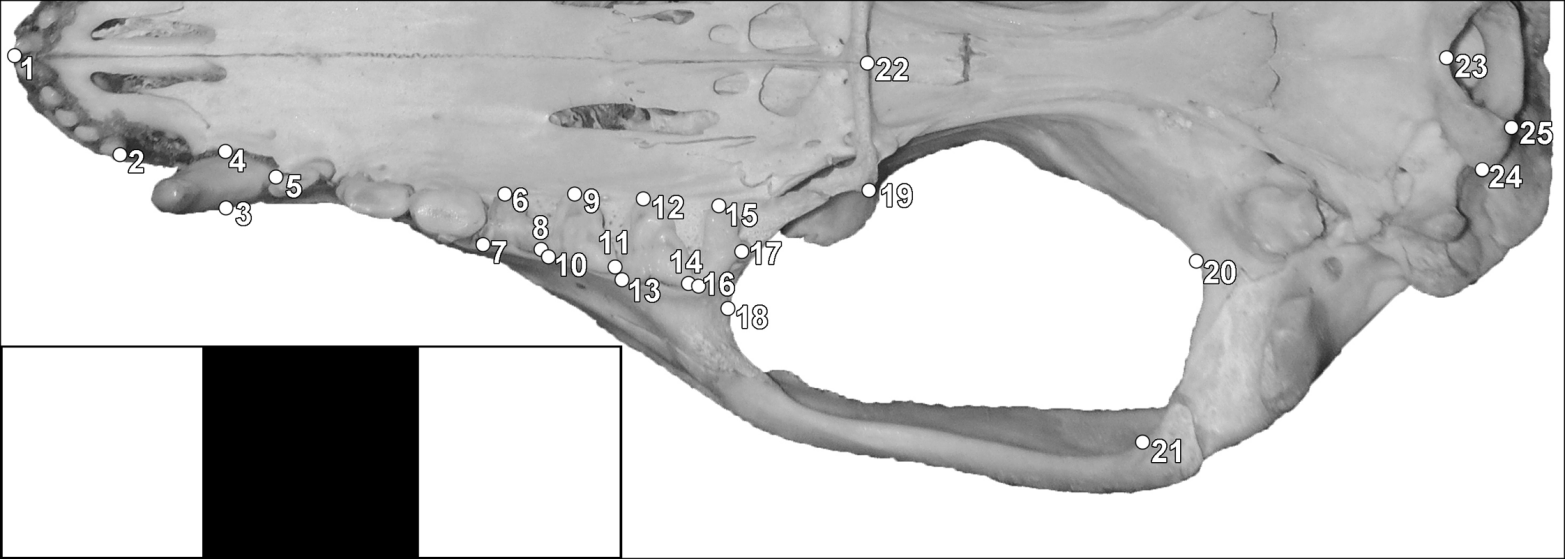
Disposition of 25 landmarks on a skull of *Didelphis albiventris* specimen. 1 = midpoint of central incisors; 2 = posterior-most point of lateral incisor alveolus; 3–5 = canine area; 5–7 = pre-molar series length; 6–8 = first molar area; 9–11 = second molar area; 12–14 = third molar area; 15–17 = fourth molar area; 18–21 = temporal muscle insertion area; 22 = most posterior tip of the palatine; 23–25 = occipital condyle area.

We sampled 159 wild-caught adult specimens of *Didelphis aurita* (67 females, 92 males) and 75 *Didelphis albiventris* (38 females, 37 males) in similar latitude range around south-east of Brazil to reduce the geographical effect (Fig 2). The database consisted of skull digital photographs in ventral view and the field data of each specimen (species, sex, sample locality, and museum). We used the software tpsDig 2 [54] to build a database of digital pictures and recorded two-dimensional spatial coordinates of twenty-five homologous landmarks. The landmark configuration (Fig 3) was chosen to accurately describe skull features in ventral view of *Didelphis*, including the temporal muscle insertion area (zygomatic arch), the rostrum area (palate), the auditory bulla area, and the relative position of upper teeth.

We categorized the specimens according to sex and geographic location here reduced into two categories based on local coexistence between *D*. *aurita* and *D*. *albiventris*: allopatric or sympatric, factor named ‘geography’ (S2 Table). Sympatric ranges between opossum species were defined based on the overlap of species range maps provided by the International Union for Conservation of Nature (IUCN; http://www.iucnredlist.org/).

Generalized Procrustes Analysis (GPA) was employed to translate, rotate and scale the original landmark coordinates and generate shape variables (the procrustes coordinates). Centroid size (= the square root of the sum of the squared distances between each landmark and configuration centroid) was extrapolated from the original landmark coordinates as a proxy for skull size to test the impact of allometric effect on skull shape changes [55].

We used TpsRelw [54] to perform a principal component analysis (named Relative Warp Analysis, RWA) of the shape coordinates and visualize graphically the shape variance between species and sex with the support of thin plate spline.

In the R environment, version 2.8.1 [33], using the R packages geomorph [56], we performed a two-way Procrustes ANOVA via 9,999 permutations testing for differences between species and sexes (factors) in *Didelphis* skull shape (response variable, 50 procrustes coordinates). We also performed a one-way Procrustes ANOVA model via 9,999 permutations testing the geography factor using the full sample, and then separating by species and sex. Finally, we conduced ANCOVA models via 9,999 permutations adding skull size (= natural log transformed CS) as a covariate to test for species, sex and geography effect in the whole sample.

Variation partitioning was then employed to evaluate the singular contribution and interaction of the four distinct components: taxonomy (described as the categorical variable “species”), size (described by lnCS), sex and geography on *Didelphis* skull shape variance [57,58]. These factors are all considered as predictors (X) of skull shape (Y, described by the 46 Relative Warp scores, 2n - 4 where n is the number of landmarks, [59]). We also performed variation partitioning on each species separately, to evaluate the relative contribution of size, sex and geography on skull shape variation. This analysis was performed with R package vegan 2.0 [59].

## Results

### Ecological Niche Modeling

All distribution models presented AUC values higher than 0.8 and positives BI values both for train and test data, indicating they both show a good degree of performance (S1 Fig).

**Fig 4:**
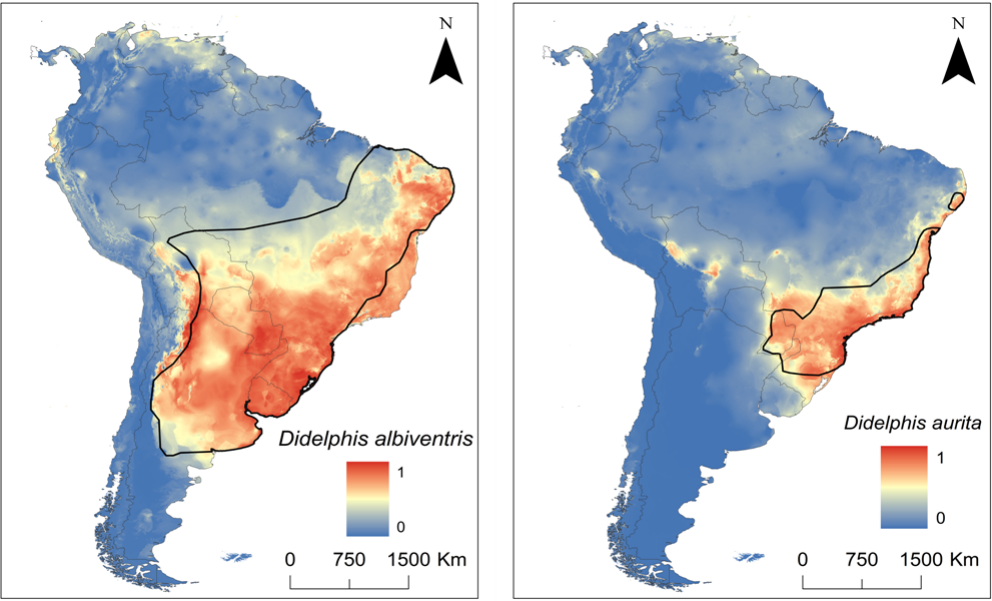
**Maps showing the climatic suitability for the White-eared Opossum, D. albiventris (at left), and for the Brazilian Common Opossum, *D*. *aurita* (at right) in South America based on an ensemble approach.** Warm colors indicate high climatic suitability (values close to one) and cold colors indicate low climatic suitability (values close to zero). The publicly available map layer was obtained from http://www.arcgis.com/features/features.html and the image prepared with the ArcMap 10 (ESRI Inc.). The polygons in each map corresponds to the IUCN map distribution for *Didelphis albiventris* and *D*. *aurita*.

*Didelphis albiventris* has a wider potential range than *D*. *aurita* occupying several areas such as the Cerrado, Atlantic forest, the Pampas, Chaco, and the Yungas (Fig 4). *Didelphis aurita* has a narrower potential range, including mostly the Atlantic forest but also the Cerrado southern boundaries (Fig 4). The *D* statistic for niche similarity in the South America indicates that there is a high similarity between species (*D* = 0.84, P = 0.02)

### Ecological Niche Similarity Analysis

**Fig 5:**
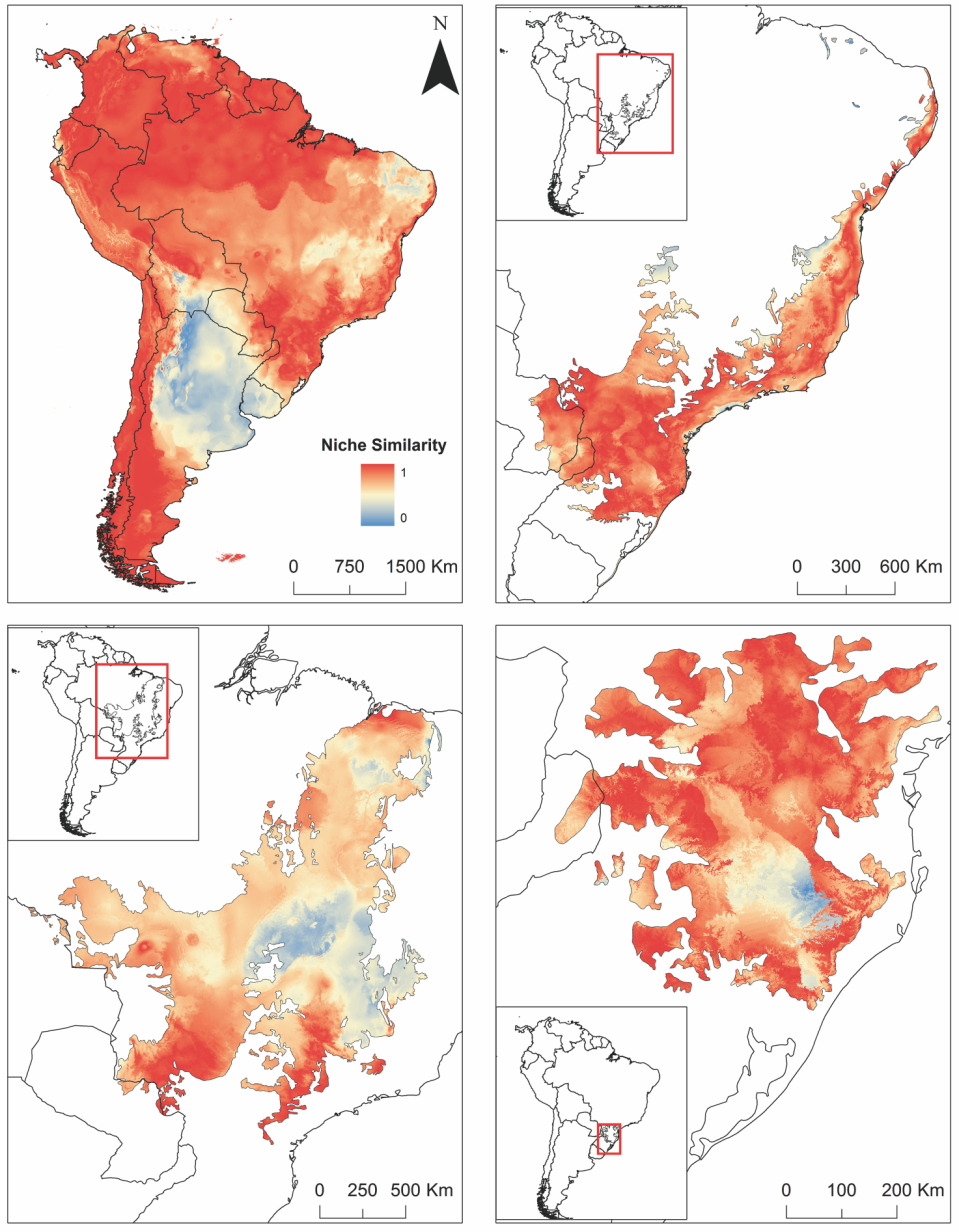
**Maps showing the level of niche similarity between the White-eared Opossum (*D*. *albiventris*) and the Brazilian Common Opossum (*D*. *aurita*) based on the S index in South America (top left), Atlantic forest (top right), Cerrado (bottom left) and in the Araucaria moist forest (bottom right).** Warm colors indicate high niche similarity (values close to one) and cold colors indicate low niche similarity (values close to zero). The publicly available map layers were obtained from http://www.arcgis.com/features/features.html and http://maps.tnc.org/gis_data.html and the image prepared with the ArcMap 10 (ESRI Inc.).

The Cerrado presents the lowest niche similarity index between species (*D* = 0.66, P = 0.02), being most of the eastern Cerrado unsuitable for *D*. *aurita* and suitable for *D*. *albiventris* (Figs 4 and 5). Niche similarity between species is higher in the Cerrado boundaries, especially in the southwestern and southeastern part of this biome (Fig 5). On the other hand, niche similarity in the Atlantic forest for *D*. *aurita* and *D*. *albiventris* is the highest (*D* = 0.94, P = 0.02) (Fig 5).

Even in potentially sympatric areas of the Atlantic forest where only *D*. *aurita* is expected to be present, such as the Araucaria moist forest, the climatic suitability of *D*. *albiventris* is classified as moderate to high. Climatic suitability in the southern part of both species ranges is high (Fig 5). Especially in the Araucaria moist forest, niche similarity between species is low in open areas which are climatically more suitable for *D*. *albiventris*. Indeed, *D*. *albiventris* tends to occur allopatrically in central and southern areas of this region (Fig 5).

In other parts of the Araucaria moist forest, the level of niche similarity between species is very high (*D* = 0.78, P = 0.02).

### Morphological Analyses

**Fig 6:**
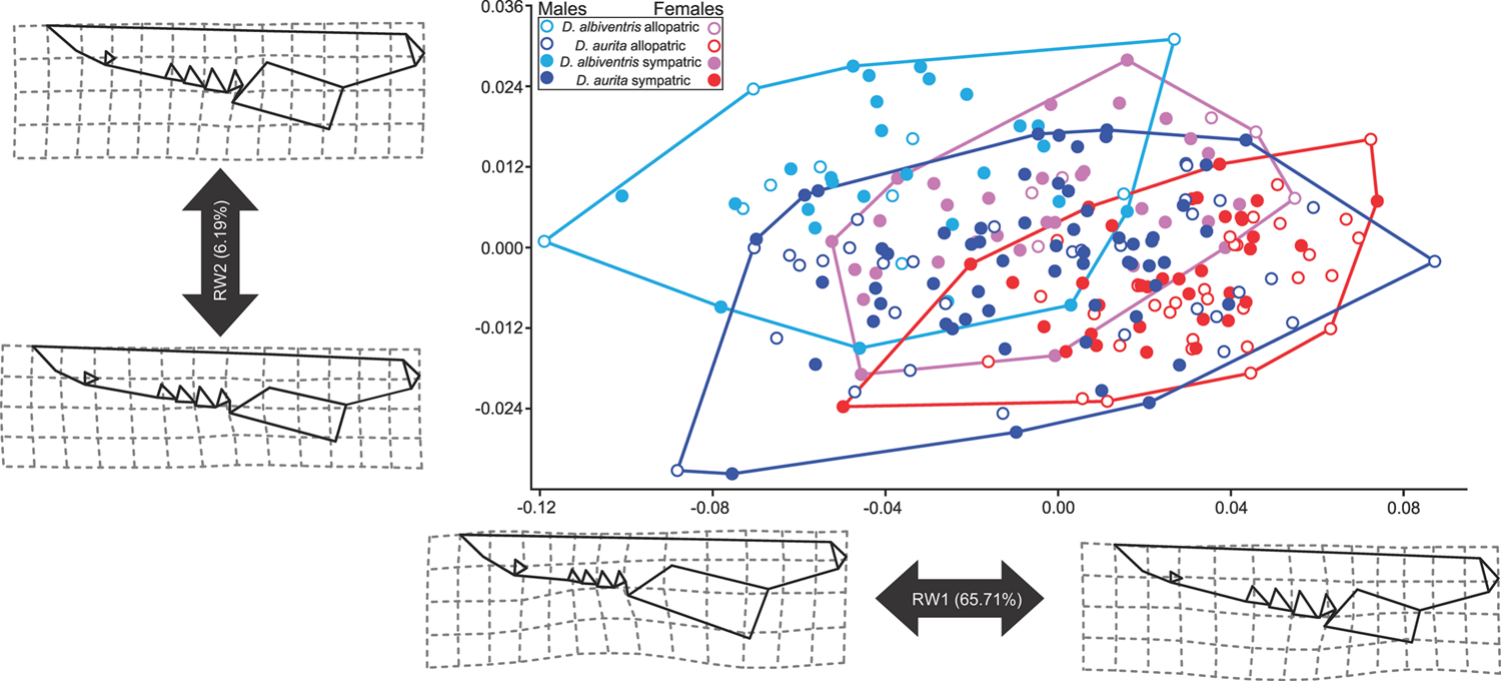
Scatter plot of RW1 versus RW2. Transformation grids visualize shape deformations relative to the mean at the positive and negative extremes of Relative Warps axes. Males and females from each species are labeled according to different colors within minimum convex hull superimposed. Allopatric and sympatric specimens are discriminated with unfilled and filled symbols respectively.

The first fifteen Relative Warps cumulatively explain 95% of total variance. The first (65.71% of shape variance) versus the second (6.19%) RWs plot evidence an extensive overlap between *Didelphis* species, as well as sexes, although some degree of separation is possible to notice (Fig 6). RW1 describes changes in the zygomatic arch, muzzle, occipital condyle and teeth. Specimens at the extreme negative of RW1 exhibit larger zygomatic arch and canine, as well as less elongated and thinner muzzle and smaller canine and occipital condyle as typical for males of *Didelphis*. On the positive scores specimens of both males and females overlap showing thinner zygomatic arch but relatively enlarged canine and molars. The RW2 describes shape changes related to overall skull shape, zygomatic arch, occipital condyle, teeth, and separates males of *D*. *albiventris* from the rest of the sample. On the negative scores of RW2 specimens exhibit thinner skull and zygomatic arch, relatively smaller occipital condyle, smaller molars and larger canine while broader skulls with larger teeth and condyle occur at the opposite extreme of RW2.

Species as well as sexes significantly differ in skull shape, with no interaction occurs between these two factors (Table 1). Geography is not a significant factor and does not show interaction with species or sex. *Didelphis albiventris* had no geography impact on skull shape but *D*. *aurita* does shift skull shape from allopatric to sympatric locations, although not strongly (R^2^ = 0.021, P < 0.038) (Table 1).

**Table 1:** Procrustes ANOVA results analyzing the skull shape variation in Didelphis according to: species (*D*. *albiventris* and *D*. *aurita*), sex, geography (allopatric and sympatric) and size (natural logarithm of centroid size).

		df	SS	MS	Rsq	F	P.val
All sample (n = 234)							
	Species	**1**	**0.055**	**0.055**	**0.108**	**33.984**	**0.005**
	Sex	**1**	**0.082**	**0.082**	**0.160**	**50.533**	**0.005**
	Species: Sex	1	0.001	0.001	0.002	0.592	0.725
	Residuals	230	0.373	0.002			
	Total	233	0.511	0.002			
	Species	**1**	**0.055**	**0.055**	**0.108**	**34.404**	**0.001**
	Sex	**1**	**0.082**	**0.082**	**0.160**	**51.157**	**0.001**
	Geography	1	0.003	0.003	0.006	2.028	0.206
	Species:Sex	1	0.001	0.001	0.002	0.600	0.769
	Species:Geography	1	0.001	0.001	0.003	0.823	0.562
	Sex:Geography	1	0.004	0.004	0.009	2.803	0.113
	Species:Sex:Geography	1	0.002	0.002	0.004	1.186	0.385
	Residuals	226	0.362	0.002			
	Total	233	0.511	0.002			
	Size	**1**	**0.097**	**0.097**	**0.189**	**82.980**	**0.005**
	Species	**1**	**0.135**	**0.135**	**0.264**	**115.667**	**0.005**
	Size: Species	**1**	**0.011**	**0.011**	**0.022**	**9.820**	**0.01**
	Residuals	230	0.268	0.001			
	Total	233	0.511	0.002			
	Size	**1**	**0.097**	**0.097**	**0.189**	**59.459**	**0.005**
	Sex	**1**	**0.031**	**0.031**	**0.060**	**18.976**	**0.005**
	Size: Sex	**1**	**0.009**	**0.009**	**0.018**	**5.748**	**0.015**
	Residuals	230	0.374	0.002			
	Total	233	0.511	0.002			
	Size	**1**	**0.097**	**0.097**	**0.189**	**57.237**	**0.005**
	Geography	**1**	**0.015**	**0.015**	**0.029**	**8.812**	**0.005**
	Size:Geography	**1**	**0.011**	**0.011**	**0.021**	**6.392**	**0.010**
	Residuals	230	0.389	0.002			
	Total	233	0.511	0.002			
*D*. *albiventris* (n = 75)							
	Size	**1**	**0.049**	**0.049**	**0.325**	**39.513**	**0.001**
	Sex	**1**	**0.012**	**0.012**	**0.082**	**9.983**	**0.002**
	Geography	1	0.001	0.001	0.006	0.773	0.711
	Size:Sex	1	0.001	0.001	0.007	0.858	0.650
	Size:Geography	1	0.003	0.003	0.020	2.422	0.183
	Sex:Geography	1	0.001	0.001	0.004	0.480	0.925
	Size:Sex:Geography	1	0.001	0.001	0.004	0.487	0.890
	Residuals	67	0.082	0.001			
	Total	74	0.149	0.002			
*D*. *aurita* (n = 159)							
	Sex	**1**	**0.139**	**0.139**	**0.454**	**143.655**	**0.001**
	Size	**1**	**0.010**	**0.010**	**0.031**	**9.848**	**0.010**
	Geography	**1**	**0.006**	**0.006**	**0.021**	**6.616**	**0.038**
	Sex:Size	1	0.002	0.002	0.006	2.033	0.347
	Sex:Geography	1	0.002	0.002	0.006	1.902	0.322
	Size:Geography	1	0.001	0.001	0.003	0.804	0.778
	Sex:Size:Geography	1	0.000	0.000	0.001	0.309	0.995
	Residuals	151	0.147	0.001			
	Total	158	0.307	0.002			

**Fig 7:**
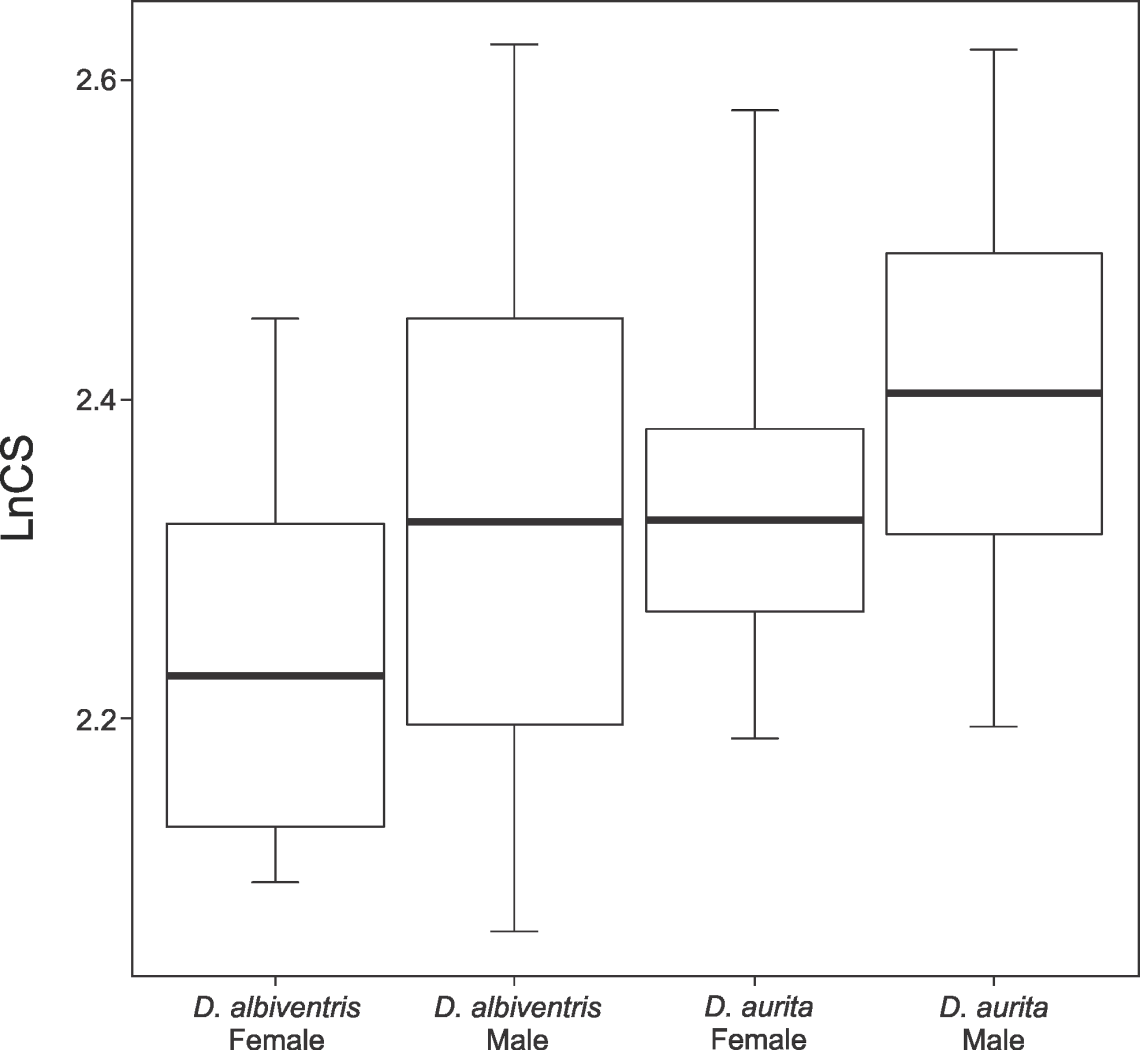
Boxplot showing the difference in natural log centroid size (lnCS) between males and females of *Didelphis albiventris* and *D*. *aurita*.

Skull size significantly differ between species (F = 59.803; P < 0.001) and sexes (F = 53.371; P < 0.001) (Fig 7) but not in geography (F = 0.822; P = 0.366). It provides a relatively large contribution (R^2^ = 0.189) on skull shape variance and its impact equally differ between species, sex and allopatric and sympatric specimens (Table 1).

**Fig 8:**
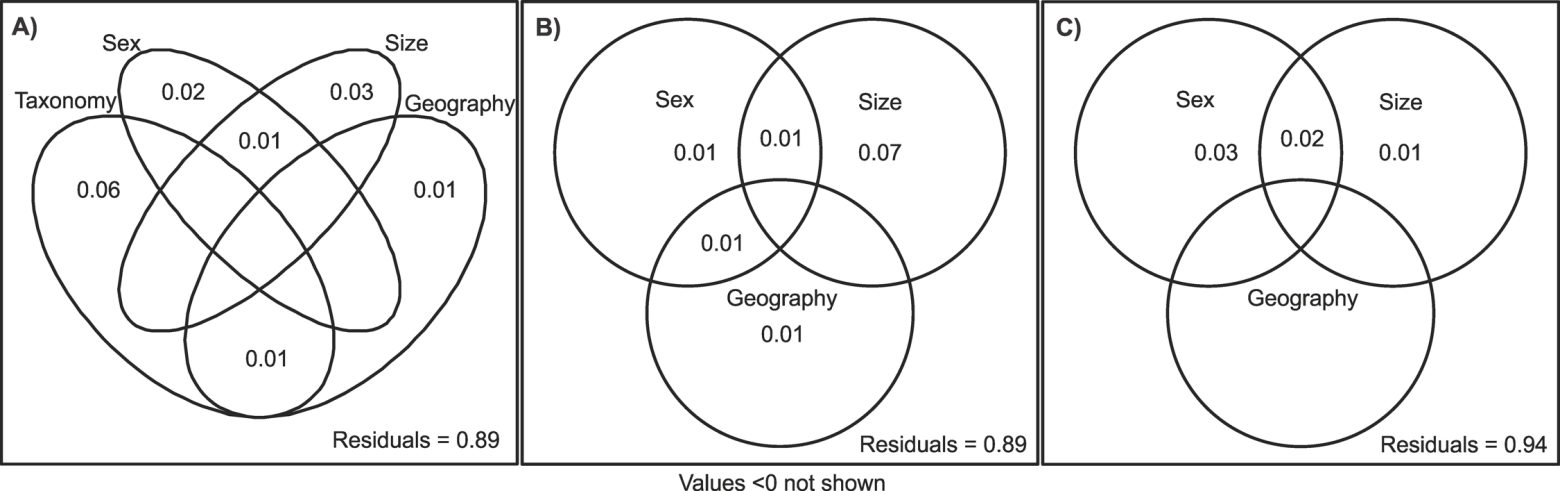
Schematic depiction of the factors analyzed in variation partition meant to illustrate both their individual contribution to *Didelphis* shape and their interaction components. A) *Didelphis* genera; B) *D*. *aurita*; C) *D*. *albiventris*.

Variation partitioning summarizes this: *Didelphis* skull shape is explained primarily by taxonomy as a single “pure” component (Adj R^2^ Taxonomy “Pure” = 0.059), followed by size (Adj R^2^ Size “Pure” = 0.033), sex (Adj R^2^ Sex “Pure” = 0.015) and geography (Adj R^2^ Size “Pure” = 0.006) (Fig 8A; S3 Table). The most important factor explaining *D*. *aurita* skull shape is size (Adj R^2^ Size “Pure” = 0.073), followed by sex and geography, which equally explain 1% of skull shape variation (Fig 8B, S4 Table). In *D*. *albiventris*, variation partitioning showed sex as the main factor explaining shape variance (Adj R^2^ Sex “Pure” = 0.035), followed by size (Adj R^2^ Size “Pure” = 0.015). Geography has no effect on this species (Fig 8C, S5 Table).

## Discussion

As we predicted, the habitat generalist *D*. *albiventris* showed potential to occur in most of the occurrence areas of the habitat specialist *D*. *aurita*. Therefore, it is plausible to suppose that if *D*. *aurita* were not distributed in the Atlantic forest and Cerrado, *D*. *albiventris* would occur throughout the Cerrado and the Atlantic forest, reaching the coastal vegetation eastward. Potentially, *D*. *albiventris* could be distributed in the middle and east of South America similarly to the crab-eating fox *Cerdocyon thous* (Canidae), which is also a habitat generalist species, occurring throughout the Cerrado and the Atlantic forest ecoregions [60]. The highest niche similarity was found in the Atlantic forest, suggesting that one species could occupy environments in both species ranges in this ecoregion, but their realized ranges did not overlap so widely in the Atlantic forest. The realized and potential ranges of *D*. *albiventris* show that it does not occur throughout the Atlantic forest because it finds a biotic barrier: the presence of *D*. *aurita*. For *Didelphis* in South America, there was at least one recent speciation event that split this genus into two clades: one habitat-generalist clade including *D*. *albiventris*, and a forest-specialist clade including *D*. *aurita*. The molecular differentiation between these two clades is about 5.7% [13]. Due to its current characteristics, *D*. *albiventris* should have originated by allopatric speciation in savannah-like refuges. ENM suggests that *D*. *albiventris* can potentially occur in all eastern South America, including the coastal Atlantic forest but today it does not occur there. As an explanation, we suggest some level of interference competition between *D*. *albiventris* and *D*. *aurita* in the Atlantic forest, which would have led to a competitive exclusion of the former species rather than competitive release [2].When in sympatry in the Araucaria moist forest, *D*. *aurita* occupies mostly forested areas while *D*. *albiventris* occupies only marginal habitats, which could be a case of interference competition [16].The same pattern was observed in two species of mouse opossums (genus *Marmosa*) in northern South America, where one species (*Marmosa xerophila*) possibly competes with the other (peninsular *M*. *robinsoni*), with the last one shifting its distribution in relation to what it could potentially occupy [3]. We do not know for sure which species, or clade, of *Didelphis* emerged first. If the generalist clade originated first (through the initial appearance of *D*. *albiventris*-*D*. *virginiana* clade, [61]), then the occupation of the Atlantic forest by the specialized clade would have occurred later, with the appearance and subsequent irradiation of *D*. *aurita* displacing *D*. *albiventris* from the most forested regions, resulting in what we see today as their realized distributions: *D*. *albiventris* outside the central and eastern parts of the Atlantic forest. Currently *D*. *aurita* is a forest dweller species, depending on forested and generally more humid areas, as its sister species, *D*. *marsupialis* that occupies the wetter Amazon forests [13,14,15].

The potential distribution of *D*. *albiventris* extends southward the Atlantic forest, within the Araucaria moist forest. Specifically in the Araucaria moist forest, both species of *Didelphis* widely overlap, since this ecoregion has elements that meet the ecological niche of both species (forests and grasslands). Then, we are able to detail sympatric and allopatric zones of these two species in the Araucaria moist forest. There are areas of the Araucaria moist forest that are more climatically suitable for *D*. *albiventris* than for *D*. *aurita*, such as where the Araucaria moist forest connects to the Pampas. The Araucaria moist forest presents both forest and grassland patches, which makes it suitable for both opossum species at a landscape scale. In fact, potential distribution for both species overlaps largely in the Araucaria moist forest, providing a high niche similarity *D* statistic. Habitat patches distribution may favor a generalist species locally, especially when the habitat is grassland which limits *D*. *aurita* and favors *D*. *albiventris*. This might explain why these species coexist when there is a mosaic of forest-grassland [16] and not in the tropical Atlantic forest.

The morphometric analyses show a degree of shape discrimination between the species that possibly allowed them to occupy marginally different feeding niches supplemented by behavioral shift in contact areas. *Didelphis albiventris* shows relatively broader skull at the zygoma while *D*. *aurita* specimens had wider teeth but thinner zygomatic arch. Those features also mirror different sexes with males of *D*. *albiventris* being more differentiated by their relatively larger zygoma than females of both species as well as males of *D*. *aurita*. Sexual dimorphism is relatively higher in *D*. *albiventris* (around 3% of shape variance) than in *D*. *aurita* although significant all the time. Clearly, size differentiation seems to play a role into shape differences and its contribution varies with *D*. *aurita* being more influenced by allometry. This might reflect the result of long time of divergence and sexual segregation between both species. Males having wider zygoma should have stronger bite force and access to broader range of food than females (not surprisingly males are larger than females in both species). A lot of overlap is shown between sympatric and allopatric specimens (Fig 6) and our analyses clarified that a significant shift in shape occurs only in *D*. *aurita* in sympatric areas (the more specialized competitor). This could be a residual evidence of a higher past competition between both species [62], when possibly contact zones were larger than today. It also supports the hypothesis that *D*. *albiventris* is ecologically more tolerant and flexible than *D*. *aurita* whose morphological shift could be a consequence of differential foraging and spatial behavior when in sympatry [16]. Both sexes of *D*. *albiventris* and male *D*. *aurita* indeed have different diets: they are both omnivorous generalist with female *D*. *aurita* including more soft food items like flesh fruits and soil invertebrates [16,63,64]. As a general result of our analyses, at biome and ecoregion scales, *D*. *aurita* seems to act as a biotic barrier for the colonization of some regions of the Atlantic forest by *D*. *albiventris*.

This is another example where biotic interactions can lead to different patterns of species distributions (see [3]). The potential distribution of *D*. *aurita* coincides with the distribution of the Atlantic forest and the potential distribution of *D*. *albiventris* extends beyond the realized range, just in the places where *D*. *aurita* exists. Our results lead us to hypothesize that these species compete in their overlapping zones and that *D*. *albiventris* may have replaced *D*. *aurita* by competitive exclusion [2] if populations of *D*. *aurita* are becoming unstable due to climatic and/or environmental disturbances (see [17]). This is thought to be true at least for the Araucaria moist forest where *D*. *aurita* exists in mosaic with *D*. *albiventris* [22]. This type of analysis using ENM should be more explored to better understand why species have their extant distributions [5].

Thus, the specialist *D*. *aurita* acts a biotic barrier to *D*. *albiventris* when niche diversity is not available for coexistence. On the other hand, when there is niche diversification (e.g. habitat mosaic), both species are capable to coexist with a minimal competitive effect in *D*. *aurita* morphological traits.

## Supporting Information

S1 Appendix Ecological Niche Models comparison analyses procedures to test for coordinates accuracy and error. (DOCX)

S1 Fig **Boxplot with AUC and Boyce Index (BI) values of both train (at left) and test data (at right) for each algorithm used to build the niche models for the White-eared Opossum (*Didelphis albiventris*) and the Brazilian Common Opossum (*D*. *aurita*).** Dark bar indicates the mean, box represents the standard deviation and whiskers represent the maximum and minimum values. ED = Euclidean Distance; MAX = Maximum Entropy; SVM = Support Vector Machine.
(TIF)

S2 Fig **Maps showing the climatic suitability for the White-eared Opossum, *D*. *albiventris* (at the top), and for the Brazilian Common Opossum, *D*. *aurita* (at bottom) in South America based on an ensemble approach showing differences in the prediction when using only GPS data (at left) and using all data available (at right).** Green indicates high climatic suitability (values close to one) and white indicates low climatic suitability (values close to zero). The publicly available map layer was obtained from http://www.arcgis.com/features/features.html and the image was prepared in the R environment (https://cran.r-project.org/) using the package raster [34].
(TIF)

S1 Table *Didelphis albiventris* and *D*. *aurita* records, separated by species, source and coordinates. (DOCX)

S2 Table *Didelphis albiventris* and *D*. *aurita* specimens used for morphological analyses, separated by museum record, species, sex, geographical coordinates (in decimal degrees) and geography. (DOCX)

S3 Table Variation partitioning with *Didelphis* skull shape as dependent variable and taxonomy, size, sex and geography as dependent variables. P values tests for the significance of F after 1000 permutations. Significance is highlighted.
(DOCX)

S4 Table Variation partitioning with *Didelphis aurita* skull shape as dependent variable and size, sex and geography as dependent variables. P values tests for the significance of F after 1000 permutations. Significance is highlighted.
(DOCX)

S5 Table Variation partitioning with *Didelphis albiventris* skull shape as dependent variable and size, sex and geography as dependent variables. P values tests for the significance of F after 1000 permutations. Significance is highlighted.
(DOCX)

S6 Table Full morphometric data used in this study. (XLSX)
