# Supplementary material for: Which Factors Determine Spatial Segregation in the South American Opossums (Didelphis aurita and D. albiventris)? An Ecological Niche Modelling and Geometric Morphometrics Approach
Source: PLoS One. 2016 Jun 23;11(6):e0157723. doi: 10.1371/journal.pone.0157723 (PMC4919065; doi:10.1371/journal.pone.0157723)
Supplement: S1 Appendix — (DOCX) [file pone.0157723.s001.docx]

**S1 Appendix. Ecological Niche Models comparison analyses procedures to test for coordinates accuracy and error**

In order to analyze how coordinate precision could affect our predictions, we ran ecological niche models using only occurrence data georeferenced locally with a GPS obtained in the field by our research group. These data are less prone to coarse errors when georeferecing. In general GPS errors are very small considering the scale we are working (e.g. less than 1 km). We have 109 records for the White-eared Opossum, *Didelphis albiventris*), and 72 records for the Brazilian Common Opossum, *Didelphis aurita*. To run the ENMs we followed the same protocol as described in the Material and Methods section. Summarizing, (i) thinning species occurrence to make occurrence points independent, (ii) use of three algorithms, (iii) and creation of a consensual model (ensemble) with the individual models evaluated as good to excellent (AUC≥ 0.75 and Boyce Index ≥0.25).

Then we correlated the environmental suitability values from the models using GPS data (hereafter GPS models) with the environmental suitability from the models using all available data (hereafter whole model). If data accuracy affects model prediction, we should find low correlations and our predictions should not be used with a fine resolution (1 km). On the other hand, if the correlation is high there should be no big difference between a GPS model and a whole model. Results of the GPS and whole models for both species can be visualized in the S2 Figure. We found a correlation between models of r = 0.93 (p<0.0001) for *D. aurita* and r = 0.69 (p<0.0001) for *D. albiventris*. Therefore, in this case all analyses derived from the ENM should not be affected by coordinate accuracy and we keep the original models (whole models). However, it is worth mentioning that coordinate precision should be evaluated whenever possible to create better models.

**References**

Hijmans, R.J. (2015). raster: Geographic Data Analysis and Modeling. R package version 2.4-20. Available: http://CRAN.R-project.org/package=raster
