# Supplementary figures and images for: Which Factors Determine Spatial Segregation in the South American Opossums (Didelphis aurita and D. albiventris)? An Ecological Niche Modelling and Geometric Morphometrics Approach
Source: PLoS One. 2016 Jun 23;11(6):e0157723. doi: 10.1371/journal.pone.0157723 (PMC4919065; doi:10.1371/journal.pone.0157723)

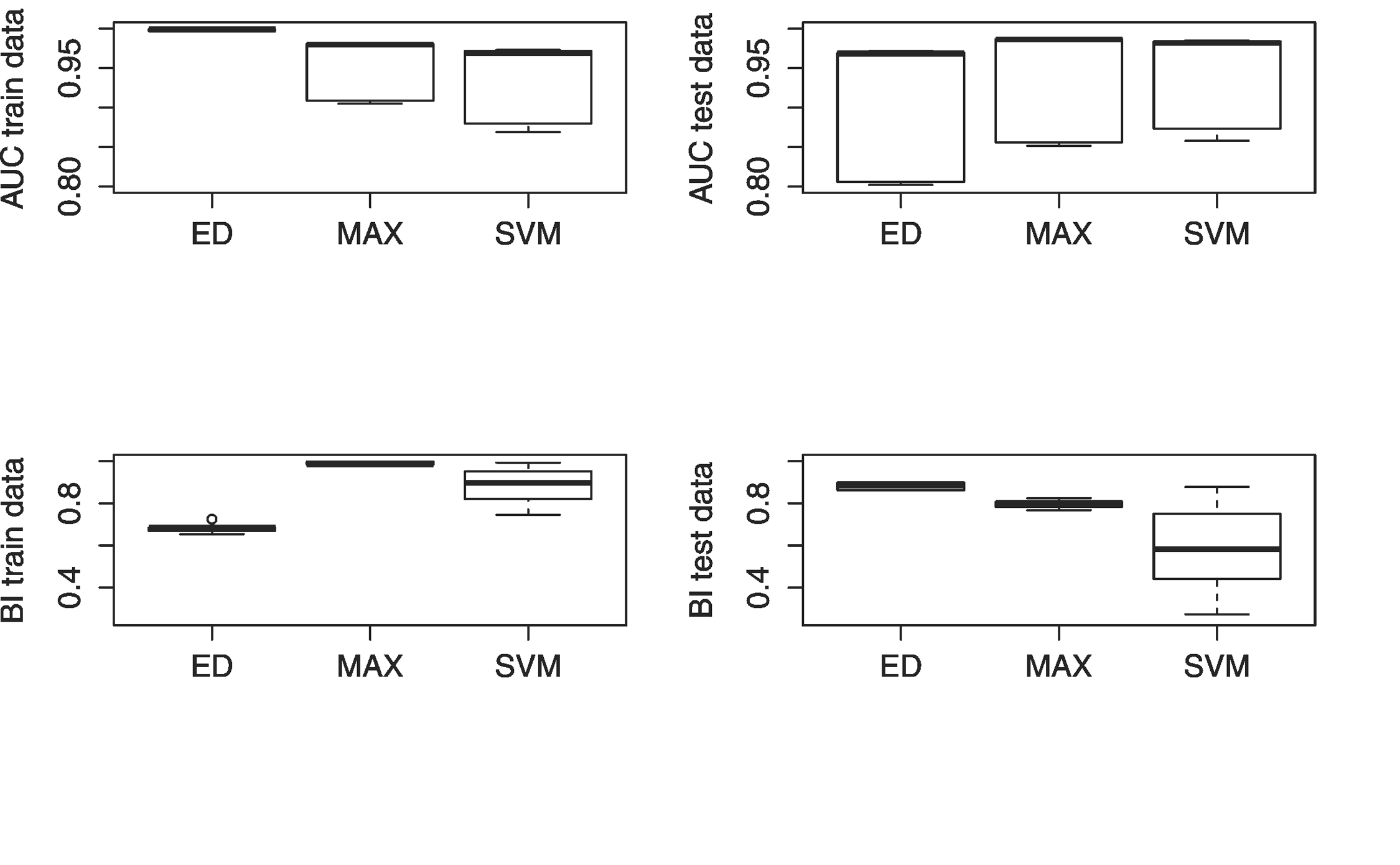

Supplement: S1 Fig — Boxplot with AUC and Boyce Index (BI) values of both train (at left) and test data (at right) for each algorithm used to build the niche models for the White-eared Opossum (Didelphis albiventris) and the Brazilian Common Opossum (D. aurita). Dark bar indicates the mean, box represents the standard deviation and whiskers represent the maximum and minimum values. ED = Euclidean Distance; MAX = Maximum Entropy; SVM = Support Vector Machine. (TIF) [file pone.0157723.s002.TIF]

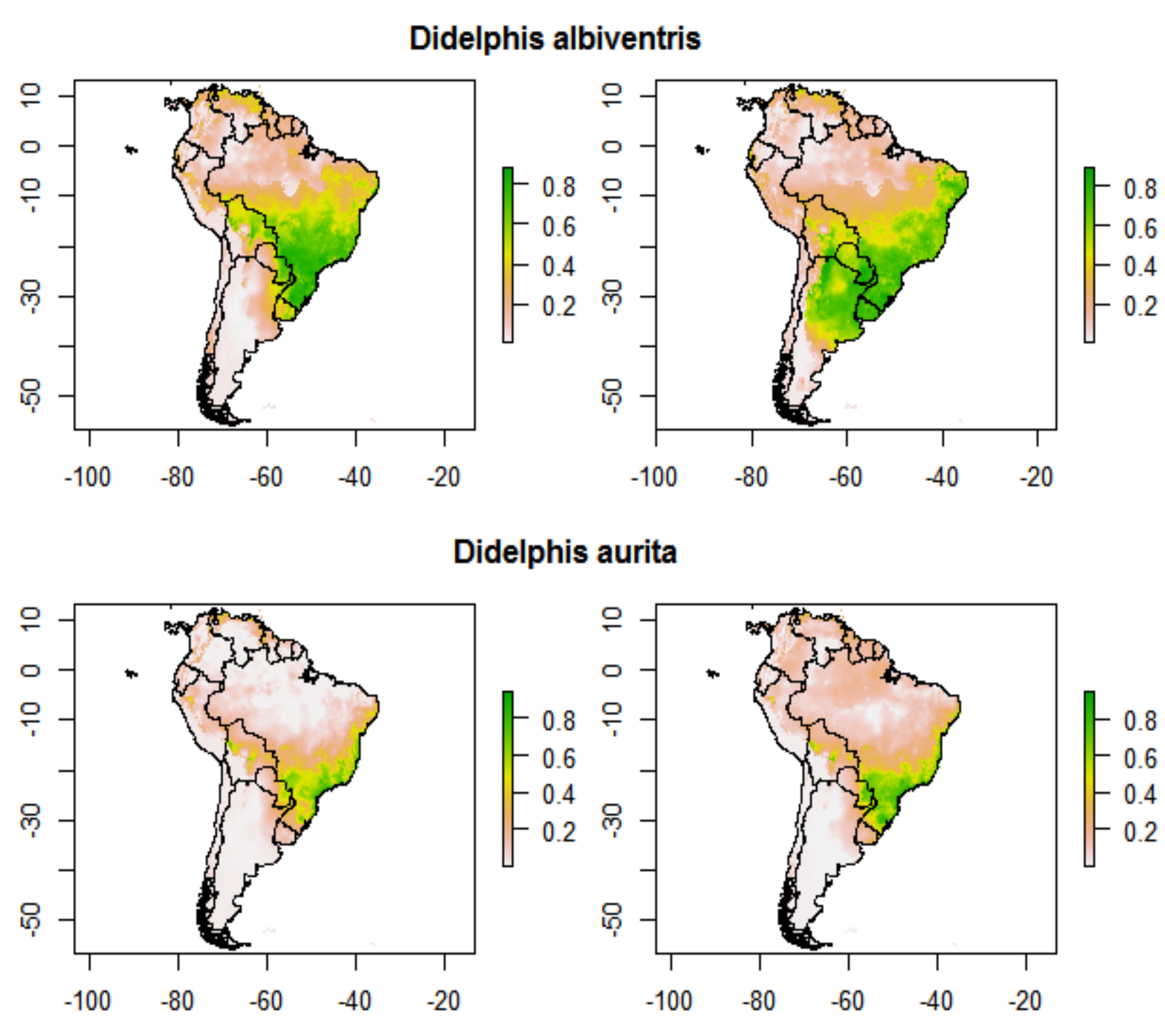

Supplement: S2 Fig — Maps showing the climatic suitability for the White-eared Opossum, D. albiventris (at the top), and for the Brazilian Common Opossum, D. aurita (at bottom) in South America based on an ensemble approach showing differences in the prediction when using only GPS data (at left) and using all data available (at right). Green indicates high climatic suitability (values close to one) and white indicates low climatic suitability (values close to zero). The publicly available map layer was obtained from http://www.arcgis.com/features/features.html and the image was prepared in the R environment (https://cran.r-project.org/) using the package raster [34]. (TIF) [file pone.0157723.s003.tif]
