# Supplementary material for: Which Factors Determine Spatial Segregation in the South American Opossums (Didelphis aurita and D. albiventris)? An Ecological Niche Modelling and Geometric Morphometrics Approach
Source: PLoS One. 2016 Jun 23;11(6):e0157723. doi: 10.1371/journal.pone.0157723 (PMC4919065; doi:10.1371/journal.pone.0157723)
Supplement: S1 Table — (DOCX) [file pone.0157723.s004.docx]

**S1 Table.** *Didelphis albiventris* and *D. aurita* records, separated by species, source and coordinates (in degrees). *Specimen available at the Museu Nacional (RJ) without museum registration number.

| Species | Latitude | Longitude | Source | |
| --- | --- | --- | --- | --- |
| *D. albiventris* | 9° 44' 17.71'' S | 36° 30' 11.99'' W | PI 3752* |  |
| *D. albiventris* | 9° 24' 53.52'' S | 36° 37' 53.67'' W | MN 22873 |  |
| *D. albiventris* | 9° 9' 29.18'' S | 35° 17' 45.85'' W | MN 28596 |  |
| *D. albiventris* | 9° 19' 32.06'' S | 36° 28' 21.43'' W | MN 22878 |  |
| *D. albiventris* | 9° 0' 7.91'' S | 35° 50' 18.15'' W |  | [1] |
| *D. albiventris* | 9° 21' 48.16'' S | 36° 14' 19.02'' W | MN 22881 |  |
| *D. albiventris* | 11° 42' 45.30'' S | 40° 37' 32.97'' W |  | [2] |
| *D. albiventris* | 12° 8' 51.89'' S | 44° 59' 42.39'' W | MN 4248 |  |
| *D. albiventris* | 14° 57' 27.56'' S | 39° 33' 19.74'' W | MN 10023 |  |
| *D. albiventris* | 8° 59' 3.44'' S | 39° 54' 4.84'' W |  | [3] |
| *D. albiventris* | 12° 15' 23.83'' S | 38° 37' 6.44'' W | MN 7235 | [4] |
| *D. albiventris* | 11° 2' 18.95'' S | 45° 10' 42.85'' W |  | [5] |
| *D. albiventris* | 14° 47' 19.94'' S | 39° 16' 44.95'' W | MN 31421 |  |
| *D. albiventris* | 12° 54' 21.12'' S | 41° 6' 35.72'' W | MN 20390 | [6] |
| *D. albiventris* | 13° 35' 27.69'' S | 44° 32' 7.54'' W | MN 53666 |  |
| *D. albiventris* | 13° 31' 43.38'' S | 39° 58' 36.42'' W | MN 17225 |  |
| *D. albiventris* | 13° 51' 3.76'' S | 40° 4' 52.22'' W | MN 22927 |  |
| *D. albiventris* | 15° 34' 11.28'' S | 40° 1' 10.17'' W | MN 4253 |  |
| *D. albiventris* | 12° 33' 25.65'' S | 41° 23' 31.40'' W | MZUSP 33826 | [2] |
| *D. albiventris* | 12° 31' 46.06'' S | 41° 34' 27.74'' W | MN 22936 |  |
| *D. albiventris* | 14° 49' 49.55'' S | 41° 23' 14.77'' W | MN 22887 |  |
| *D. albiventris* | 10° 27' 57.40'' S | 40° 10' 50.88'' W | MZUSP 2605 |  |
| *D. albiventris* | 11° 37' 46.56'' S | 38° 58' 39.85'' W | MN 22942 |  |
| *D. albiventris* | 9° 28' 31.67'' S | 40° 48' 50.21'' W | MN 23658 |  |
| *D. albiventris* | 14° 51' 5.22'' S | 40° 50' 54.61'' W | MN 8322 |  |
| *D. albiventris* | 7° 29' 26.63'' S | 38° 59' 7.65'' W | MN 26020 |  |
| *D. albiventris* | 7° 3' 32.47'' S | 40° 21' 5.27'' W | MN 26021 |  |
| *D. albiventris* | 3° 5' 1.18'' S | 39° 40' 59.88'' W | MZUSP 8700 |  |
| *D. albiventris* | 7° 13' 59.13'' S | 39° 24' 59.82'' W | MN 1481 |  |
| *D. albiventris* | 4° 19' 41.36'' S | 40° 42' 37.77'' W | MN 23571 |  |
| *D. albiventris* | 3° 41' 19.75'' S | 39° 35' 8.88'' W | MN 23632 |  |
| *D. albiventris* | 7° 35' 2.68'' S | 39° 16' 50.09'' W | W 1662* |  |
| *D. albiventris* | 7° 21' 20.58'' S | 38° 57' 27.39'' W | MN 26018 |  |
| *D. albiventris* | 7° 14' 58.88'' S | 39° 8' 43.73'' W | MN 23626 |  |
| *D. albiventris* | 4° 13' 28.79'' S | 38° 55' 31.67'' W | MN 23810 |  |
| *D. albiventris* | 4° 2' 42.20'' S | 40° 52' 1.68'' W | MN 23815 |  |
| *D. albiventris* | 3° 50' 6.12'' S | 40° 53' 52.00'' W |  | [7] |
| *D. albiventris* | 15° 56' 41.00'' S | 47° 53' 7.00'' W |  | [8] |
| *D. albiventris* | 16° 19' 43.33'' S | 48° 57' 12.23'' W | MN 4740 |  |
| *D. albiventris* | 15° 54' 6.85'' S | 52° 14' 43.56'' W |  | [9] |
| *D. albiventris* | 16° 10' 52.05'' S | 52° 32' 34.43'' W |  | [10] |
| *D. albiventris* | 16° 50' 38.04'' S | 51° 46' 14.74'' W |  | [9] |
| *D. albiventris* | 17° 44' 44.46'' S | 48° 37' 32.84'' W | MN 20960 |  |
| *D. albiventris* | 13° 0' 1.00'' S | 46° 20' 25.00'' W | MN 10499 |  |
| *D. albiventris* | 13° 44' 25.22'' S | 46° 52' 52.28'' W | MZUSP 4133 |  |
| *D. albiventris* | 13° 47' 51.40'' S | 47° 27' 19.80'' W | MN 46514 |  |
| *D. albiventris* | 15° 32' 3.05'' S | 47° 19' 57.03'' W | MN 24446 |  |
| *D. albiventris* | 17° 43' 16.61'' S | 48° 9' 47.38'' W | MN 37816 | [11] |
| *D. albiventris* | 13° 32' 16.95'' S | 48° 13' 6.43'' W | UNB 387 |  |
| *D. albiventris* | 17° 38' 26.58'' S | 52° 48' 6.11'' W |  | [9] |
| *D. albiventris* | 16° 27' 43.27'' S | 51° 46' 5.46'' W |  | [9] |
| *D. albiventris* | 13° 46' 49.85'' S | 47° 15' 28.21'' W | MN 42991 |  |
| *D. albiventris* | 14° 26' 2.90'' S | 49° 42' 31.73'' W |  |  |
| *D. albiventris* | 13° 34' 4.63'' S | 47° 12' 22.18'' W |  | [12] |
| *D. albiventris* | 14° 25' 56.61'' S | 49° 42' 52.65'' W | MN 43054 | [6] |
| *D. albiventris* | 14° 1' 59.85'' S | 48° 13' 44.95'' W | MN 36248 | [11] |
| *D. albiventris* | 5° 41' 16.08'' S | 47° 22' 26.37'' W | MHNCI 3905 |  |
| *D. albiventris* | 6° 30' 43.45'' S | 43° 42' 10.46'' W |  | [13] |
| *D. albiventris* | 20° 1' 16.95'' S | 45° 57' 38.74'' W | DZUFMG 710 | [6] |
| *D. albiventris* | 19° 57' 3.80'' S | 43° 54' 17.92'' W |  | [14] |
| *D. albiventris* | 16° 33' 53.40'' S | 45° 58' 47.32'' W | DZUFMG 654 | [6] |
| *D. albiventris* | 17° 42' 20.40'' S | 40° 45' 47.03'' W | MZUSP 4096 |  |
| *D. albiventris* | 19° 52' 50.15'' S | 43° 40' 11.11'' W | DZUFMG 617 | [6] |
| *D. albiventris* | 19° 56' 18.82'' S | 44° 3' 10.58'' W | DZUFMG 708 | [6] |
| *D. albiventris* | 15° 20' 21.27'' S | 43° 40' 36.96'' W | MN 43812 |  |
| *D. albiventris* | 16° 26' 7.62'' S | 41° 0' 11.87'' W | DZUFMG 905 | [6] |
| *D. albiventris* | 19° 37' 44.67'' S | 43° 53' 23.09'' W | MN 4229 |  |
| *D. albiventris* | 17° 53' 14.04'' S | 44° 34' 53.39'' W | DZUFMG 647 | [6] |
| *D. albiventris* | 19° 47' 0.35'' S | 45° 40' 53.12'' W | DZUFMG 846 | [6] |
| *D. albiventris* | 19° 16' 54.30'' S | 44° 24' 29.43'' W | MN 1189 |  |
| *D. albiventris* | 20° 43' 15.04'' S | 46° 36' 35.71'' W | MN 23642 |  |
| *D. albiventris* | 19° 37' 12.41'' S | 44° 2' 37.60'' W | DZUFMG 846 | [6] |
| *D. albiventris* | 17° 20' 37.12'' S | 44° 53' 47.57'' W | MZUSP 3111 |  |
| *D. albiventris* | 22° 13' 1.91'' S | 45° 56' 9.00'' W |  | [13] |
| *D. albiventris* | 18° 39' 0.59'' S | 44° 3' 27.65'' W | DZUFMG 640 | [6] |
| *D. albiventris* | 19° 28' 41.88'' S | 44° 9' 27.17'' W | DZUFMG 587 | [6] |
| *D. albiventris* | 19° 53' 21.41'' S | 43° 48' 18.03'' W | DZUFMG 816 | [6] |
| *D. albiventris* | 15° 24' 0.00'' S | 43° 10' 0.00'' W | MN 42834 |  |
| *D. albiventris* | 20° 6' 14.00'' S | 43° 28' 32.00'' W | DZUFMG 833 | [6] |
| *D. albiventris* | 19° 46' 1.93'' S | 43° 51' 8.56'' W | DZUFMG 120 | [6] |
| *D. albiventris* | 20° 54' 50.47'' S | 44° 50' 23.68'' W |  | [15] |
| *D. albiventris* | 19° 49' 22.25'' S | 43° 21' 32.34'' W | DZUFMG 630 | [6] |
| *D. albiventris* | 19° 28' 4.47'' S | 44° 14' 51.54'' W | DZUFMG 582 | [6] |
| *D. albiventris* | 18° 36' 11.85'' S | 48° 41' 24.66'' W | DZUFMG 766 | [6] |
| *D. albiventris* | 22° 18' 12.90'' S | 53° 16' 15.42'' W | MZUSP 28803 |  |
| *D. albiventris* | 22° 4' 18.00'' S | 56° 32' 49.90'' W | Personal information | Cáceres N.C. |
| *D. albiventris* | 20° 52' 5.35'' S | 56° 46' 48.47'' W |  | [16] |
| *D. albiventris* | 20° 44' 50.49'' S | 56° 24' 43.26'' W |  | Melo GL, Sponchiado J |
| *D. albiventris* | 21° 12' 59.97'' S | 56° 17' 53.52'' W |  | Melo GL, Sponchiado J |
| *D. albiventris* | 20° 26' 36.72'' S | 54° 38' 51.93'' W | MZUSP 3779 |  |
| *D. albiventris* | 20° 29' 8.50'' S | 54° 30' 4.90'' W | Personal information | Cáceres NC |
| *D. albiventris* | 20° 29' 56.07'' S | 54° 29' 42.63'' W |  | [9] |
| *D. albiventris* | 19° 28' 48.00'' S | 56° 55' 4.50'' W | Personal information | Cáceres NC |
| *D. albiventris* | 19° 11' 58.00'' S | 57° 37' 44.00'' W |  | [17] |
| *D. albiventris* | 19° 12' 5.84'' S | 57° 34' 6.43'' W |  | [17] |
| *D. albiventris* | 19° 23' 37.70'' S | 57° 23' 46.40'' W | Personal information | Cáceres NC |
| *D. albiventris* | 18° 59' 9.74'' S | 56° 59' 0.36'' W |  | [18] |
| *D. albiventris* | 18° 39' 18.40'' S | 52° 55' 27.27'' W |  | [9] |
| *D. albiventris* | 20° 34' 6.98'' S | 55° 18' 18.35'' W |  | [17] |
| *D. albiventris* | 26° 6' 1.10'' S | 54° 35' 13.30'' W |  | Cáceres NC |
| *D. albiventris* | 23° 47' 9.40'' S | 54° 7' 31.20'' W |  | Cáceres NC |
| *D. albiventris* | 22° 50' 31.00'' S | 55° 0' 2.00'' W |  | Cáceres NC |
| *D. albiventris* | 20° 46' 13.85'' S | 55° 52' 16.74'' W | Personal information | Melo GL, Sponchiado J |
| *D. albiventris* | 21° 26' 44.38'' S | 54° 26' 8.35'' W |  | [9] |
| *D. albiventris* | 21° 35' 17.90'' S | 53° 52' 6.20'' W | Personal information | Cáceres NC |
| *D. albiventris* | 19° 33' 10.81'' S | 51° 51' 59.22'' W |  | [9] |
| *D. albiventris* | 21° 41' 18.69'' S | 52° 36' 4.24'' W |  | [9] |
| *D. albiventris* | 20° 45' 31.20'' S | 54° 50' 35.00'' W |  | Cáceres NC |
| *D. albiventris* | 20° 27' 3.86'' S | 55° 16' 30.41'' W |  | [17] |
| *D. albiventris* | 20° 26' 20.92'' S | 56° 12' 17.60'' W | Personal information | Melo GL, Sponchiado J |
| *D. albiventris* | 20° 21' 16.43'' S | 55° 13' 26.27'' W | Personal information | Melo GL, Sponchiado J |
| *D. albiventris* | 20° 39' 53.06'' S | 55° 0' 52.14'' W | Personal information | Melo GL, Sponchiado J |
| *D. albiventris* | 20° 54' 13.70'' S | 52° 10' 30.00'' W |  | Cáceres NC |
| *D. albiventris* | 20° 49' 53.21'' S | 51° 41' 2.48'' W |  | [9] |
| *D. albiventris* | 17° 16' 59.15'' S | 53° 14' 57.81'' W |  | [9] |
| *D. albiventris* | 15° 52' 34.24'' S | 52° 9' 41.33'' W |  | [9] |
| *D. albiventris* | 16° 14' 29.96'' S | 57° 33' 1.32'' W |  | [19] |
| *D. albiventris* | 14° 53' 17.66'' S | 55° 43' 1.69'' W | UNB 615 | [6] |
| *D. albiventris* | 18° 30' 17.05'' S | 54° 44' 38.64'' W | MN 1187 |  |
| *D. albiventris* | 21° 37' 8.49'' S | 55° 9' 57.94'' W | MN 24956 |  |
| *D. albiventris* | 16° 44' 57.18'' S | 52° 49' 39.00'' W |  | [10] |
| *D. albiventris* | 13° 51' 27.05'' S | 53° 3' 28.64'' W | MZUSP 7039 |  |
| *D. albiventris* | 13° 30' 52.79'' S | 51° 23' 4.03'' W |  | [13] |
| *D. albiventris* | 13° 2' 54.12'' S | 58° 17' 45.26'' W |  | [13] |
| *D. albiventris* | 6° 39' 0.22'' S | 35° 7' 59.96'' W | MZUSP 8452 |  |
| *D. albiventris* | 7° 44' 0.40'' S | 37° 59' 22.98'' W | MN 46523 |  |
| *D. albiventris* | 8° 40' 0.01'' S | 35° 43' 0.01'' W | MN 17231 |  |
| *D. albiventris* | 7° 46' 15.35'' S | 39° 55' 41.33'' W | MN 30156 |  |
| *D. albiventris* | 9° 9' 51.15'' S | 36° 40' 59.96'' W | MN 23586 |  |
| *D. albiventris* | 8° 4' 60.00'' S | 35° 4' 60.00'' W |  | [1] |
| *D. albiventris* | 8° 16' 50.92'' S | 35° 58' 31.59'' W |  | [4] |
| *D. albiventris* | 7° 30' 58.17'' S | 39° 43' 19.91'' W | MZUSP 16533 |  |
| *D. albiventris* | 8° 53' 47.84'' S | 36° 30' 21.92'' W | MN 23654 |  |
| *D. albiventris* | 8° 53' 0.24'' S | 36° 29' 2.11'' W |  | [4] |
| *D. albiventris* | 8° 42' 37.00'' S | 35° 50' 1.00'' W |  | [1] |
| *D. albiventris* | 7° 52' 37.14'' S | 40° 4' 49.05'' W | MN 75041 |  |
| *D. albiventris* | 8° 32' 2.44'' S | 36° 57' 52.27'' W |  | [4] |
| *D. albiventris* | 8° 22' 17.81'' S | 36° 41' 30.51'' W | MN 23587 |  |
| *D. albiventris* | 8° 11' 18.98'' S | 36° 42' 30.68'' W | MN 1502 |  |
| *D. albiventris* | 8° 0' 36.07'' S | 34° 56' 51.38'' W |  | [20] |
| *D. albiventris* | 24° 38' 60.00'' S | 48° 58' 60.00'' W | MHNCI 2659 |  |
| *D. albiventris* | 24° 43' 0.00'' S | 52° 1' 60.00'' W | MHNCI 4198 |  |
| *D. albiventris* | 25° 22' 0.00'' S | 49° 27' 0.00'' W | MHNCI 414 |  |
| *D. albiventris* | 25° 28' 60.00'' S | 53° 37' 0.00'' W | MHNCI 2655 |  |
| *D. albiventris* | 24° 53' 36.89'' S | 49° 57' 57.14'' W | MHNCI 316 |  |
| *D. albiventris* | 25° 25' 2.50'' S | 49° 18' 16.73'' W |  | [21] |
| *D. albiventris* | 25° 25' 42.23'' S | 49° 16' 23.71'' W | MHNCI 30 |  |
| *D. albiventris* | 25° 19' 60.00'' S | 49° 10' 0.00'' W | MHNCI 2663 |  |
| *D. albiventris* | 25° 52' 60.00'' S | 48° 37' 60.00'' W | MHNCI 3878 |  |
| *D. albiventris* | 25° 22' 11.24'' S | 50° 33' 27.78'' W | MHNCI 1106 |  |
| *D. albiventris* | 23° 25' 44.26'' S | 51° 55' 48.15'' W |  | [22] |
| *D. albiventris* | 24° 13' 60.00'' S | 50° 55' 60.00'' W | MZUSP 31680 |  |
| *D. albiventris* | 24° 12' 24.19'' S | 50° 55' 27.94'' W | MZUSP 31627 |  |
| *D. albiventris* | 26° 28' 60.00'' S | 52° 0' 0.00'' W |  | [23] |
| *D. albiventris* | 25° 25' 60.00'' S | 50° 0' 0.00'' W | MHNCI 4249 |  |
| *D. albiventris* | 25° 26' 60.00'' S | 49° 12' 0.00'' W | MHNCI 3741 |  |
| *D. albiventris* | 23° 46' 47.25'' S | 50° 2' 7.93'' W | MZUSP 31379 |  |
| *D. albiventris* | 25° 55' 0.00'' S | 52° 10' 0.00'' W | MHNCI 1097 | [23] |
| *D. albiventris* | 25° 0' 0.00'' S | 50° 10' 0.00'' W | MHNCI 716 |  |
| *D. albiventris* | 25° 32' 49.08'' S | 49° 53' 29.81'' W | MHNCI 825 |  |
| *D. albiventris* | 25° 49' 60.00'' S | 52° 1' 60.00'' W | MHNCI 3969 |  |
| *D. albiventris* | 24° 13' 0.00'' S | 50° 32' 60.00'' W |  | [24] |
| *D. albiventris* | 25° 25' 0.00'' S | 53° 10' 0.00'' W |  | [25] |
| *D. albiventris* | 23° 26' 60.00'' S | 53° 22' 60.00'' W | MHNCI 354 |  |
| *D. albiventris* | 31° 19' 60.00'' S | 54° 0' 0.00'' W |  | [26] |
| *D. albiventris* | 30° 11' 8.63'' S | 57° 30' 5.15'' W | Personal information | Sponchiado J. |
| *D. albiventris* | 30° 0' 45.14'' S | 52° 55' 11.14'' W |  | [27] |
| *D. albiventris* | 29° 40' 0.00'' S | 52° 47' 60.00'' W | MCN 2964 |  |
| *D. albiventris* | 31° 23' 12.20'' S | 52° 40' 2.57'' W | Personal information | Cáceres NC (personal observation) |
| *D. albiventris* | 29° 56' 21.22'' S | 51° 8' 50.08'' W |  | [27] |
| *D. albiventris* | 29° 45' 18.81'' S | 50° 2' 30.94'' W | MCNU 533 | [28] |
| *D. albiventris* | 31° 46' 0.00'' S | 52° 34' 60.00'' W |  | [27] |
| *D. albiventris* | 31° 48' 48.80'' S | 52° 19' 3.98'' W |  | [27] |
| *D. albiventris* | 29° 48' 0.00'' S | 50° 17' 60.00'' W | MCNU 235 | [28] |
| *D. albiventris* | 31° 51' 25.38'' S | 52° 48' 51.45'' W | FZB 171 | [28] |
| *D. albiventris* | 28° 7' 0.00'' S | 55° 0' 0.00'' W |  |  |
| *D. albiventris* | 27° 11' 38.64'' S | 53° 53' 41.37'' W |  | [29] |
| *D. albiventris* | 30° 4' 34.00'' S | 55° 17' 3.10'' W | Personal information | Cáceres NC |
| *D. albiventris* | 29° 56' 0.80'' S | 54° 22' 5.80'' W | Personal information | Cáceres NC |
| *D. albiventris* | 27° 22' 29.36'' S | 53° 25' 41.91'' W | Personal information | Sponchiado J. |
| *D. albiventris* | 28° 11' 2.72'' S | 55° 38' 18.97'' W | FZB 336 |  |
| *D. albiventris* | 29° 56' 44.66'' S | 50° 59' 15.12'' W |  | [27] |
| *D. albiventris* | 29° 59' 45.25'' S | 51° 12' 59.99'' W | FZB 455 |  |
| *D. albiventris* | 29° 41' 21.57'' S | 51° 28' 0.65'' W | FZB 451 |  |
| *D. albiventris* | 29° 40' 49.08'' S | 50° 14' 18.27'' W |  | [27] |
| *D. albiventris* | 29° 30' 13.87'' S | 49° 59' 37.63'' W |  | [30] |
| *D. albiventris* | 30° 15' 0.00'' S | 50° 30' 0.00'' W | MCN 170 |  |
| *D. albiventris* | 28° 17' 24.63'' S | 53° 29' 27.26'' W | FZB 506 |  |
| *D. albiventris* | 30° 11' 24.75'' S | 52° 22' 24.65'' W | FZB 505 |  |
| *D. albiventris* | 31° 19' 60.00'' S | 54° 0' 0.00'' W | AMNH 238006 | [6] |
| *D. albiventris* | 30° 4' 10.38'' S | 51° 7' 14.36'' W | FZB 338 |  |
| *D. albiventris* | 30° 19' 60.00'' S | 50° 16' 0.00'' W | MCN 137 |  |
| *D. albiventris* | 32° 38' 14.01'' S | 52° 32' 23.70'' W |  | [31] |
| *D. albiventris* | 29° 42' 4.66'' S | 53° 50' 50.44'' W | FZB 337 |  |
| *D. albiventris* | 29° 40' 0.00'' S | 53° 43' 0.00'' W |  |  |
| *D. albiventris* | 29° 11' 44.00'' S | 54° 51' 1.00'' W |  | [27] |
| *D. albiventris* | 28° 10' 60.00'' S | 55° 37' 60.00'' W | MCN 336 |  |
| *D. albiventris* | 29° 40' 53.89'' S | 51° 27' 19.24'' W |  | [6] |
| *D. albiventris* | 29° 46' 0.00'' S | 51° 8' 60.00'' W | MCN 378 |  |
| *D. albiventris* | 31° 25' 0.00'' S | 52° 0' 0.00'' W | MCN 411 |  |
| *D. albiventris* | 31° 21' 46.52'' S | 51° 58' 43.87'' W | MZUSP 1650 |  |
| *D. albiventris* | 29° 50' 31.34'' S | 51° 8' 46.38'' W | FZB 018 | [28] |
| *D. albiventris* | 30° 36' 49.50'' S | 51° 40' 50.34'' W |  | [27] |
| *D. albiventris* | 30° 16' 15.90'' S | 53° 32' 31.90'' W | Personal information | Cáceres NC |
| *D. albiventris* | 32° 4' 37.60'' S | 52° 16' 19.80'' W | Personal information | Cáceres NC |
| *D. albiventris* | 29° 21' 0.00'' S | 49° 45' 0.00'' W |  | [32] |
| *D. albiventris* | 29° 58' 0.00'' S | 50° 6' 0.00'' W | MCN 64 |  |
| *D. albiventris* | 29° 55' 0.00'' S | 51° 45' 0.00'' W | FZB 389 |  |
| *D. albiventris* | 29° 52' 12.16'' S | 51° 22' 4.78'' W |  | [27] |
| *D. albiventris* | 29° 35' 44.40'' S | 53° 45' 58.80'' W | Personal information | Cáceres NC |
| *D. albiventris* | 27° 23' 11.00'' S | 53° 28' 39.30'' W | Personal information | Cáceres NC |
| *D. albiventris* | 29° 35' 31.81'' S | 52° 12' 21.24'' W |  | [33] |
| *D. albiventris* | 30° 17' 5.34'' S | 51° 1' 9.06'' W |  | [27] |
| *D. albiventris* | 30° 4' 60.00'' S | 51° 0' 0.00'' W | FZB 3012 |  |
| *D. albiventris* | 29° 30' 0.00'' S | 53° 25' 60.00'' W |  |  |
| *D. albiventris* | 27° 15' 0.00'' S | 49° 42' 0.00'' W | JC 2206 |  |
| *D. albiventris* | 27° 41' 60.00'' S | 49° 21' 0.00'' W | JC 2169 |  |
| *D. albiventris* | 27° 40' 60.00'' S | 51° 7' 60.00'' W |  | [34] |
| *D. albiventris* | 28° 52' 0.00'' S | 49° 31' 60.00'' W |  | [35] |
| *D. albiventris* | 26° 55' 0.00'' S | 49° 4' 0.00'' W |  | [34] |
| *D. albiventris* | 27° 43' 60.00'' S | 49° 47' 60.00'' W | JC 2009 |  |
| *D. albiventris* | 28° 43' 0.00'' S | 50° 28' 0.00'' W | JC 2066 |  |
| *D. albiventris* | 27° 48' 0.00'' S | 49° 28' 60.00'' W | JC 1988 |  |
| *D. albiventris* | 27° 19' 60.00'' S | 50° 58' 0.00'' W | JC 2053 |  |
| *D. albiventris* | 27° 4' 0.00'' S | 51° 38' 60.00'' W | JC 2145 |  |
| *D. albiventris* | 27° 13' 60.00'' S | 52° 1' 60.00'' W | MPEG 22223 | [6] |
| *D. albiventris* | 26° 43' 0.00'' S | 49° 28' 60.00'' W |  | [34] |
| *D. albiventris* | 26° 52' 0.00'' S | 52° 16' 0.00'' W | JC 2023 |  |
| *D. albiventris* | 27° 49' 30.48'' S | 48° 33' 23.36'' W |  | [36] |
| *D. albiventris* | 26° 1' 46.92'' S | 48° 51' 19.73'' W | MZUSP 7127 |  |
| *D. albiventris* | 28° 43' 0.00'' S | 49° 17' 60.00'' W |  | [34] |
| *D. albiventris* | 26° 53' 60.00'' S | 48° 49' 60.00'' W | UFSC 944 | [37] |
| *D. albiventris* | 26° 58' 60.00'' S | 53° 31' 60.00'' W |  | [34] |
| *D. albiventris* | 26° 37' 60.00'' S | 52° 27' 0.00'' W | UFSC 2808 |  |
| *D. albiventris* | 28° 37' 0.00'' S | 49° 1' 60.00'' W |  | [34] |
| *D. albiventris* | 27° 49' 0.00'' S | 50° 19' 60.00'' W |  | [34] |
| *D. albiventris* | 28° 28' 60.00'' S | 48° 46' 60.00'' W |  | [34] |
| *D. albiventris* | 28° 51' 0.00'' S | 49° 46' 60.00'' W |  | [34] |
| *D. albiventris* | 27° 55' 60.00'' S | 50° 6' 0.00'' W | UFSC 784 | [37] |
| *D. albiventris* | 27° 58' 0.00'' S | 48° 40' 60.00'' W |  | [34] |
| *D. albiventris* | 27° 25' 0.00'' S | 51° 45' 0.00'' W | JC 2171 |  |
| *D. albiventris* | 27° 22' 0.00'' S | 50° 25' 60.00'' W | JC 1681 |  |
| *D. albiventris* | 26° 52' 60.00'' S | 51° 58' 0.00'' W | JC 2002 |  |
| *D. albiventris* | 27° 16' 0.00'' S | 49° 55' 0.00'' W | JC 2027 |  |
| *D. albiventris* | 27° 40' 0.00'' S | 49° 1' 0.00'' W |  | [34] |
| *D. albiventris* | 27° 13' 60.00'' S | 49° 40' 0.00'' W | JC 2028 |  |
| *D. albiventris* | 27° 16' 60.00'' S | 50° 12' 0.00'' W | JC 2205 |  |
| *D. albiventris* | 28° 16' 60.00'' S | 49° 55' 60.00'' W | JC 2136 |  |
| *D. albiventris* | 27° 37' 0.00'' S | 48° 37' 60.00'' W | UFSC 797-798 | [37] |
| *D. albiventris* | 27° 8' 60.00'' S | 52° 19' 0.00'' W | JC 2097 |  |
| *D. albiventris* | 28° 36' 0.00'' S | 49° 32' 60.00'' W | UFSC 3074 |  |
| *D. albiventris* | 29° 7' 0.00'' S | 49° 37' 0.00'' W |  | [34] |
| *D. albiventris* | 26° 6' 0.00'' S | 50° 19' 0.00'' W |  | [38] |
| *D. albiventris* | 28° 28' 0.00'' S | 49° 0' 0.00'' W | UFSC 3143 |  |
| *D. albiventris* | 26° 51' 49.00'' S | 52° 9' 18.00'' W |  | [37] |
| *D. albiventris* | 26° 52' 0.00'' S | 52° 8' 60.00'' W |  | [34] |
| *D. albiventris* | 27° 0' 0.00'' S | 51° 43' 60.00'' W | JC 2146 |  |
| *D. albiventris* | 26° 52' 0.00'' S | 52° 19' 0.00'' W | JC 2041 |  |
| *D. albiventris* | 10° 26' 42.98'' S | 36° 28' 11.99'' W |  | [13] |
| *D. albiventris* | 22° 52' 52.33'' S | 49° 14' 20.62'' W | MZUSP 32195 |  |
| *D. albiventris* | 22° 53' 25.14'' S | 48° 27' 18.59'' W | MN 72147 |  |
| *D. albiventris* | 23° 48' 0.98'' S | 48° 35' 15.13'' W | MZUSP 31038 |  |
| *D. albiventris* | 22° 54' 25.57'' S | 47° 3' 47.66'' W | MN 25646 |  |
| *D. albiventris* | 21° 8' 56.79'' S | 51° 37' 51.17'' W |  | [9] |
| *D. albiventris* | 23° 33' 20.51'' S | 46° 9' 17.24'' W | MZUSP 22438 |  |
| *D. albiventris* | 23° 0' 51.43'' S | 48° 0' 22.72'' W | MZUSP 13736 |  |
| *D. albiventris* | 23° 0' 51.43'' S | 48° 0' 22.72'' W | MZUSP 13742 |  |
| *D. albiventris* | 22° 24' 2.70'' S | 49° 41' 59.27'' W |  | [39] |
| *D. albiventris* | 21° 15' 52.52'' S | 48° 41' 13.07'' W | MZUSP 9705 |  |
| *D. albiventris* | 23° 34' 25.15'' S | 48° 1' 20.01'' W | MZUSP 7130 |  |
| *D. albiventris* | 23° 42' 59.28'' S | 49° 29' 27.39'' W | MZUSP 17376 |  |
| *D. albiventris* | 20° 20' 6.91'' S | 47° 47' 47.79'' W | MZUSP 2993 |  |
| *D. albiventris* | 21° 40' 45.21'' S | 49° 44' 34.95'' W | MZUSP 6167 | [6] |
| *D. albiventris* | 22° 46' 54.19'' S | 47° 49' 4.73'' W |  | [39] |
| *D. albiventris* | 22° 49' 50.64'' S | 46° 55' 23.70'' W |  | [39] |
| *D. albiventris* | 22° 40' 37.60'' S | 48° 8' 21.49'' W |  | [39] |
| *D. albiventris* | 22° 25' 8.14'' S | 47° 28' 29.27'' W |  | [39] |
| *D. albiventris* | 22° 22' 15.05'' S | 46° 56' 16.23'' W | UNICAMP 1213 | [28] |
| *D. albiventris* | 22° 58' 59.28'' S | 49° 51' 25.57'' W | MN 23735 |  |
| *D. albiventris* | 22° 26' 57.84'' S | 52° 17' 35.01'' W |  | [39] |
| *D. albiventris* | 22° 43' 21.78'' S | 46° 55' 34.06'' W |  | [12] |
| *D. albiventris* | 22° 42' 29.40'' S | 47° 38' 30.96'' W |  | [40] |
| *D. albiventris* | 23° 41' 54.41'' S | 47° 37' 48.30'' W | MZUSP 16527 |  |
| *D. albiventris* | 21° 45' 50.26'' S | 52° 6' 22.05'' W | MZUSP 3716 |  |
| *D. albiventris* | 21° 10' 36.02'' S | 47° 49' 14.75'' W | MZUSP 7138 |  |
| *D. albiventris* | 22° 24' 50.48'' S | 47° 33' 46.02'' W | MN 50677 |  |
| *D. albiventris* | 24° 20' 23.20'' S | 48° 30' 55.29'' W | MN 50678 |  |
| *D. albiventris* | 10° 27' 53.30'' S | 50° 29' 35.05'' W |  | [41] |
| *D. albiventris* | 10° 40' 13.72'' S | 46° 52' 5.77'' W |  | [5] |
| *D. albiventris* | 10° 42' 25.90'' S | 48° 24' 51.47'' W | FLS 82 | [11] |
| *D. albiventris* | 11° 14' 52.97'' S | 46° 51' 49.50'' W |  | [5] |
| *D. albiventris* | 35° 17' 60.00'' S | 61° 25' 0.00'' W |  | [42] |
| *D. albiventris* | 34° 47' 60.00'' S | 58° 23' 60.00'' W | MACN 17257 | [43] |
| *D. albiventris* | 34° 13' 60.00'' S | 58° 52' 60.00'' W | MACN 19 | [43] |
| *D. albiventris* | 34° 22' 60.00'' S | 59° 49' 0.00'' W | CML 5481 | [43] |
| *D. albiventris* | 34° 49' 0.00'' S | 57° 58' 60.00'' W |  | [43] |
| *D. albiventris* | 38° 17' 60.00'' S | 58° 0' 0.00'' W |  | [43] |
| *D. albiventris* | 38° 16' 0.00'' S | 57° 51' 0.00'' W |  | [43] |
| *D. albiventris* | 38° 16' 0.00'' S | 57° 51' 0.00'' W | MACN 36757 | [43] |
| *D. albiventris* | 36° 57' 0.00'' S | 57° 4' 60.00'' W |  | [43] |
| *D. albiventris* | 36° 31' 0.00'' S | 56° 52' 60.00'' W | MACN 30.27 | [43] |
| *D. albiventris* | 38° 13' 60.00'' S | 57° 47' 60.00'' W |  | [43] |
| *D. albiventris* | 38° 15' 0.00'' S | 57° 47' 60.00'' W | BMNH 6.5.5.6 | [43] |
| *D. albiventris* | 37° 57' 0.00'' S | 57° 43' 60.00'' W | USNM 536826 | [43] |
| *D. albiventris* | 38° 0' 0.00'' S | 57° 32' 60.00'' W |  | [43] |
| *D. albiventris* | 34° 34' 60.00'' S | 61° 10' 0.00'' W |  | [43] |
| *D. albiventris* | 34° 55' 0.00'' S | 59° 15' 0.00'' W |  | [43] |
| *D. albiventris* | 34° 55' 0.00'' S | 57° 57' 0.00'' W |  | [43] |
| *D. albiventris* | 35° 15' 0.00'' S | 57° 46' 0.00'' W |  | [43] |
| *D. albiventris* | 35° 16' 0.00'' S | 57° 13' 60.00'' W | CML 3172 | [43] |
| *D. albiventris* | 35° 47' 60.00'' S | 61° 53' 60.00'' W |  | [42] |
| *D. albiventris* | 34° 10' 0.00'' S | 60° 45' 0.00'' W |  | [43] |
| *D. albiventris* | 35° 4' 60.00'' S | 58° 30' 0.00'' W | ARG 4511 | [43] |
| *D. albiventris* | 34° 25' 0.00'' S | 58° 34' 0.00'' W | CML 651 | [43] |
| *D. albiventris* | 38° 40' 0.00'' S | 63° 6' 0.00'' W |  | [43] |
| *D. albiventris* | 36° 45' 0.00'' S | 59° 51' 0.00'' W | MACN 35.13 | [43] |
| *D. albiventris* | 27° 4' 0.00'' S | 58° 42' 0.00'' W | MACN 17259 | [43] |
| *D. albiventris* | 27° 0' 0.00'' S | 58° 34' 0.00'' W | IADIZA | [43] |
| *D. albiventris* | 26° 30' 0.00'' S | 59° 34' 60.00'' W | BMNH 0.7.9.19 | [43] |
| *D. albiventris* | 26° 40' 0.00'' S | 59° 47' 60.00'' W | CML 506 4652 | [43] |
| *D. albiventris* | 30° 51' 0.00'' S | 65° 1' 0.00'' W | CML 440 | [43] |
| *D. albiventris* | 33° 21' 0.00'' S | 63° 17' 60.00'' W | MACN 425 | [43] |
| *D. albiventris* | 32° 22' 0.00'' S | 62° 19' 0.00'' W |  | [43] |
| *D. albiventris* | 30° 13' 0.00'' S | 65° 2' 60.00'' W | USNM 539387 | [43] |
| *D. albiventris* | 30° 22' 0.00'' S | 63° 55' 60.00'' W |  | [42] |
| *D. albiventris* | 32° 32' 60.00'' S | 62° 58' 60.00'' W | MACN 37.90 | [43] |
| *D. albiventris* | 32° 37' 60.00'' S | 62° 40' 0.00'' W | MACN 29799 | [43] |
| *D. albiventris* | 27° 28' 0.00'' S | 58° 49' 60.00'' W |  | [43] |
| *D. albiventris* | 29° 7' 60.00'' S | 59° 16' 0.00'' W |  | [43] |
| *D. albiventris* | 28° 1' 0.00'' S | 58° 1' 0.00'' W | MLP 5.V.99.6 | [43] |
| *D. albiventris* | 28° 40' 0.00'' S | 56° 10' 60.00'' W |  | [43] |
| *D. albiventris* | 28° 49' 60.00'' S | 65° 30' 0.00'' W | MACN 48125 | [43] |
| *D. albiventris* | 27° 31' 60.00'' S | 66° 24' 0.00'' W | MACN 34561 | [43] |
| *D. albiventris* | 27° 25' 0.00'' S | 66° 0' 0.00'' W | CML 3199 | [43] |
| *D. albiventris* | 35° 2' 60.00'' S | 58° 46' 0.00'' W | MACN 506 | [43] |
| *D. albiventris* | 28° 10' 0.00'' S | 65° 40' 60.00'' W | MACN 39203 | [43] |
| *D. albiventris* | 32° 7' 0.00'' S | 58° 19' 60.00'' W |  | [43] |
| *D. albiventris* | 31° 49' 0.00'' S | 58° 15' 0.00'' W | CML 1639 | [43] |
| *D. albiventris* | 31° 38' 60.00'' S | 58° 1' 0.00'' W | MMD 340 | [43] |
| *D. albiventris* | 32° 7' 0.00'' S | 60° 37' 60.00'' W |  | [42] |
| *D. albiventris* | 33° 1' 0.00'' S | 58° 31' 0.00'' W |  | [43] |
| *D. albiventris* | 24° 10' 0.00'' S | 61° 43' 60.00'' W | IADIZA 3418 | [43] |
| *D. albiventris* | 25° 1' 0.00'' S | 59° 19' 0.00'' W |  | [43] |
| *D. albiventris* | 24° 41' 60.00'' S | 60° 36' 0.00'' W |  | [43] |
| *D. albiventris* | 25° 19' 60.00'' S | 59° 43' 60.00'' W |  | [43] |
| *D. albiventris* | 25° 7' 60.00'' S | 58° 15' 0.00'' W | MACN 15432 | [43] |
| *D. albiventris* | 25° 10' 0.00'' S | 58° 10' 0.00'' W | MLP 26.V.99.1 | [43] |
| *D. albiventris* | 26° 25' 0.00'' S | 59° 28' 0.00'' W |  | [43] |
| *D. albiventris* | 24° 11' 60.00'' S | 65° 19' 0.00'' W | BMNH 9.12.1.55 | [43] |
| *D. albiventris* | 24° 1' 60.00'' S | 65° 7' 0.00'' W |  | [43] |
| *D. albiventris* | 23° 37' 0.00'' S | 64° 47' 60.00'' W |  | [43] |
| *D. albiventris* | 23° 46' 60.00'' S | 64° 46' 60.00'' W | MACN 31227 | [43] |
| *D. albiventris* | 23° 43' 60.00'' S | 64° 35' 60.00'' W | MACN 549 | [43] |
| *D. albiventris* | 23° 37' 60.00'' S | 64° 28' 0.00'' W |  | [43] |
| *D. albiventris* | 24° 13' 60.00'' S | 65° 13' 60.00'' W | MMP 88003 | [43] |
| *D. albiventris* | 24° 16' 0.00'' S | 65° 10' 0.00'' W |  | [43] |
| *D. albiventris* | 24° 11' 60.00'' S | 64° 50' 60.00'' W |  | [43] |
| *D. albiventris* | 24° 19' 0.00'' S | 64° 40' 60.00'' W | MACN 36192 | [43] |
| *D. albiventris* | 23° 43' 0.00'' S | 64° 31' 60.00'' W | MACN 36489 | [43] |
| *D. albiventris* | 23° 37' 0.00'' S | 65° 28' 0.00'' W | MACN 36753 | [43] |
| *D. albiventris* | 23° 34' 60.00'' S | 64° 54' 0.00'' W | BMNH 98.8.19.5 | [43] |
| *D. albiventris* | 36° 37' 0.00'' S | 64° 16' 60.00'' W |  | [42] |
| *D. albiventris* | 29° 18' 0.00'' S | 68° 12' 0.00'' W |  | [43] |
| *D. albiventris* | 29° 0' 0.00'' S | 68° 16' 60.00'' W | MACN 27.11 | [43] |
| *D. albiventris* | 27° 22' 41.78'' S | 54° 44' 38.00'' W |  | [42] |
| *D. albiventris* | 27° 6' 0.00'' S | 54° 57' 0.00'' W |  | [43] |
| *D. albiventris* | 27° 8' 44.37'' S | 54° 52' 23.24'' W |  | [43] |
| *D. albiventris* | 28° 30' 0.00'' S | 55° 43' 0.00'' W | MACN 51.56 | [43] |
| *D. albiventris* | 27° 19' 0.00'' S | 55° 31' 60.00'' W |  | [43] |
| *D. albiventris* | 27° 28' 24.94'' S | 55° 53' 29.65'' W |  | [43] |
| *D. albiventris* | 26° 23' 59.71'' S | 54° 37' 36.52'' W | MACN 17265 | [43] |
| *D. albiventris* | 25° 46' 60.00'' S | 54° 1' 60.00'' W | MLP 18.XI.41.9 | [43] |
| *D. albiventris* | 26° 4' 35.15'' S | 53° 59' 51.64'' W |  | [43] |
| *D. albiventris* | 27° 31' 26.30'' S | 55° 9' 36.49'' W | MLP 8.V.59.3 | [43] |
| *D. albiventris* | 25° 36' 0.00'' S | 54° 34' 60.00'' W |  | [43] |
| *D. albiventris* | 25° 37' 60.00'' S | 54° 30' 0.00'' W | MACN 17807 | [43] |
| *D. albiventris* | 26° 26' 60.00'' S | 54° 28' 0.00'' W |  | [43] |
| *D. albiventris* | 25° 38' 60.00'' S | 54° 19' 60.00'' W | MLP 16-VIII-40.1 | [43] |
| *D. albiventris* | 27° 36' 15.97'' S | 55° 19' 25.85'' W |  | [43] |
| *D. albiventris* | 27° 4' 60.00'' S | 55° 2' 60.00'' W | MACN 16546 | [43] |
| *D. albiventris* | 26° 34' 0.80'' S | 54° 45' 58.00'' W |  | [43] |
| *D. albiventris* | 27° 15' 31.18'' S | 55° 32' 21.28'' W | CML 437 | [43] |
| *D. albiventris* | 26° 37' 51.33'' S | 54° 6' 47.92'' W | CML 4067 | [43] |
| *D. albiventris* | 32° 52' 60.00'' S | 68° 49' 0.00'' W | CEM | [43] |
| *D. albiventris* | 33° 28' 0.00'' S | 67° 32' 60.00'' W | MACN 30.348 | [43] |
| *D. albiventris* | 33° 10' 0.00'' S | 68° 55' 0.00'' W | AMNH 185206 | [43] |
| *D. albiventris* | 33° 10' 60.00'' S | 68° 28' 0.00'' W | CML 20 | [43] |
| *D. albiventris* | 33° 46' 0.00'' S | 69° 1' 60.00'' W | MLP 3.VIII.99.14 | [43] |
| *D. albiventris* | 33° 4' 0.00'' S | 68° 28' 0.00'' W |  | [43] |
| *D. albiventris* | 34° 2' 60.00'' S | 67° 57' 0.00'' W | MACN 43.12 | [43] |
| *D. albiventris* | 33° 34' 60.00'' S | 68° 57' 0.00'' W | MLP 1692 | [43] |
| *D. albiventris* | 24° 41' 60.00'' S | 64° 37' 60.00'' W | BMNH 17.1.25.61 | [43] |
| *D. albiventris* | 25° 16' 60.00'' S | 64° 4' 0.00'' W |  | [43] |
| *D. albiventris* | 26° 4' 60.00'' S | 65° 58' 0.00'' W | MACN 36679 | [43] |
| *D. albiventris* | 26° 3' 0.00'' S | 65° 30' 0.00'' W |  | [42] |
| *D. albiventris* | 26° 7' 0.00'' S | 65° 16' 60.00'' W | CML 30222 | [43] |
| *D. albiventris* | 25° 4' 60.00'' S | 65° 10' 0.00'' W |  | [43] |
| *D. albiventris* | 22° 16' 0.00'' S | 63° 43' 60.00'' W | MACN 17263 | [43] |
| *D. albiventris* | 23° 15' 0.00'' S | 63° 21' 0.00'' W |  | [43] |
| *D. albiventris* | 23° 16' 34.03'' S | 63° 16' 58.47'' W | MMD 404 405 | [43] |
| *D. albiventris* | 25° 11' 60.00'' S | 64° 55' 0.00'' W |  | [43] |
| *D. albiventris* | 23° 15' 0.00'' S | 64° 10' 0.00'' W | MACN 29848 | [43] |
| *D. albiventris* | 22° 16' 60.00'' S | 62° 43' 0.00'' W | MACN 26.82 | [43] |
| *D. albiventris* | 24° 53' 60.00'' S | 65° 37' 60.00'' W | PIDBA 535 | [43] |
| *D. albiventris* | 22° 31' 0.00'' S | 64° 34' 0.00'' W |  | [43] |
| *D. albiventris* | 28° 7' 0.00'' S | 64° 13' 0.00'' W |  | [43] |
| *D. albiventris* | 26° 43' 0.00'' S | 64° 49' 60.00'' W |  | [43] |
| *D. albiventris* | 27° 28' 60.00'' S | 64° 52' 0.00'' W | CML 5482 | [43] |
| *D. albiventris* | 32° 28' 0.00'' S | 61° 34' 0.00'' W |  | [43] |
| *D. albiventris* | 31° 45' 0.00'' S | 61° 49' 60.00'' W |  | [43] |
| *D. albiventris* | 31° 11' 60.00'' S | 60° 10' 0.00'' W | MLP 17.II.71.1 | [43] |
| *D. albiventris* | 29° 22' 0.00'' S | 59° 49' 60.00'' W | MLP 31-VIII-41.1 | [43] |
| *D. albiventris* | 28° 28' 60.00'' S | 59° 37' 0.00'' W |  | [43] |
| *D. albiventris* | 28° 1' 60.00'' S | 59° 15' 0.00'' W | MACN 17262 | [43] |
| *D. albiventris* | 31° 28' 0.00'' S | 60° 55' 0.00'' W |  | [43] |
| *D. albiventris* | 32° 57' 0.00'' S | 60° 40' 0.00'' W | CML 1559 | [43] |
| *D. albiventris* | 32° 28' 0.00'' S | 60° 52' 60.00'' W |  | [42] |
| *D. albiventris* | 32° 32' 60.00'' S | 61° 23' 60.00'' W |  | [43] |
| *D. albiventris* | 32° 36' 0.00'' S | 66° 7' 60.00'' W | MACN 19193 19194 | [43] |
| *D. albiventris* | 33° 40' 0.00'' S | 65° 28' 0.00'' W | MLP 12.VIII.40.4 | [43] |
| *D. albiventris* | 32° 21' 0.00'' S | 64° 58' 60.00'' W | MACN 34.586 | [43] |
| *D. albiventris* | 24° 34' 0.00'' S | 65° 49' 60.00'' W |  | [42] |
| *D. albiventris* | 26° 37' 0.00'' S | 65° 12' 0.00'' W | MLP 27.X.97.05 | [43] |
| *D. albiventris* | 26° 34' 60.00'' S | 64° 57' 0.00'' W |  | [43] |
| *D. albiventris* | 26° 30' 0.00'' S | 64° 52' 0.00'' W |  | [43] |
| *D. albiventris* | 26° 34' 60.00'' S | 64° 57' 0.00'' W |  | [43] |
| *D. albiventris* | 26° 49' 60.00'' S | 65° 13' 0.00'' W |  | [43] |
| *D. albiventris* | 26° 49' 0.00'' S | 65° 13' 0.00'' W | MLP 28.IV.50.3 | [43] |
| *D. albiventris* | 26° 49' 0.00'' S | 65° 10' 60.00'' W | PIDBA 837 | [43] |
| *D. albiventris* | 27° 19' 60.00'' S | 63° 34' 60.00'' W | MACN 14 | [43] |
| *D. albiventris* | 26° 55' 0.00'' S | 65° 10' 0.00'' W | MACN 27.98 | [43] |
| *D. albiventris* | 26° 49' 60.00'' S | 65° 10' 0.00'' W | CML 3174 | [43] |
| *D. albiventris* | 27° 49' 0.00'' S | 65° 19' 60.00'' W | 30100 | [43] |
| *D. albiventris* | 27° 40' 0.00'' S | 65° 46' 0.00'' W | ARG 4506 | [43] |
| *D. albiventris* | 27° 8' 60.00'' S | 65° 15' 0.00'' W |  | [43] |
| *D. albiventris* | 27° 23' 60.00'' S | 65° 7' 60.00'' W | MACN 39198 | [43] |
| *D. albiventris* | 27° 10' 0.00'' S | 65° 30' 0.00'' W |  | [43] |
| *D. albiventris* | 34° 25' 60.00'' S | 58° 34' 0.00'' W | MACN 39719 | [43] |
| *D. albiventris* | 26° 52' 0.00'' S | 65° 40' 60.00'' W | MACN 13067 | [43] |
| *D. albiventris* | 26° 15' 0.00'' S | 65° 28' 60.00'' W |  | [43] |
| *D. albiventris* | 26° 23' 60.00'' S | 65° 19' 60.00'' W |  | [43] |
| *D. albiventris* | 26° 22' 60.00'' S | 65° 17' 60.00'' W |  | [43] |
| *D. albiventris* | 26° 37' 0.00'' S | 65° 12' 0.00'' W |  | [43] |
| *D. albiventris* | 26° 46' 60.00'' S | 65° 22' 60.00'' W | CML 5976 | [43] |
| *D. albiventris* | 26° 46' 60.00'' S | 65° 22' 60.00'' W |  | [43] |
| *D. albiventris* | 26° 45' 0.00'' S | 65° 20' 60.00'' W | CML 4832 | [43] |
| *D. albiventris* | 26° 45' 0.00'' S | 65° 20' 60.00'' W | PIDBA 645 | [43] |
| *D. albiventris* | 26° 45' 0.00'' S | 65° 20' 60.00'' W |  | [43] |
| *D. albiventris* | 26° 45' 0.00'' S | 65° 20' 60.00'' W |  | [43] |
| *D. albiventris* | 26° 37' 0.00'' S | 65° 12' 0.00'' W | USNM 331055 | [43] |
| *D. albiventris* | 16° 25' 0.11'' S | 68° 4' 0.01'' W | AMNH M-38715 | GBIF |
| *D. albiventris* | 17° 23' 29.70'' S | 66° 19' 1.39'' W | AMNH M-38774 | GBIF |
| *D. albiventris* | 17° 25' 60.00'' S | 66° 19' 0.00'' W |  | [43] |
| *D. albiventris* | 17° 31' 0.01'' S | 66° 7' 0.01'' W | MSB 70285 | MaNIS |
| *D. albiventris* | 17° 24' 36.00'' S | 65° 21' 36.00'' W | AMNH M-264925 | GBIF |
| *D. albiventris* | 20° 51' 0.00'' S | 63° 10' 0.01'' W | MSB 55832 | MaNIS |
| *D. albiventris* | 19° 3' 0.00'' S | 64° 49' 59.99'' W | MSB 63279 | MaNIS |
| *D. albiventris* | 20° 51' 0.00'' S | 63° 10' 1.20'' W | MSB 55832 | GBIF |
| *D. albiventris* | 19° 4' 0.00'' S | 64° 49' 0.00'' W |  | [43] |
| *D. albiventris* | 15° 36' 8.10'' S | 68° 20' 2.11'' W | AMNH M-72573 | GBIF |
| *D. albiventris* | 16° 7' 0.01'' S | 68° 4' 0.01'' W | MSB 70284 | MaNIS |
| *D. albiventris* | 19° 34' 58.88'' S | 65° 45' 21.83'' W | AMNH M-39005 | GBIF |
| *D. albiventris* | 16° 22' 32.25'' S | 60° 57' 16.99'' W | USNM 391488 | GBIF |
| *D. albiventris* | 17° 46' 45.66'' S | 63° 10' 49.14'' W | AMNH M-246454 | GBIF |
| *D. albiventris* | 16° 22' 60.00'' S | 60° 58' 60.00'' W |  | [43] |
| *D. albiventris* | 21° 25' 58.80'' S | 63° 55' 1.20'' W | MSB 239808 | GBIF |
| *D. albiventris* | 18° 45' 0.00'' S | 62° 13' 1.20'' W | RAP 18982 | GBIF |
| *D. albiventris* | 20° 26’ 60.00’’ S | 63° 7’ 48.00’’ W | AMNH M-261286 | GBIF |
| *D. albiventris* | 17° 31' 48.00'' S | 64° 20' 60.00'' W | AMNH M-260040 | GBIF |
| *D. albiventris* | 18° 45' 0.00'' S | 62° 13' 1.20'' W | RAP 18982 | GBIF |
| *D. albiventris* | 21° 28' 0.00'' S | 64° 31' 60.00'' W |  | [43] |
| *D. albiventris* | 21° 25' 60.00'' S | 63° 55' 0.00'' W | MSB 239808 | MaNIS |
| *D. albiventris* | 21° 16' 12.00'' S | 64° 11' 24.00'' W | MSB 236882 | MaNIS |
| *D. albiventris* | 21° 26' 60.00'' S | 64° 19' 1.20'' W | MSB 56999 | GBIF |
| *D. albiventris* | 21° 15' 36.00'' S | 63° 32' 60.00'' W | AMNH M-263967 | GBIF |
| *D. albiventris* | 19° 55' 1.20'' S | 62° 34' 1.20'' W | RAP 4982 | GBIF |
| *D. albiventris* | 21° 1' 58.80'' S | 57° 53' 60.00'' W | FMNH 145242 | GBIF |
| *D. albiventris* | 24° 48' 57.60'' S | 54° 28' 1.20'' W |  | [44] |
| *D. albiventris* | 22° 39' 14.05'' S | 56° 11' 32.22'' W | NMNH 536826 | [6] |
| *D. albiventris* | 22° 37' 1.20'' S | 55° 58' 58.80'' W | UMMZ 125450 | GBIF |
| *D. albiventris* | 22° 30' 0.00'' S | 60° 0' 0.00'' W | FMNH 63859 |  |
| *D. albiventris* | 22° 26' 60.00'' S | 62° 19' 58.80'' W | FMNH 54342 | GBIF |
| *D. albiventris* | 25° 28' 11.57'' S | 56° 0' 55.21'' W | MSB 208416 | GBIF |
| *D. albiventris* | 25° 12' 50.40'' S | 56° 27' 0.00'' W | UMMZ 124684 | GBIF |
| *D. albiventris* | 26° 27' 32.40'' S | 55° 45' 28.80'' W |  | [44] |
| *D. albiventris* | 24° 29' 16.11'' S | 55° 42' 29.84'' W | UMMZ 134041 | [45] |
| *D. albiventris* | 24° 9' 21.60'' S | 55° 42' 21.60'' W | UMMZ 174934 | GBIF |
| *D. albiventris* | 24° 26' 60.00'' S | 55° 38' 60.00'' W | UMMZ 134033 | GBIF |
| *D. albiventris* | 24° 8' 0.97'' S | 54° 30' 0.19'' W | MACN 13.139 | [6] |
| *D. albiventris* | 24° 32' 16.80'' S | 55° 21' 3.60'' W |  | [44] |
| *D. albiventris* | 24° 8' 56.40'' S | 55° 25' 55.20'' W |  | [44] |
| *D. albiventris* | 25° 16' 53.19'' S | 57° 38' 5.86'' W | MACN 31.239 | [6] |
| *D. albiventris* | 25° 16' 0.12'' S | 57° 40' 0.12'' W | MVZ 144317 | MaNIS |
| *D. albiventris* | 25° 16' 1.20'' S | 57° 40' 1.20'' W | UMMZ 134567 | GBIF |
| *D. albiventris* | 25° 16' 1.20'' S | 57° 40' 1.20'' W | MVZ 144316 | GBIF |
| *D. albiventris* | 23° 21' 42.12'' S | 57° 23' 9.60'' W | MSB 82529 | MaNIS |
| *D. albiventris* | 23° 21' 43.20'' S | 57° 23' 9.60'' W | MSB 82529 | GBIF |
| *D. albiventris* | 25° 16' 58.80'' S | 57° 6' 0.00'' W | UMMZ 126106 | GBIF |
| *D. albiventris* | 25° 16' 59.99'' S | 57° 6' 0.00'' W | UMMZ 134042 | MaNIS |
| *D. albiventris* | 25° 16' 58.80'' S | 57° 6' 0.00'' W | UMMZ 134042 | GBIF |
| *D. albiventris* | 25° 4' 12.00'' S | 57° 4' 1.20'' W | MVZ 144320 | GBIF |
| *D. albiventris* | 25° 47' 7.43'' S | 56° 27' 1.13'' W |  | [6] |
| *D. albiventris* | 26° 43' 15.60'' S | 55° 34' 8.40'' W | UMMZ 174847 | GBIF |
| *D. albiventris* | 26° 56' 0.82'' S | 55° 15' 4.95'' W | MACN 350-47 | GBIF |
| *D. albiventris* | 27° 7' 42.23'' S | 55° 42' 28.21'' W | AMNH M-36521 | GBIF |
| *D. albiventris* | 28° 37' 60.00'' S | 65° 22' 60.00'' W |  | [43] |
| *D. albiventris* | 27° 19' 58.80'' S | 56° 25' 8.40'' W | UMMZ 134046 | GBIF |
| *D. albiventris* | 26° 31' 12.00'' S | 55° 47' 60.00'' W | KUM 158895 | GBIF |
| *D. albiventris* | 27° 23' 11.07'' S | 56° 50' 52.72'' W | AMNH M-248304 | GBIF |
| *D. albiventris* | 27° 23' 10.12'' S | 56° 50' 52.07'' W | MACN 50.476 | [6] |
| *D. albiventris* | 26° 53' 12.02'' S | 57° 1' 42.01'' W | NMNH 391489 | GBIF |
| *D. albiventris* | 27° 23' 23.44'' S | 56° 50' 52.35'' W | AMNH 248304 | [6] |
| *D. albiventris* | 26° 24' 28.80'' S | 57° 2' 45.60'' W | UMMZ 174886 | GBIF |
| *D. albiventris* | 27° 20' 56.40'' S | 56° 48' 10.80'' W | UMMZ 125451 | GBIF |
| *D. albiventris* | 27° 9' 32.40'' S | 57° 38' 60.00'' W | UMMZ 124682 | GBIF |
| *D. albiventris* | 27° 19' 58.80'' S | 55° 55' 12.00'' W | UMMZ 125261 | GBIF |
| *D. albiventris* | 25° 43' 44.40'' S | 57° 14' 24.00'' W | UMMZ 134057 | GBIF |
| *D. albiventris* | 26° 5' 43.61'' S | 56° 50' 19.37'' W | BM 2.2.5.32 | [6] |
| *D. albiventris* | 25° 40' 3.96'' S | 56° 57' 20.02'' W | NRM 581176 | GBIF |
| *D. albiventris* | 26° 1' 1.20'' S | 57° 2' 60.00'' W | USNM 531153 | GBIF |
| *D. albiventris* | 25° 5' 15.68'' S | 57° 31' 46.66'' W | UMMZ 134058 | [45] |
| *D. albiventris* | 24° 57' 57.60'' S | 56° 31' 26.40'' W | UMMZ 134029 | GBIF |
| *D. albiventris* | 24° 5' 31.20'' S | 56° 25' 8.40'' W | UMMZ 174910 | GBIF |
| *D. albiventris* | 30° 44' 14.09'' S | 57° 47' 6.77'' W | AMNH | [27] |
| *D. albiventris* | 34° 49' 55.00'' S | 56° 2' 46.00'' W | FMNH | [27] |
| *D. albiventris* | 34° 47' 2.04'' S | 55° 24' 37.99'' W | MNHN | [27] |
| *D. albiventris* | 34° 45' 9.64'' S | 55° 50' 18.78'' W | MNHN | [27] |
| *D. albiventris* | 34° 42' 16.70'' S | 56° 13' 36.76'' W | Brown 2004 | [27] |
| *D. albiventris* | 34° 49' 4.36'' S | 55° 55' 47.20'' W | ZVC-M | [27] |
| *D. albiventris* | 34° 47' 19.32'' S | 55° 51' 22.39'' W | ZVC-M | [27] |
| *D. albiventris* | 34° 46' 45.82'' S | 55° 45' 34.63'' W | Montevideo 793 | [6] |
| *D. albiventris* | 34° 47' 5.69'' S | 55° 24' 9.64'' W | Montevideo 103 | [6] |
| *D. albiventris* | 32° 54' 5.58'' S | 55° 18' 30.42'' W | ZVC-M | [27] |
| *D. albiventris* | 32° 25' 3.60'' S | 54° 6' 42.09'' W | AMNH | [27] |
| *D. albiventris* | 32° 41' 57.43'' S | 53° 47' 43.10'' W | AMNH | [27] |
| *D. albiventris* | 34° 0' 31.18'' S | 58° 17' 47.12'' W | ZVC-M | [27] |
| *D. albiventris* | 34° 10' 19.69'' S | 58° 6' 16.19'' W | ZVC-M | [27] |
| *D. albiventris* | 33° 57' 59.40'' S | 57° 22' 13.31'' W | FMNH | [27] |
| *D. albiventris* | 32° 46' 53.01'' S | 55° 56' 16.55'' W | AMNH | [27] |
| *D. albiventris* | 33° 34' 51.09'' S | 56° 21' 43.58'' W | ZVC-M | [27] |
| *D. albiventris* | 34° 22' 30.50'' S | 55° 13' 36.53'' W | MNHN | [27] |
| *D. albiventris* | 33° 41' 49.25'' S | 54° 57' 5.60'' W | Palerm 1950 | [27] |
| *D. albiventris* | 34° 55' 4.96'' S | 54° 58' 29.89'' W | MNHN | [27] |
| *D. albiventris* | 34° 55' 4.96'' S | 54° 11' 9.29'' W | FMNH | [27] |
| *D. albiventris* | 34° 51' 24.99'' S | 55° 16' 18.31'' W | Brown 2004 | [27] |
| *D. albiventris* | 34° 53' 56.31'' S | 55° 15' 25.37'' W | ZVC-M | [27] |
| *D. albiventris* | 34° 47' 36.74'' S | 55° 23' 8.40'' W | ZVC-M | [27] |
| *D. albiventris* | 34° 45' 35.95'' S | 54° 44' 44.06'' W | ZVC-M | [27] |
| *D. albiventris* | 34° 47' 55.08'' S | 56° 4' 51.66'' W | ZVC-M | [27] |
| *D. albiventris* | 34° 51' 33.03'' S | 56° 12' 2.02'' W | ZVC-M | [27] |
| *D. albiventris* | 34° 53' 38.79'' S | 56° 15' 45.97'' W | ZVC-M | [27] |
| *D. albiventris* | 34° 49' 57.53'' S | 56° 23' 37.20'' W | ZVC-M | [27] |
| *D. albiventris* | 34° 47' 15.81'' S | 56° 20' 20.66'' W | Brown 2004 | [27] |
| *D. albiventris* | 34° 53' 38.79'' S | 56° 15' 45.97'' W | ZVC-M | [27] |
| *D. albiventris* | 34° 50' 12.57'' S | 56° 13' 16.21'' W | ZVC-M | [27] |
| *D. albiventris* | 32° 9' 31.00'' S | 57° 33' 16.00'' W | CECN 1993 | [27] |
| *D. albiventris* | 33° 6' 48.69'' S | 58° 11' 39.39'' W | MNHN | [27] |
| *D. albiventris* | 32° 28' 35.03'' S | 58° 8' 50.68'' W | AMNH | [27] |
| *D. albiventris* | 33° 6' 17.97'' S | 57° 59' 13.34'' W | ZVC-M | [27] |
| *D. albiventris* | 34° 6' 5.98'' S | 54° 10' 37.23'' W | Brown 2004 | [27] |
| *D. albiventris* | 34° 33' 9.06'' S | 54° 6' 29.75'' W | ZVC-M | [27] |
| *D. albiventris* | 34° 21' 29.63'' S | 53° 52' 4.01'' W | Gambarotta 1999 | [27] |
| *D. albiventris* | 33° 58' 9.92'' S | 53° 38' 15.81'' W | Maneyro 1995 | [27] |
| *D. albiventris* | 31° 15' 23.27'' S | 57° 46' 19.54'' W | MNHN | [27] |
| *D. albiventris* | 34° 20' 18.21'' S | 57° 0' 7.68'' W | ZVC-M | [27] |
| *D. albiventris* | 33° 30' 4.76'' S | 58° 25' 28.13'' W | ZVC-M | [27] |
| *D. albiventris* | 33° 36' 56.21'' S | 58° 21' 11.64'' W | FMNH | [27] |
| *D. albiventris* | 33° 39' 0.83'' S | 58° 4' 30.56'' W | ZVC-M | [27] |
| *D. albiventris* | 33° 52' 11.86'' S | 57° 20' 2.59'' W | AMNH | [27] |
| *D. albiventris* | 32° 25' 33.54'' S | 55° 25' 37.38'' W | ZVC-M | [27] |
| *D. albiventris* | 32° 55' 45.58'' S | 54° 27' 40.39'' W | AMNH | [27] |
| *D. aurita* | 9° 17' 6.62'' S | 36° 30' 49.44'' W | MZUSP 7366 |  |
| *D. aurita* | 9° 47' 48.68'' S | 36° 5' 54.05'' W |  | [13] |
| *D. aurita* | 14° 47' 50.13'' S | 39° 2' 3.82'' W | MZUSP 3494 |  |
| *D. aurita* | 14° 48' 15.16'' S | 39° 16' 27.96'' W |  | [13] |
| *D. aurita* | 12° 54' 15.16'' S | 38° 40' 27.96'' W |  | [13] |
| *D. aurita* | 15° 27' 53.03'' S | 39° 39' 0.27'' W | UFPB 597 | [11] |
| *D. aurita* | 15° 11' 43.44'' S | 39° 1' 47.53'' W |  | [46] |
| *D. aurita* | 17° 53' 38.02'' S | 39° 22' 32.53'' W |  | [13] |
| *D. aurita* | 20° 30' 48.16'' S | 41° 45' 52.79'' W |  | [12] |
| *D. aurita* | 20° 16' 52.00'' S | 40° 31' 19.00'' W |  | [47] |
| *D. aurita* | 18° 16' 0.00'' S | 39° 47' 60.00'' W |  | [48] |
| *D. aurita* | 18° 17' 56.25'' S | 39° 51' 30.24'' W |  | [48] |
| *D. aurita* | 19° 0' 43.88'' S | 40° 2' 26.73'' W |  | [48] |
| *D. aurita* | 18° 20' 20.00'' S | 40° 8' 51.00'' W |  | [48] |
| *D. aurita* | 19° 56' 9.14'' S | 40° 36' 11.15'' W |  | [49] |
| *D. aurita* | 19° 56' 17.78'' S | 40° 35' 45.36'' W | MZUSP 6204 |  |
| *D. aurita* | 18° 43' 15.33'' S | 39° 51' 49.01'' W | MN 51871 |  |
| *D. aurita* | 20° 7' 41.60'' S | 40° 18' 35.56'' W | MN 11671 |  |
| *D. aurita* | 20° 20' 58.00'' S | 40° 30' 20.00'' W |  | [47] |
| *D. aurita* | 20° 22' 44.00'' S | 40° 28' 31.00'' W |  | [47] |
| *D. aurita* | 20° 21' 33.00'' S | 40° 28' 22.00'' W |  | [47] |
| *D. aurita* | 20° 22' 45.00'' S | 40° 28' 6.00'' W |  | [47] |
| *D. aurita* | 20° 23' 12.00'' S | 40° 27' 25.00'' W |  | [47] |
| *D. aurita* | 20° 23' 12.00'' S | 40° 27' 24.00'' W |  | [47] |
| *D. aurita* | 20° 20' 33.38'' S | 40° 17' 34.02'' W | MN 3857 |  |
| *D. aurita* | 20° 17' 52.13'' S | 40° 20' 0.47'' W |  | [50] |
| *D. aurita* | 21° 51' 49.21'' S | 42° 40' 7.86'' W | MN 3850 |  |
| *D. aurita* | 19° 43' 35.81'' S | 41° 49' 18.67'' W |  | [51] |
| *D. aurita* | 18° 51' 17.72'' S | 41° 56' 56.40'' W |  | [13] |
| *D. aurita* | 16° 26' 15.39'' S | 41° 0' 8.56'' W |  | [13] |
| *D. aurita* | 19° 47' 46.81'' S | 42° 38' 0.79'' W |  | [51] |
| *D. aurita* | 20° 54' 50.47'' S | 44° 50' 23.68'' W |  | [15] |
| *D. aurita* | 17° 51' 53.01'' S | 41° 30' 52.52'' W | MZUSP 2732 |  |
| *D. aurita* | 19° 20' 25.76'' S | 42° 50' 4.99'' W |  | [13] |
| *D. aurita* | 19° 42' 37.14'' S | 42° 34' 25.13'' W | UFSC 2410 |  |
| *D. aurita* | 21° 34' 60.00'' S | 55° 12' 0.00'' W | Personal information | Sponchiado J |
| *D. aurita* | 20° 9' 29.04'' S | 42° 37' 9.50'' W |  | [51] |
| *D. aurita* | 20° 58' 60.00'' S | 54° 30' 0.00'' W | Personal information | Sponchiado J |
| *D. aurita* | 23° 13' 47.49'' S | 53° 43' 27.03'' W |  | [13] |
| *D. aurita* | 7° 7' 53.40'' S | 34° 52' 53.72'' W |  | [13] |
| *D. aurita* | 8° 42' 37.00'' S | 35° 50' 1.00'' W |  | [1] |
| *D. aurita* | 8° 3' 32.86'' S | 34° 54' 52.34'' W |  | [13] |
| *D. aurita* | 25° 25' 60.00'' S | 48° 42' 0.00'' W | MHNCI 172 |  |
| *D. aurita* | 25° 31' 55.91'' S | 49° 23' 39.94'' W | MHNCI 2654 |  |
| *D. aurita* | 26° 4' 60.00'' S | 51° 30' 0.00'' W |  | [23] |
| *D. aurita* | 25° 49' 44.03'' S | 48° 32' 28.02'' W | MHNCI 331 |  |
| *D. aurita* | 25° 22' 0.00'' S | 49° 27' 0.00'' W | MHNCI 409 |  |
| *D. aurita* | 25° 19' 19.87'' S | 49° 9' 28.53'' W | MHNCI 4326 |  |
| *D. aurita* | 25° 40' 40.51'' S | 49° 31' 10.80'' W | MHNCI 335 |  |
| *D. aurita* | 26° 13' 60.00'' S | 52° 0' 0.00'' W |  | [23] |
| *D. aurita* | 25° 25' 2.50'' S | 49° 18' 16.73'' W |  | [52] |
| *D. aurita* | 25° 25' 60.00'' S | 49° 18' 14.02'' W | MHNCI 3879 |  |
| *D. aurita* | 25° 25' 60.00'' S | 49° 18' 14.00'' W | MHNCI 3875 |  |
| *D. aurita* | 25° 25' 0.00'' S | 49° 17' 60.00'' W |  | [53] |
| *D. aurita* | 25° 25' 43.76'' S | 49° 16' 23.53'' W | MHNCI 129 |  |
| *D. aurita* | 25° 38' 60.00'' S | 49° 17' 60.00'' W | MHNCI 4785 |  |
| *D. aurita* | 25° 30' 0.00'' S | 54° 30' 0.00'' W | MHNCI 3819 |  |
| *D. aurita* | 25° 40' 49.45'' S | 54° 26' 2.31'' W | MHNCI 206 |  |
| *D. aurita* | 25° 18' 0.00'' S | 48° 22' 60.00'' W | MHNCI 2743 |  |
| *D. aurita* | 25° 53' 3.85'' S | 48° 34' 34.34'' W | MHNCI 246 |  |
| *D. aurita* | 25° 49' 0.00'' S | 48° 32' 60.00'' W | MHNCI 3965 |  |
| *D. aurita* | 25° 30' 0.00'' S | 48° 49' 60.00'' W | MHNCI 2068 |  |
| *D. aurita* | 26° 5' 59.17'' S | 49° 25' 36.38'' W | MHNCI 3881 |  |
| *D. aurita* | 23° 46' 47.45'' S | 50° 2' 7.97'' W | MZUSP 31381 |  |
| *D. aurita* | 25° 28' 0.00'' S | 48° 49' 60.00'' W |  | [54] |
| *D. aurita* | 25° 7' 13.42'' S | 50° 10' 19.19'' W | MHNCI 2662 |  |
| *D. aurita* | 23° 25' 0.00'' S | 53° 46' 0.00'' W | MZUSP 7714 |  |
| *D. aurita* | 25° 35' 9.84'' S | 48° 36' 59.72'' W | MHNCI 29 |  |
| *D. aurita* | 25° 26' 44.84'' S | 49° 30' 4.79'' W | MHNCI 6191 |  |
| *D. aurita* | 25° 43' 35.15'' S | 48° 56' 57.25'' W | MHNCI 1738 |  |
| *D. aurita* | 24° 13' 0.00'' S | 50° 32' 60.00'' W |  | [24] |
| *D. aurita* | 25° 55' 60.00'' S | 49° 12' 0.00'' W | MHNCI 1737 |  |
| *D. aurita* | 25° 40' 0.00'' S | 48° 57' 0.00'' W | MZUSP 27798 |  |
| *D. aurita* | 25° 25' 0.00'' S | 53° 10' 0.00'' W |  | [25] |
| *D. aurita* | 23° 26' 60.00'' S | 53° 22' 60.00'' W | MHNCI 356 |  |
| *D. aurita* | 23° 52' 60.00'' S | 49° 47' 60.00'' W | MZUSP 31903 |  |
| *D. aurita* | 24° 4' 60.00'' S | 54° 30' 0.00'' W | MHNCI 408 |  |
| *D. aurita* | 25° 49' 60.00'' S | 48° 49' 60.00'' W | MHNCI 210 |  |
| *D. aurita* | 25° 52' 60.00'' S | 48° 34' 0.00'' W | MHNCI 197 |  |
| *D. aurita* | 23° 0' 36.18'' S | 44° 19' 6.18'' W | MN 72113 |  |
| *D. aurita* | 22° 57' 27.26'' S | 42° 53' 44.29'' W |  | [55] |
| *D. aurita* | 22° 27' 49.99'' S | 42° 39' 9.85'' W |  | [56] |
| *D. aurita* | 22° 28' 47.12'' S | 42° 12' 10.32'' W | MN 50631 |  |
| *D. aurita* | 22° 47' 12.57'' S | 43° 18' 47.18'' W | MN 5864 |  |
| *D. aurita* | 22° 28' 29.00'' S | 42° 59' 32.00'' W |  | [57] |
| *D. aurita* | 22° 28' 27.06'' S | 42° 59' 6.08'' W |  | [57] |
| *D. aurita* | 22° 51' 58.15'' S | 43° 46' 37.21'' W | MN 72359 |  |
| *D. aurita* | 22° 22' 59.90'' S | 43° 7' 58.88'' W | MN 50648 |  |
| *D. aurita* | 22° 29' 14.25'' S | 44° 33' 30.89'' W | MN 41974 |  |
| *D. aurita* | 22° 21' 29.64'' S | 41° 46' 47.46'' W | MN 55058 |  |
| *D. aurita* | 22° 39' 16.78'' S | 43° 2' 23.31'' W | MN 42795 |  |
| *D. aurita* | 22° 57' 32.07'' S | 44° 2' 20.76'' W | MN 73789 |  |
| *D. aurita* | 22° 55' 8.57'' S | 42° 49' 7.38'' W | MN 25695 |  |
| *D. aurita* | 22° 31' 34.83'' S | 43° 43' 39.55'' W | MN 69794 |  |
| *D. aurita* | 22° 27' 19.48'' S | 43° 28' 21.00'' W | MN 25031 |  |
| *D. aurita* | 22° 52' 50.18'' S | 43° 6' 20.89'' W | MN 66234 |  |
| *D. aurita* | 22° 19' 2.72'' S | 42° 20' 1.34'' W | MZUSP 2808 |  |
| *D. aurita* | 23° 12' 57.10'' S | 44° 42' 42.07'' W | MN 62189 |  |
| *D. aurita* | 22° 30' 15.69'' S | 43° 10' 55.90'' W | MN 50672 |  |
| *D. aurita* | 22° 38' 0.55'' S | 43° 54' 16.57'' W | MN 75296 |  |
| *D. aurita* | 22° 43' 41.06'' S | 44° 7' 52.53'' W | MN 77812 |  |
| *D. aurita* | 22° 31' 38.19'' S | 41° 56' 44.26'' W | MN 25677 |  |
| *D. aurita* | 22° 56' 59.73'' S | 43° 12' 44.89'' W |  | [58] |
| *D. aurita* | 22° 30' 0.28'' S | 42° 30' 6.59'' W |  | [59] |
| *D. aurita* | 22° 32' 35.13'' S | 42° 19' 35.30'' W |  | [60] |
| *D. aurita* | 21° 28' 25.26'' S | 41° 6' 36.44'' W | MN 69883 |  |
| *D. aurita* | 22° 50' 49.52'' S | 44° 3' 1.97'' W | MN 5754 |  |
| *D. aurita* | 22° 39' 33.56'' S | 42° 22' 53.46'' W | MN 29413 |  |
| *D. aurita* | 22° 2' 44.29'' S | 42° 41' 20.20'' W |  | [61] |
| *D. aurita* | 22° 22' 3.43'' S | 42° 45' 8.39'' W |  | [56] |
| *D. aurita* | 29° 25' 0.00'' S | 51° 30' 0.00'' W | PUCRS 592 |  |
| *D. aurita* | 30° 0' 45.14'' S | 52° 55' 11.14'' W |  | [27] |
| *D. aurita* | 27° 11' 38.64'' S | 53° 53' 41.37'' W |  | [62] |
| *D. aurita* | 29° 41' 18.18'' S | 51° 28' 1.36'' W |  | [27] |
| *D. aurita* | 29° 30' 13.87'' S | 49° 59' 37.63'' W |  | [30] |
| *D. aurita* | 30° 3' 5.41'' S | 51° 10' 38.19'' W |  | [27] |
| *D. aurita* | 29° 45' 37.81'' S | 51° 9' 8.28'' W |  | [27] |
| *D. aurita* | 31° 21' 28.00'' S | 51° 58' 44.00'' W |  | [27] |
| *D. aurita* | 29° 36' 0.00'' S | 50° 46' 0.00'' W |  | [63] |
| *D. aurita* | 29° 19' 60.00'' S | 49° 45' 0.00'' W | MCN 421 |  |
| *D. aurita* | 29° 20' 17.93'' S | 49° 43' 52.65'' W |  | [27] |
| *D. aurita* | 27° 41' 60.00'' S | 49° 19' 60.00'' W |  | [34] |
| *D. aurita* | 27° 53' 60.00'' S | 49° 7' 60.00'' W | UFSC 3466 |  |
| *D. aurita* | 26° 55' 0.00'' S | 49° 4' 0.00'' W |  | [34] |
| *D. aurita* | 27° 55' 3.00'' S | 48° 55' 3.00'' W |  | [37] |
| *D. aurita* | 27° 4' 0.00'' S | 53° 13' 60.00'' W |  | [34] |
| *D. aurita* | 27° 1' 60.00'' S | 48° 38' 60.00'' W | MCAGH 08 49 | [37] |
| *D. aurita* | 26° 25' 60.00'' S | 49° 15' 0.00'' W | MSCJ 51 | [37] |
| *D. aurita* | 27° 36' 47.04'' S | 48° 35' 22.86'' W | UFSC 360 |  |
| *D. aurita* | 27° 43' 3.22'' S | 48° 32' 5.27'' W |  | [64] |
| *D. aurita* | 27° 42' 57.34'' S | 48° 31' 58.77'' W |  | [65] |
| *D. aurita* | 27° 49' 30.48'' S | 48° 33' 23.36'' W |  | [36] |
| *D. aurita* | 27° 19' 0.00'' S | 48° 34' 0.00'' W | UFSC 3402 |  |
| *D. aurita* | 27° 28' 34.80'' S | 48° 33' 46.89'' W | UFSC 780 | [37] |
| *D. aurita* | 27° 35' 53.33'' S | 48° 31' 14.06'' W | UFSC 801 |  |
| *D. aurita* | 27° 35' 53.37'' S | 48° 31' 14.05'' W | UFSC 793 |  |
| *D. aurita* | 27° 36' 46.46'' S | 48° 30' 54.21'' W | UFSC 109 |  |
| *D. aurita* | 27° 36' 15.80'' S | 48° 26' 6.56'' W | UFSC 444 |  |
| *D. aurita* | 27° 16' 60.00'' S | 48° 22' 0.00'' W | UFSC 901 | [37] |
| *D. aurita* | 26° 4' 0.00'' S | 48° 37' 60.00'' W |  | [66] |
| *D. aurita* | 26° 28' 60.00'' S | 49° 4' 0.00'' W |  | [34] |
| *D. aurita* | 26° 18' 0.00'' S | 48° 51' 0.00'' W |  | [34] |
| *D. aurita* | 28° 28' 60.00'' S | 48° 46' 60.00'' W |  | [34] |
| *D. aurita* | 28° 22' 0.00'' S | 49° 16' 60.00'' W |  | [34] |
| *D. aurita* | 27° 38' 60.00'' S | 48° 40' 0.00'' W |  | [34] |
| *D. aurita* | 27° 40' 0.00'' S | 49° 1' 0.00'' W |  | [34] |
| *D. aurita* | 27° 43' 0.00'' S | 48° 47' 60.00'' W | UFSC 3452 |  |
| *D. aurita* | 26° 15' 0.00'' S | 49° 22' 60.00'' W |  | [34] |
| *D. aurita* | 28° 10' 33.00'' S | 49° 10' 36.00'' W |  | [37] |
| *D. aurita* | 28° 36' 0.00'' S | 49° 32' 60.00'' W |  | [35] |
| *D. aurita* | 26° 6' 0.00'' S | 50° 19' 0.00'' W |  | [38] |
| *D. aurita* | 26° 11' 60.00'' S | 50° 12' 0.00'' W |  | [67] |
| *D. aurita* | 23° 51' 15.98'' S | 46° 8' 22.40'' W | MZUSP 10053 |  |
| *D. aurita* | 23° 45' 43.18'' S | 45° 55' 49.54'' W |  | [13] |
| *D. aurita* | 22° 53' 25.14'' S | 48° 27' 18.59'' W | MN 72363 |  |
| *D. aurita* | 24° 15' 56.35'' S | 48° 24' 49.18'' W | MZUSP 30977 |  |
| *D. aurita* | 23° 6' 2.50'' S | 45° 42' 22.64'' W | MN 23730 |  |
| *D. aurita* | 22° 54' 34.14'' S | 47° 3' 46.32'' W | MZUSP 10000 |  |
| *D. aurita* | 25° 8' 40.77'' S | 47° 57' 54.19'' W | MZUSP 27763 |  |
| *D. aurita* | 23° 37' 22.71'' S | 45° 24' 42.55'' W | MZUSP 9786 |  |
| *D. aurita* | 23° 36' 58.76'' S | 45° 56' 59.94'' W | MZUSP 12875 |  |
| *D. aurita* | 23° 0' 34.00'' S | 47° 59' 22.13'' W | MZUSP 13755 |  |
| *D. aurita* | 23° 43' 9.00'' S | 47° 3' 2.00'' W |  | [68] |
| *D. aurita* | 23° 41' 51.76'' S | 46° 57' 52.93'' W |  | [68] |
| *D. aurita* | 23° 36' 33.22'' S | 46° 55' 28.95'' W | MZUSP 9697 |  |
| *D. aurita* | 22° 14' 23.42'' S | 47° 49' 51.02'' W |  | [69] |
| *D. aurita* | 23° 59' 40.19'' S | 46° 15' 25.07'' W | UFSC 445 |  |
| *D. aurita* | 23° 43' 43.03'' S | 47° 4' 54.61'' W |  | [70] |
| *D. aurita* | 23° 33' 2.67'' S | 47° 58' 59.37'' W | MZUSP 16543 |  |
| *D. aurita* | 23° 42' 58.79'' S | 49° 29' 27.18'' W | MZUSP 17373 |  |
| *D. aurita* | 20° 20' 6.53'' S | 47° 47' 47.83'' W | MZUSP 2991 |  |
| *D. aurita* | 23° 45' 45.68'' S | 45° 47' 22.98'' W | MZUSP 27440 |  |
| *D. aurita* | 24° 14' 24.12'' S | 48° 4' 8.18'' W |  | [71] |
| *D. aurita* | 22° 43' 21.78'' S | 46° 55' 34.06'' W |  | [12] |
| *D. aurita* | 23° 41' 51.77'' S | 47° 37' 51.54'' W | MZUSP 16520 |  |
| *D. aurita* | 24° 18' 57.86'' S | 47° 51' 14.96'' W | MZUSP 16589 |  |
| *D. aurita* | 23° 46' 49.03'' S | 46° 31' 45.57'' W | MZUSP 30667 |  |
| *D. aurita* | 23° 37' 51.83'' S | 45° 52' 10.90'' W | MZUSP 16572 |  |
| *D. aurita* | 23° 46' 48.42'' S | 46° 31' 46.16'' W | MZUSP 30648 |  |
| *D. aurita* | 22° 53' 59.94'' S | 45° 58' 0.15'' W | MZUSP 6546 |  |
| *D. aurita* | 23° 42' 5.49'' S | 46° 41' 48.98'' W | MZUSP 10321 |  |
| *D. aurita* | 23° 38' 3.78'' S | 46° 37' 33.51'' W | MZUSP 16431 |  |
| *D. aurita* | 23° 35' 32.54'' S | 46° 36' 27.85'' W | MZUSP 2495 |  |
| *D. aurita* | 23° 31' 37.72'' S | 47° 8' 3.57'' W | MZUSP 24820 |  |
| *D. aurita* | 24° 18' 57.19'' S | 47° 51' 16.25'' W | MZUSP 10427 |  |
| *D. aurita* | 23° 1' 51.28'' S | 45° 32' 53.67'' W | MN 23731 |  |
| *D. aurita* | 23° 26' 22.18'' S | 45° 5' 9.00'' W | MZUSP 1809 |  |
| *D. aurita* | 24° 15' 56.35'' S | 48° 24' 49.18'' W | MZUSP 27246 |  |
| *D. aurita* | 27° 22' 41.78'' S | 54° 44' 38.00'' W |  | [42] |
| *D. aurita* | 26° 23' 59.71'' S | 54° 37' 36.52'' W |  | [42] |
| *D. aurita* | 25° 46' 60.00'' S | 54° 1' 60.00'' W |  | [43] |
| *D. aurita* | 26° 4' 60.00'' S | 53° 46' 0.00'' W |  | [43] |
| *D. aurita* | 27° 7' 60.00'' S | 53° 53' 60.00'' W | SNOMNH 23495 | [43] |
| *D. aurita* | 25° 36' 0.00'' S | 54° 34' 60.00'' W |  | [43] |
| *D. aurita* | 25° 38' 60.00'' S | 54° 19' 60.00'' W |  | [43] |
| *D. aurita* | 23° 53' 60.00'' S | 56° 34' 0.00'' W |  | [43] |

**References from S1 Table**

1. Asfora PH, Pontes AR. The small mammals of the highly impacted north-eastern Atlantic Forest of Brazil, Pernambuco endemism center. Biota Neotrop. 2009; 9: 31–36.

2. Pereira LG, Geise L. Non-flying mammals of Chapada Diamantina (Bahia, Brazil). Biota Neotrop. 2009; 9: 185-196.

3. Freitas RR, Rocha PLB, Simões-Lopes PC. Habitat structure and small mammals abundances in one semiarid landscape in the Brazilian Caatinga. Rev Bras Zool. 2005; 22: 119-129.

4. Cerqueira R. Reproduction de *Didelphis albiventris* dans le nord-est du Brésil (Polyprotodontia, Didelphidae). Mammalia. 1984; 48: 95-104.

5. Carmignotto AP, Aires CC. Mamíferos não voadores (Mammalia) da Estação Ecológica Serra Geral do Tocantins. Biota Neotrop. 2011; 11: 313-328.

6. Lemos B, Cerqueira R. Morphological differentiation in the white-eared opossum group (Didelphidae: *Didelphis*). J Mammal. 2002; 83: 354-3692.

7. Guedes PG, Silva SSP, Camardella AR, Abreu MFG, Borges-Nojosa DM, Silva JAG, et al. Diversidade de mamíferos do Parque Nacional de Ubajara (Ceará, Brasil). Mastozool Neotrop. 2000; 7: 95-100.

8. Mares MA, Ernest KA. Population and community ecology of small mammals in a gallery forest of central Brazil. J Mammal. 1995; 76: 750-768.

9. Cáceres NC, Casella J, Vargas CF, Prates LZ, Tombini AAM, Goulart CS, et al. Distribuição geográfica de pequenos mamíferos não voadores nas bacias dos rios Araguaia e Paraná, região centro-sul do Brasil. Iheringia Ser. Zool. 2008; 98: 173-180.

10. Bonvicino CR, Cerqueira R, Soraes VA. Habitat use by small mammals of upper Araguaia River. Rev Bras Biol. 1996; 56: 761-767.

11. Carvalho BA, Oliveira LFB, Nunes AP, Mattevi MS. Karyotypes of nineteen marsupial species from Brazil. J Mammal. 2002; 83: 58-70.

12. Bonvicino CR, Lindbergh SM, Maroja LS. small non-flying mammals from conserved and altered areas of Atlantic Forest and Cerrado: comments on their potential use for monitoring environment. Braz J Biol. 2002; 62: 765-774.

13. Gardner AL. Mammals of South America, Volume 1: marsupials, xenarthrans, shrews, and bats. Chicago: The University of Chicago Press; 2008.

14. Oliveira FFR, Nessim R, Costa LP, Leite YLR. Small mammal ecology in an urban atlantic forest fragment in southeastern Brazil. Lundiana. 2007; 8: 27-34.

15. Rocha MF, Passamani M, Louzada J. A small mammal community in a forest fragment, vegetation corridor and coffee matrix system in the Brazilian Atlantic Forest. PLoS One. 2011; 6: e23312. doi: 10.1371/journal.pone.0023312.

16. Cáceres NC, Cherem J, Graipel ME. Distribuição geográfica de mamíferos terrestres na região sul do Brasil. C & A. 2007; 35: 167-180.

17. Cáceres NC, Godoi MN, Hannibal W, Ferreira VL. Effects of altitude and vegetation on small-mammal distribution in the Urucum Mountain, western Brazil. J Trop Ecol. 2011; 27: 279-287.

18. Alho CJR, Lacher Jr TE, Campos ZMS, Gonçalves HC. Mamíferos da Fazenda Nhumirim, sub-região de Nhecolândia, Pantanal do Mato Grosso do Sul. I – Levantamento preliminar de espécies. Rev Bras Zool. 1987; 4: 151-164.

19. Melo ES, Santos-Filho M. Efeitos da BR-070 na província serrana de Cáceres, Mato Grosso, sobre a comunidade de vertebrados silvestres. Rev Bras de Zoociênc. 2007; 9: 185-192.

20. Aléssio FM, Pontes ARM, Silva VL. Feeding by *Didelphis albiventris* on tree gum in the northeastern Atlantic Forest of Brazil. Mastozool Neotrop. 2005; 12: 53-56.

21. Cáceres NC. Food Habits and Seed Dispersal by the White-Eared Opossum *Didelphis albiventris* in Southern Brazil. Stud Neotrop Fauna Environ. 2002; 37: 97-104.

22. Gazarini J, Brito JEC, Bernardi IP. Predação oportunista de morcegos por *Didelphis albiventris* no sul do Brasil. Chiropt Neotrop. 2008; 14: 408-411.

23. Persson VG, Lorini ML. Contribuição ao Conhecimento mastofaunístico da porção centro-sul do estado do Paraná. Acta Biol Leopold. 1990; 12: 79-98.

24. Reis NR, Perachi AL, Fandiño-Mariño H, Rocha VJ. Mamíferos da Fazenda Monte Alegre, Paraná. Londrina: Editora da Universidae Estadual de Londrina; 2005.

25. Quadros J, Cáceres NC, Tiepolo LM, Wängler MS. Mastofauna do Parque Estadual do Rio Guarani e área de influência da Usina Hidrelétrica de Salto Caxias, baixo Rio Iguaçu, Estado do Paraná, Brasil. II CBUC. 2000; 822-829.

26. González EM, Claramunt SJ, Saralegui AM. Mamíferos hallados en egagrópilas de *Tyto alba* (Aves, Strigiformes, Tytonidae) en Bagé, Rio Grande do Sul, Brasil. Iheringia Ser. Zool. 1999; 86: 117-120.

27. Queirolo D. Diversidade e padrões de distribuição de mamíferos dos pampas do Uruguai e Brasil. PhD Thesis, Universidade de São Paulo. 2009. Available: http://www.teses.usp.br/teses/disponiveis/41/41134/tde-10092009-144552/pt-br.php

28. Abreu MSL, Cristoff AU, Vieira EM. Identificação de marsupiais do Rio Grande do Sul através da microestrutura dos pelos-guarda. Biota Neotrop. 2011; 11: 391-400.

29. Kasper CB, Feldens MJ, Mazim FD, Schneider A, Cademartori CV, César H, Grillo Z. Mammals of the Taquari Valley, Central Region of Rio Grande do Sul State. Biociências. 2007; 15: 53-62.

30. Coelho, I.P., Kindel, A. & Coelho, A.V.P. (2008) Roadkills of vertebrate species on two highways through the Atlantic Forest Biosphere Reserve, southern Brazil. Eur J Wildl Res. 2008; 54: 689-699.

31. Sponchiado J, Melo GL, Cáceres NC. Habitat selection by small mammals in Brazilian Pampas biome. J Nat Hist. 2012; 46: 1321-1335.

32. Horn GB. A assembléia de pequenos mamíferos da floresta paludosa do faxinal, Torres, RS: sua relação com a borda e o roedor *Akodon montensis* (Rodentia, Muridae) como potenical dispersor de sementes endozoocóricas. M.Sc. Dissertation, Universidade Federal do Rio Grande do Sul. 2005. Available: http://hdl.handle.net/10183/6869

33. Scheibler DR, Christoff AU. Habitat associations of small mammals in southern Brazil and use of regurgitated pellets of birds of prey for inventorying a local fauna. Braz J Biol. 2007; 67: 619-625.

34. Cimardi AV. Mamíferos de Santa Catarina. Florianópolis: Fundação de Amparo à Tecnologia e Meio Ambiente; 1996.

35. Cherem JJ. Registros de mamíferos não-voadores em estudos de avaliação ambiental no sul do Brasil. Biotemas. 2005; 18: 169-202.

36. Oliveira-Santos LGR, Tortato MA, Graipel ME. Activity pattern of Atlantic Forest small arboreal mammals as revealed by camera traps. J Trop Ecol. 2008; 24: 563-567.

37. Cherem JJ, Simões-Lopes PC, Althoff S, Graipel ME. Lista dos mamíferos do estado de Santa Catarina, Sul do Brasil. Mastozool Neotrop. 2004; 11: 151-184.

38. Cherem JJ, Perez DM. Mamíferos Terrestres de Floresta de Araucária no Município de Três Barras, Santa Catarina, Brasil. Biotemas. 1996; 9: 29-46.

39. Fleury M, Galetti M. Forest fragment size and microhabitat effects on palm seed predation. Biol Conserv. 2006; 131: 1-13.

40. Gheler-Costa C, Verdade LM, Almeida AF. Mamíferos não-voadores do campus Luiz de Queiroz, Universidade de São Paulo, Piracicaba, Brasil. Rev Bras Zool. 2002; 19: 203-214.

41. Bezerra AMR, Carmignotto AP, Rodrigues FHG. Small non-volant mammals of an ecotone region between the Cerrado hotspot and the Amazonian Rainforest, with comments on their taxonomy and distribution. Zool Stud. 2009; 48: 861-874.

42. Chebez JC. Fauna Misionera: catálogo sistemático y zoogeográfico de los vertebrados de la Provincia de Misiones (Argentina). Buenos Aires: LOLA; 1996.

43. Flores DA, Díaz MM, Barquez RM. Systematics and Distribution of Marsupials in Argentina: A Review. In: Kelt DA, Lessa EP, Salazar-Bravo j, Patton JL, editors. The quintessential naturalist: honoring the life and legacy of Oliver P. Pearson. Berkeley: University of California publications in zoology; 2007, pp. 579-670.

44. Saenz NUS. Effects of habitat fragmentation on non-volant small mammals of the interior atlantic forest of eastern Paraguay. PhD dissertation, Texas Tech University. 2010. Available: http://www.redalyc.org/articulo.oa?id=45719986017

45. Jansa SA, Voss RS. Phylogenetic Studies on Didelphid Marsupials I. Introduction and Preliminary Results from Nuclear IRBP Gene Sequences. J Mamm Evol. 2000; 7: 43-77.

46. Pardini R. Effects of forest fragmentation on small mammals in an Atlantic Forest landscape. Biodivers Conserv. 2004; 13: 2567-2586.

47. Tonini JPR, Carão LM, Pinto IS, Gasparini JL, Leite YLR, Costa LP. Non-volant tetrapods from Reserva Biológica de Duas Bocas, state of Espírito Santo, Southeastern Brazil. Biota Neotrop. 2010; 10: 339-351.

48. Chiarello AG. Effects of fragmentation of the Atlantic forest on mammal communities in south-eastern Brazil. Biol Conserv. 1999; 89: 71-82.

49. Passamani M, Mendes SL, Chiarello AG. Non volant mammals of the Estação Biológica de Santa Lúcia and adjacent áreas of Santa Teresa, Espírito Santo, Brazil. Bol Mus Biol Mello Leitão. 2000; 11/12: 201-214.

50. Caldara Jr. V, Leite YLR. Uso de habitats por pequenos mamíferos no Parque Estadual da Fonte Grande, Vitória, Espírito Santo, Brasil. Bol Mus Biol Mello Leitão. 2007; 21: 57-77.

51. Fonseca GAB. Small mammal species diversity in brazilian tropical primary and secondary forests of different sizes. Rev Bras Zool. 1989; 6: 381-422.

52. Cáceres NC. Use of the space by the opossum *Didelphis aurita* Wied-Newied (Mammalia, Marsupialia) in a mixed forest fragment of southern Brazil. Rev Bras Zool. 2003; 20: 315-322.

53. Cáceres NC. Population Ecology and Reproduction of the White-Eared Opossum, *Didelphis albiventris* (Mammalia, Marsupialia), in an Urban Environment of Southern Brazil. Cienc Cult. 2000; 52: 171-174.

54. Cáceres NC. Diet of three didelphid marsupials (Mammalia, Didelphimorphia) in southern Brazil. Mamm Biol. 2004; 69: 430-433.

55. Freitas SR, Moraes DA, Santori RT, Cerqueira R. Habitat preference and food use by *Metachirus nudicaudatus* and *Didelphis aurita* (Didelphimorphia, Didelphidae) in a restinga forest at Rio de Janeiro. Rev Bras Biol. (1997; 57: 93-98.

56. Prevedello JA, Vieira MV. Plantation rows as dispersal routes: a test with didelphid marsupials in the Atlantic Forest, Brazil. Biol Conserv. 2010; 143: 131-135.

57. Kajin M, Cerqueira R, Vieira MV, Gentile R. Nine-year demography of the black-eared opossum *Didelphis aurita* using life tables. Rev Bras Zool. 2008; 25: 206-213.

58. Vieira MV. Body size and form in two Neotropical marsupials, *Didelphis aurita* and *Philander opossum* (Marsupialia, Didelphidae). Mammalia. 1997; 61: 245-254.

59. Galliez M, Leite MS, Queiroz TL, Fernandez AS. Ecology of the water opossum *Chironectes minimus* in Atlantic forest streams of southeastern Brazil. J Mammal. 2009; 90: 93-103.

60. Pires AS, Lira PK, Fernandez FAS, Schittini GM, Oliveira LC. Frequency of movements of small mammals among Atlantic Coastal Forest fragments in Brazil. Biol Conserv. 2002 108, 229–237.

61. D’Andrea PS, Gentile R, Cerqueira R, Grelle CEV, Horta C, Rey L. Ecology of small mammals in a Brazilian rural area. Rev Bras Zool. 1999; 16: 611-620.

62. Melo GL, Sponchiado J, Machado AF, Cácares NC. Small-mammal community structure in a South American deciduous Atlantic Forest. Community Ecol. 2011; 12: 58-66.

63. Ávila-Pires FD. Mamíferos descritos do Estado do Rio Grande do Sul, Brasil. Rev Bras Biol. 1994; 54: 367-384.

64. Cáceres NC, Prates LZ, Ghizoni Jr IR, Graipel ME. Frugivory by the black-eared opossum *Didelphis aurita* in the Atlantic Forest of southern Brazil: Roles of sex, season and sympatric species. Biotemas*.* 2009; 22: 203-211.

65. Graipel ME, Cherem JJ, Monteiro-Filho ELA, Glock L. Dinâmica populacional de marsupiais e roedores no Parque Municipal da Lagoa do Peri, Ilha de Santa Catarina, sul do Brasil. Mastozool Neotrop. 2006; 13: 31-49.

66. Quadros J, Cáceres NC. Ecologia e conservação de mamíferos na Reserva Volta Velha, estado de Santa Catarina, Brasil. Acta Biol Leopold. 2001; 23: 213-224.

67. Wallauer JP, Becker M, Martins-Sá LG, Liermann LM, Perretto SH, Schermacket V. Levantamento dos mamíferos da Floresta Nacional de Três Barras - Santa Catarina. Biotemas. 2000; 13: 103-107.

68. Pardini R, Umetsu F. Pequenos mamíferos da Reserva Florestal do Morro Grande distribuição das espécies e da diversidade em uma área de Mata Atlântica. Biota Neotrop. 2006; 6: 1-22.

69. Vieira MV, Grelle CEV, Gentile R. Differential trappability of small mammals in three habitats of southeastern Brazil. Braz J Biol. 2005; 64: 895-900.

70. Umetsu F, Metzger JP, Pardini R. Importance of estimating matrix quality for modeling species distribution in complex tropical landscapes: a test with Atlantic forest small mammals. Ecography. 2008; 31: 359-370.

71. Vieira EM, Izar P. Interactions between aroids and arboreal mammals in the Brazilian Atlantic rainforest. Plant Ecol. 1999; 145: 75-82.
