# Supplementary material for: Which Factors Determine Spatial Segregation in the South American Opossums (Didelphis aurita and D. albiventris)? An Ecological Niche Modelling and Geometric Morphometrics Approach
Source: PLoS One. 2016 Jun 23;11(6):e0157723. doi: 10.1371/journal.pone.0157723 (PMC4919065; doi:10.1371/journal.pone.0157723)
Supplement: S2 Table — (DOCX) [file pone.0157723.s005.docx]

**S2 Table.** *Didelphis albiventris* and *D. aurita* specimens used for morphological analyses, separated by museum record, species, sex, geographical coordinates (in decimal degrees) and geography.

| Museum Record | Species | Sex | Latitude | Longitude | Geography |
| --- | --- | --- | --- | --- | --- |
| MN 72152 | *D. albiventris* | Female | -22.8840 | -48.4444 | Sympatric |
| MN 72153 | *D. albiventris* | Female | -22.8840 | -48.4444 | Sympatric |
| MN 72221 | *D. albiventris* | Female | -22.8840 | -48.4444 | Sympatric |
| MN 4486 | *D. albiventris* | Female | -21.6111 | -55.1683 | Allopatric |
| MN 4493 | *D. albiventris* | Female | -21.6111 | -55.1683 | Allopatric |
| MN 4562 | *D. albiventris* | Female | -21.6111 | -55.1683 | Allopatric |
| MN 22957 | *D. albiventris* | Female | -20.7221 | -46.6133 | Sympatric |
| MN 22981 | *D. albiventris* | Female | -20.7221 | -46.6133 | Sympatric |
| MN 23637 | *D. albiventris* | Female | -20.7221 | -46.6133 | Sympatric |
| MN 4229 | *D. albiventris* | Female | -19.6327 | -43.8983 | Sympatric |
| FZB 506 | *D. albiventris* | Female | -28.2902 | -53.4909 | Sympatric |
| MHNCI 825 | *D. albiventris* | Female | -25.5470 | -49.8916 | Sympatric |
| MHNCI 2661 | *D. albiventris* | Female | -25.4284 | -49.2733 | Sympatric |
| MHNCI 2663 | *D. albiventris* | Female | -25.4284 | -49.2733 | Sympatric |
| MHNCI 3742 | *D. albiventris* | Female | -25.4284 | -49.2733 | Sympatric |
| MHNCI 3876 | *D. albiventris* | Female | -25.4284 | -49.2733 | Sympatric |
| MHNCI 1106 | *D. albiventris* | Female | -25.3698 | -50.5577 | Sympatric |
| MACN 29848 | *D. albiventris* | Female | -25.3333 | -59.7333 | Allopatric |
| MACN 36302 | *D. albiventris* | Female | -25.2833 | -64.0667 | Allopatric |
| MHNCI 316 | *D. albiventris* | Female | -24.8936 | -49.9659 | Sympatric |
| MHNCI 318 | *D. albiventris* | Female | -24.8936 | -49.9659 | Sympatric |
| MZUSP 22814 | *D. albiventris* | Female | -24.6598 | -49.0416 | Sympatric |
| MZUSP 31627 | *D. albiventris* | Female | -24.2333 | -50.9333 | Sympatric |
| MZUSP 31038 | *D. albiventris* | Female | -23.8003 | -48.5875 | Sympatric |
| MZUSP 17375 | *D. albiventris* | Female | -23.7165 | -49.4909 | Sympatric |
| MZUSP 17377 | *D. albiventris* | Female | -23.7165 | -49.4909 | Sympatric |
| MZUSP 17382 | *D. albiventris* | Female | -23.7165 | -49.4909 | Sympatric |
| MZUSP 17383 | *D. albiventris* | Female | -23.7165 | -49.4909 | Sympatric |
| MZUSP 19368 | *D. albiventris* | Female | -23.7165 | -49.4909 | Sympatric |
| MZUSP 16521 | *D. albiventris* | Female | -23.6977 | -47.6310 | Sympatric |
| MZUSP 10028 | *D. albiventris* | Female | -23.5737 | -48.0222 | Sympatric |
| MZUSP 10123 | *D. albiventris* | Female | -23.5737 | -48.0222 | Sympatric |
| MZUSP 10130 | *D. albiventris* | Female | -23.5737 | -48.0222 | Sympatric |
| MZUSP 10131 | *D. albiventris* | Female | -23.5737 | -48.0222 | Sympatric |
| MZUSP 13736 | *D. albiventris* | Female | -23.0143 | -48.0063 | Sympatric |
| MZUSP 13742 | *D. albiventris* | Female | -23.0143 | -48.0063 | Sympatric |
| MACN 36196 | *D. albiventris* | Female | -22.2395 | -63.7329 | Allopatric |
| MZUSP 2993 | *D. albiventris* | Female | -20.3353 | -47.7966 | Sympatric |
| MACN 24192 | *D. albiventris* | Male | -27.4667 | -58.8333 | Allopatric |
| MZUSP 7127 | *D. albiventris* | Male | -26.0297 | -48.8555 | Sympatric |
| MHNCI 30 | *D. albiventris* | Male | -25.4284 | -49.2733 | Sympatric |
| MHNCI 2664 | *D. albiventris* | Male | -25.4284 | -49.2733 | Sympatric |
| MACN 36299 | *D. albiventris* | Male | -25.2833 | -64.0667 | Allopatric |
| MACN 36303 | *D. albiventris* | Male | -25.2833 | -64.0667 | Allopatric |
| MACN 36307 | *D. albiventris* | Male | -25.2833 | -64.0667 | Allopatric |
| MACN 96730 | *D. albiventris* | Male | -25.2833 | -64.0667 | Allopatric |
| MHNCI 317 | *D. albiventris* | Male | -24.8936 | -49.9659 | Sympatric |
| MHNCI 319 | *D. albiventris* | Male | -24.8936 | -49.9659 | Sympatric |
| MHNCI 321 | *D. albiventris* | Male | -24.8936 | -49.9659 | Sympatric |
| MACN 25845 | *D. albiventris* | Male | -24.1172 | -58.8748 | Allopatric |
| MZUSP 13751 | *D. albiventris* | Male | -23.7165 | -49.4909 | Sympatric |
| MZUSP 13753 | *D. albiventris* | Male | -23.7165 | -49.4909 | Sympatric |
| MZUSP 17376 | *D. albiventris* | Male | -23.7165 | -49.4909 | Sympatric |
| MZUSP 17379 | *D. albiventris* | Male | -23.7165 | -49.4909 | Sympatric |
| MZUSP 17381 | *D. albiventris* | Male | -23.7165 | -49.4909 | Sympatric |
| MACN 2797 | *D. albiventris* | Male | -23.6167 | -65.4667 | Allopatric |
| MZUSP 7130 | *D. albiventris* | Male | -23.5737 | -48.0222 | Sympatric |
| MZUSP 10140 | *D. albiventris* | Male | -23.5737 | -48.0222 | Sympatric |
| MHNCI 412 | *D. albiventris* | Male | -23.4500 | -53.3833 | Sympatric |
| MACN 36753 | *D. albiventris* | Male | -23.2763 | -63.2830 | Allopatric |
| MN 25646 | *D. albiventris* | Male | -22.9071 | -47.0632 | Sympatric |
| MN 72171 | *D. albiventris* | Male | -22.8840 | -48.4444 | Sympatric |
| MN 72184 | *D. albiventris* | Male | -22.8840 | -48.4444 | Sympatric |
| MN 72229 | *D. albiventris* | Male | -22.8840 | -48.4444 | Sympatric |
| MN 50676 | *D. albiventris* | Male | -22.4100 | -47.5621 | Sympatric |
| MZUSP 3716 | *D. albiventris* | Male | -21.7640 | -52.1061 | Sympatric |
| MZUSP 3717 | *D. albiventris* | Male | -21.7640 | -52.1061 | Sympatric |
| MZUSP 3719 | *D. albiventris* | Male | -21.7640 | -52.1061 | Sympatric |
| MN 24957 | *D. albiventris* | Male | -21.6111 | -55.1683 | Allopatric |
| MZUSP 20177 | *D. albiventris* | Male | -21.1667 | -47.8149 | Sympatric |
| MN 22958 | *D. albiventris* | Male | -20.7221 | -46.6133 | Sympatric |
| MN 22961 | *D. albiventris* | Male | -20.7221 | -46.6133 | Sympatric |
| MN 20957 | *D. albiventris* | Male | -20.7221 | -46.6133 | Sympatric |
| MZUSP 3779 | *D. albiventris* | Male | -20.4435 | -54.6478 | Allopatric |
| MN 13414 | *D. albiventris* | Male | -19.6327 | -43.8983 | Sympatric |
| MN 3858 | *D. aurita* | Female | -22.9631 | -44.0414 | Allopatric |
| MN 73762 | *D. aurita* | Female | -22.9631 | -44.0414 | Allopatric |
| MN 73769 | *D. aurita* | Female | -22.9631 | -44.0414 | Allopatric |
| MN 28903 | *D. aurita* | Female | -22.9340 | -42.8246 | Allopatric |
| MN 50626 | *D. aurita* | Female | -22.9340 | -42.8246 | Allopatric |
| MN 1179 | *D. aurita* | Female | -22.9171 | -43.1974 | Allopatric |
| MN 5615 | *D. aurita* | Female | -22.9171 | -43.1974 | Allopatric |
| MN 10486 | *D. aurita* | Female | -22.9171 | -43.1974 | Allopatric |
| MN 42795 | *D. aurita* | Female | -22.6615 | -43.0312 | Allopatric |
| MN 46509 | *D. aurita* | Female | -22.6615 | -43.0312 | Allopatric |
| MN 50190 | *D. aurita* | Female | -22.5343 | -42.9898 | Allopatric |
| MN 43753 | *D. aurita* | Female | -22.0544 | -42.6766 | Allopatric |
| MN 50332 | *D. aurita* | Female | -22.0544 | -42.6766 | Allopatric |
| MN 7721 | *D. aurita* | Female | -21.8874 | -42.7046 | Allopatric |
| MN 7722 | *D. aurita* | Female | -21.8874 | -42.7046 | Allopatric |
| MN 20940 | *D. aurita* | Female | -21.7629 | -43.3417 | Allopatric |
| MN 5718 | *D. aurita* | Female | -19.9434 | -40.5965 | Allopatric |
| UFSC801 | *D. aurita* | Female | -27.5981 | -48.5206 | Sympatric |
| UFSC830 | *D. aurita* | Female | -27.5981 | -48.5206 | Sympatric |
| MHNCI 3881 | *D. aurita* | Female | -26.0998 | -49.4268 | Sympatric |
| MZUSP 9644 | *D. aurita* | Female | -25.8844 | -48.5762 | Sympatric |
| MHNCI 28 | *D. aurita* | Female | -25.8167 | -48.5500 | Sympatric |
| MHNCI 206 | *D. aurita* | Female | -25.6804 | -54.4340 | Sympatric |
| MHNCI 207 | *D. aurita* | Female | -25.6804 | -54.4340 | Sympatric |
| MHNCI 4326 | *D. aurita* | Female | -25.3222 | -49.1579 | Sympatric |
| MZUSP 3906 | *D. aurita* | Female | -25.1447 | -47.9651 | Sympatric |
| MZUSP 27763 | *D. aurita* | Female | -25.1447 | -47.9651 | Sympatric |
| MHNCI 2662 | *D. aurita* | Female | -25.1204 | -50.1720 | Sympatric |
| MZUSP 16595 | *D. aurita* | Female | -24.3161 | -47.8542 | Sympatric |
| MZUSP 16596 | *D. aurita* | Female | -24.3161 | -47.8542 | Sympatric |
| MZUSP 9663 | *D. aurita* | Female | -23.8544 | -46.1396 | Allopatric |
| MZUSP 30667 | *D. aurita* | Female | -23.7803 | -46.5293 | Allopatric |
| MZUSP 30768 | *D. aurita* | Female | -23.7803 | -46.5293 | Allopatric |
| MZUSP 27439 | *D. aurita* | Female | -23.7627 | -45.7897 | Allopatric |
| MZUSP 27440 | *D. aurita* | Female | -23.7627 | -45.7897 | Allopatric |
| MZUSP 17371 | *D. aurita* | Female | -23.7165 | -49.4909 | Sympatric |
| MZUSP 17373 | *D. aurita* | Female | -23.7165 | -49.4909 | Sympatric |
| MZUSP 17374 | *D. aurita* | Female | -23.7165 | -49.4909 | Sympatric |
| MZUSP 17375 | *D. aurita* | Female | -23.7165 | -49.4909 | Sympatric |
| MZUSP 10321 | *D. aurita* | Female | -23.7015 | -46.6969 | Sympatric |
| MZUSP 10323 | *D. aurita* | Female | -23.7015 | -46.6969 | Sympatric |
| MZUSP 10324 | *D. aurita* | Female | -23.7015 | -46.6969 | Sympatric |
| MZUSP 10325 | *D. aurita* | Female | -23.7015 | -46.6969 | Sympatric |
| MZUSP 16572 | *D. aurita* | Female | -23.6311 | -45.8697 | Allopatric |
| MZUSP 9786 | *D. aurita* | Female | -23.6230 | -45.4118 | Allopatric |
| MZUSP 9787 | *D. aurita* | Female | -23.6230 | -45.4118 | Allopatric |
| MZUSP 16594 | *D. aurita* | Female | -23.6230 | -45.4118 | Allopatric |
| MZUSP 10138 | *D. aurita* | Female | -23.6163 | -45.9500 | Allopatric |
| MZUSP 12874 | *D. aurita* | Female | -23.6163 | -45.9500 | Allopatric |
| MZUSP 16440 | *D. aurita* | Female | -23.6163 | -45.9500 | Allopatric |
| MZUSP 9647 | *D. aurita* | Female | -23.6092 | -46.9247 | Sympatric |
| MZUSP 9652 | *D. aurita* | Female | -23.6092 | -46.9247 | Sympatric |
| MZUSP 9653 | *D. aurita* | Female | -23.6092 | -46.9247 | Sympatric |
| MZUSP 9656 | *D. aurita* | Female | -23.6092 | -46.9247 | Sympatric |
| MZUSP 9697 | *D. aurita* | Female | -23.6092 | -46.9247 | Sympatric |
| MZUSP 3019 | *D. aurita* | Female | -23.5924 | -46.6077 | Sympatric |
| MZUSP 12855 | *D. aurita* | Female | -23.5737 | -48.0222 | Sympatric |
| MZUSP 16543 | *D. aurita* | Female | -23.5737 | -48.0222 | Sympatric |
| MZUSP 24820 | *D. aurita* | Female | -23.5271 | -47.1343 | Sympatric |
| MZUSP 24822 | *D. aurita* | Female | -23.5271 | -47.1343 | Sympatric |
| MZUSP 1809 | *D. aurita* | Female | -23.4395 | -45.0858 | Allopatric |
| MZUSP 13755 | *D. aurita* | Female | -23.0143 | -48.0063 | Sympatric |
| MZUSP 13792 | *D. aurita* | Female | -23.0143 | -48.0063 | Sympatric |
| MZUSP 10002 | *D. aurita* | Female | -22.9071 | -47.0632 | Sympatric |
| MZUSP 9650 | *D. aurita* | Female | -22.1931 | -48.7812 | Sympatric |
| MZUSP 9651 | *D. aurita* | Female | -22.1931 | -48.7812 | Sympatric |
| MZUSP 6204 | *D. aurita* | Female | -19.9383 | -40.5959 | Allopatric |
| UFSC 109 | *D. aurita* | Male | -27.6129 | -48.5151 | Sympatric |
| UFSC 444 | *D. aurita* | Male | -27.6044 | -48.4352 | Sympatric |
| UFSC 793 | *D. aurita* | Male | -27.5982 | -48.5206 | Sympatric |
| UFSC 802 | *D. aurita* | Male | -27.5981 | -48.5206 | Sympatric |
| MACN 49345 | *D. aurita* | Male | -26.4500 | -54.4667 | Sympatric |
| MACN 49392 | *D. aurita* | Male | -26.4500 | -54.4667 | Sympatric |
| MACN 49412 | *D. aurita* | Male | -26.4500 | -54.4667 | Sympatric |
| MACN 49454 | *D. aurita* | Male | -26.4500 | -54.4667 | Sympatric |
| MHNCI 246 | *D. aurita* | Male | -25.8844 | -48.5762 | Sympatric |
| MZUSP 9643 | *D. aurita* | Male | -25.8844 | -48.5762 | Sympatric |
| MHNCI 331 | *D. aurita* | Male | -25.8289 | -48.5411 | Sympatric |
| MACN 15432 | *D. aurita* | Male | -25.7833 | -54.0333 | Sympatric |
| MHNCI 280 | *D. aurita* | Male | -25.6804 | -54.4340 | Sympatric |
| MHNCI 335 | *D. aurita* | Male | -25.6779 | -49.5197 | Sympatric |
| MACN 25807 | *D. aurita* | Male | -25.6500 | -54.3333 | Sympatric |
| MHNCI 29 | *D. aurita* | Male | -25.5861 | -48.6166 | Sympatric |
| MHNCI 2654 | *D. aurita* | Male | -25.5322 | -49.3944 | Sympatric |
| MHNCI 2657 | *D. aurita* | Male | -25.5322 | -49.3944 | Sympatric |
| MHNCI 6191 | *D. aurita* | Male | -25.4458 | -49.5013 | Sympatric |
| MHNCI 3875 | *D. aurita* | Male | -25.4333 | -49.3039 | Sympatric |
| MHNCI 3879 | *D. aurita* | Male | -25.4333 | -49.3039 | Sympatric |
| MHNCI 129 | *D. aurita* | Male | -25.3333 | -49.1667 | Sympatric |
| MZUSP 10631 | *D. aurita* | Male | -24.3844 | -47.9280 | Sympatric |
| MZUSP 16589 | *D. aurita* | Male | -24.3161 | -47.8542 | Sympatric |
| MZUSP 10427 | *D. aurita* | Male | -24.3159 | -47.8545 | Sympatric |
| MZUSP 27246 | *D. aurita* | Male | -24.2657 | -48.4137 | Sympatric |
| MZUSP 27247 | *D. aurita* | Male | -24.2657 | -48.4137 | Sympatric |
| MZUSP 27249 | *D. aurita* | Male | -24.2657 | -48.4137 | Sympatric |
| MZUSP 30977 | *D. aurita* | Male | -24.2657 | -48.4137 | Sympatric |
| MZUSP 10053 | *D. aurita* | Male | -23.8544 | -46.1396 | Allopatric |
| MZUSP 30648 | *D. aurita* | Male | -23.7801 | -46.5295 | Sympatric |
| MZUSP 31381 | *D. aurita* | Male | -23.7798 | -50.0355 | Sympatric |
| MZUSP 17365 | *D. aurita* | Male | -23.7165 | -49.4909 | Sympatric |
| MZUSP 17366 | *D. aurita* | Male | -23.7165 | -49.4909 | Sympatric |
| MZUSP 17368 | *D. aurita* | Male | -23.7165 | -49.4909 | Sympatric |
| MZUSP 17370 | *D. aurita* | Male | -23.7165 | -49.4909 | Sympatric |
| MZUSP 10335 | *D. aurita* | Male | -23.7015 | -46.6969 | Sympatric |
| MZUSP 16578 | *D. aurita* | Male | -23.6977 | -47.6310 | Sympatric |
| MZUSP 16431 | *D. aurita* | Male | -23.6344 | -46.6260 | Sympatric |
| MZUSP 10139 | *D. aurita* | Male | -23.6163 | -45.9500 | Allopatric |
| MZUSP 12875 | *D. aurita* | Male | -23.6163 | -45.9500 | Allopatric |
| MZUSP 16438 | *D. aurita* | Male | -23.6163 | -45.9500 | Allopatric |
| MZUSP 16442 | *D. aurita* | Male | -23.6163 | -45.9500 | Allopatric |
| MZUSP 9646 | *D. aurita* | Male | -23.6092 | -46.9247 | Sympatric |
| MZUSP 9648 | *D. aurita* | Male | -23.6092 | -46.9247 | Sympatric |
| MZUSP 9666 | *D. aurita* | Male | -23.6092 | -46.9247 | Sympatric |
| MZUSP 9668 | *D. aurita* | Male | -23.6092 | -46.9247 | Sympatric |
| MZUSP 9669 | *D. aurita* | Male | -23.6092 | -46.9247 | Sympatric |
| MZUSP 2573 | *D. aurita* | Male | -23.5924 | -46.6077 | Sympatric |
| MZUSP 24821 | *D. aurita* | Male | -23.5271 | -47.1343 | Sympatric |
| MZUSP 24824 | *D. aurita* | Male | -23.5271 | -47.1343 | Sympatric |
| MZUSP 24825 | *D. aurita* | Male | -23.5271 | -47.1343 | Sympatric |
| MZUSP 24826 | *D. aurita* | Male | -23.5271 | -47.1343 | Sympatric |
| MZUSP 13749 | *D. aurita* | Male | -23.0143 | -48.0063 | Sympatric |
| MZUSP 13774 | *D. aurita* | Male | -23.0143 | -48.0063 | Sympatric |
| MN 3854 | *D. aurita* | Male | -22.9631 | -44.0414 | Allopatric |
| MN 3860 | *D. aurita* | Male | -22.9631 | -44.0414 | Allopatric |
| MN 73770 | *D. aurita* | Male | -22.9631 | -44.0414 | Allopatric |
| MN 5688 | *D. aurita* | Male | -22.9171 | -43.1974 | Allopatric |
| MN 10483 | *D. aurita* | Male | -22.9171 | -43.1974 | Allopatric |
| MN 10485 | *D. aurita* | Male | -22.9171 | -43.1974 | Allopatric |
| MZUSP 10000 | *D. aurita* | Male | -22.9071 | -47.0632 | Sympatric |
| MZUSP 6546 | *D. aurita* | Male | -22.9000 | -45.9667 | Allopatric |
| MN 3856 | *D. aurita* | Male | -22.8426 | -44.0535 | Allopatric |
| MN 46503 | *D. aurita* | Male | -22.6615 | -43.0312 | Allopatric |
| MN 50370 | *D. aurita* | Male | -22.6615 | -43.0312 | Allopatric |
| MZUSP 12862 | *D. aurita* | Male | -22.6449 | -47.4257 | Sympatric |
| MN 51927 | *D. aurita* | Male | -22.6263 | -43.9057 | Allopatric |
| MN 75967 | *D. aurita* | Male | -22.6263 | -43.9057 | Allopatric |
| MZUSP 2808 | *D. aurita* | Male | -22.3174 | -42.3337 | Allopatric |
| MZUSP 9706 | *D. aurita* | Male | -22.1931 | -48.7812 | Sympatric |
| MZUSP 12856 | *D. aurita* | Male | -22.1931 | -48.7812 | Sympatric |
| MZUSP 12858 | *D. aurita* | Male | -22.1931 | -48.7812 | Sympatric |
| MZUSP 12859 | *D. aurita* | Male | -22.1931 | -48.7812 | Sympatric |
| MZUSP 12878 | *D. aurita* | Male | -22.1931 | -48.7812 | Sympatric |
| MN 3850 | *D. aurita* | Male | -21.8874 | -42.7046 | Allopatric |
| MN 3852 | *D. aurita* | Male | -21.8874 | -42.7046 | Allopatric |
| MN 3853 | *D. aurita* | Male | -21.8874 | -42.7046 | Allopatric |
| MN 23478 | *D. aurita* | Male | -21.7881 | -46.5633 | Sympatric |
| MN 3851 | *D. aurita* | Male | -21.7685 | -42.5378 | Allopatric |
| MN 20938 | *D. aurita* | Male | -21.7629 | -43.3417 | Allopatric |
| MN 20941 | *D. aurita* | Male | -21.7629 | -43.3417 | Allopatric |
| MN 50679 | *D. aurita* | Male | -21.7629 | -43.3417 | Allopatric |
| MN 23758 | *D. aurita* | Male | -20.3614 | -40.6596 | Allopatric |
| MN 20946 | *D. aurita* | Male | -20.3519 | -40.2997 | Allopatric |
| MN 20948 | *D. aurita* | Male | -20.3519 | -40.2997 | Allopatric |
| MZUSP 2989 | *D. aurita* | Male | -20.3353 | -47.7966 | Sympatric |
| MZUSP 2990 | *D. aurita* | Male | -20.3353 | -47.7966 | Sympatric |
| MN 11671 | *D. aurita* | Male | -20.1243 | -40.3062 | Allopatric |
| MN 5720 | *D. aurita* | Male | -19.9434 | -40.5965 | Allopatric |
| MN 20947 | *D. aurita* | Male | -19.9434 | -40.5965 | Allopatric |
| UFSC 2407 | *D. aurita* | Male | -19.7963 | -42.6336 | Allopatric |
