# Supplementary material for: Which Factors Determine Spatial Segregation in the South American Opossums (Didelphis aurita and D. albiventris)? An Ecological Niche Modelling and Geometric Morphometrics Approach
Source: PLoS One. 2016 Jun 23;11(6):e0157723. doi: 10.1371/journal.pone.0157723 (PMC4919065; doi:10.1371/journal.pone.0157723)
Supplement: S3 Table — P values tests for the significance of F after 1000 permutations. Significance is highlighted. (DOCX) [file pone.0157723.s006.docx]

**S3 Table.** Variation partitioning with *Didelphis* skull shape as dependent variable and taxonomy, size, sex and geography as dependent variables. P values tests for the significance of F after 1000 permutations. Significance is highlighted.

| Factor | Df | R.square | Adj.R.square | F | P |
| --- | --- | --- | --- | --- | --- |
| Species | 1 | 0.051 | 0.047 | 12.392 | **0.001** |
| Sex | 2 | 0.034 | 0.025 | 4.040 | **0.001** |
| Size | 1 | 0.027 | 0.022 | 6.304 | **0.001** |
| Geography | 1 | 0.017 | 0.012 | 3.883 | **0.007** |
| Species + Sex | 3 | 0.088 | 0.076 | 7.364 | **0.001** |
| Species + Size | 2 | 0.100 | 0.093 | 12.894 | **0.001** |
| Species + Geography | 2 | 0.060 | 0.052 | 7.398 | **0.001** |
| Sex + Size | 3 | 0.052 | 0.040 | 4.195 | **0.001** |
| Sex + Geography | 3 | 0.053 | 0.040 | 4.268 | **0.001** |
| Size + Geography | 2 | 0.045 | 0.036 | 5.376 | **0.001** |
| Species + Sex + Size | 4 | 0.123 | 0.108 | 8.049 | **0.001** |
| Species + Sex + Geography | 4 | 0.098 | 0.082 | 6.202 | **0.001** |
| Species + Size + Geography | 3 | 0.111 | 0.099 | 9.551 | **0.001** |
| Sex + Size + Geography | 4 | 0.071 | 0.055 | 4.385 | **0.001** |
| All | 5 | 0.134 | 0.115 | 7.045 | **0.001** |
| Species “Pure” | 1 |  | 0.060 | 16.494 | **0.001** |
| Sex “Pure” | 2 |  | 0.017 | 3.032 | **0.001** |
| Size “Pure” | 1 |  | 0.033 | 9.496 | **0.001** |
| Geography “Pure” | 1 |  | 0.007 | 2.779 | **0.013** |
