# Supplementary material for: Which Factors Determine Spatial Segregation in the South American Opossums (Didelphis aurita and D. albiventris)? An Ecological Niche Modelling and Geometric Morphometrics Approach
Source: PLoS One. 2016 Jun 23;11(6):e0157723. doi: 10.1371/journal.pone.0157723 (PMC4919065; doi:10.1371/journal.pone.0157723)
Supplement: S4 Table — P values tests for the significance of F after 1000 permutations. Significance is highlighted. (DOCX) [file pone.0157723.s007.docx]

**S4 Table.** Variation partitioning with *Didelphis aurita* skull shape as dependent variable and size, sex and geography as dependent variables. P values tests for the significance of F after 1000 permutations. Significance is highlighted.

| Factor | Df | R.square | Adj.R.square | F | P |
| --- | --- | --- | --- | --- | --- |
| Sex | 2 | 0.042 | 0.029 | 3.389 | **0.001** |
| Size | 1 | 0.088 | 0.082 | 15.086 | **0.001** |
| Geography | 1 | 0.016 | 0.009 | 2.550 | **0.025** |
| Sex + Size | 3 | 0.119 | 0.103 | 7.044 | **0.001** |
| Sex + Geography | 3 | 0.054 | 0.036 | 2.962 | **0.001** |
| Size + Geography | 2 | 0.106 | 0.094 | 9.235 | **0.001** |
| All | 4 | 0.131 | 0.109 | 5.816 | **0.001** |
| Sex "Pure" | 2 |  | 0.014 | 2.249 | **0.015** |
| Size "Pure" | 1 |  | 0.073 | 13.653 | **0.001** |
| Geography "Pure" | 1 |  | 0.006 | 1.997 | 0.078 |
