# Supplementary material for: Which Factors Determine Spatial Segregation in the South American Opossums (Didelphis aurita and D. albiventris)? An Ecological Niche Modelling and Geometric Morphometrics Approach
Source: PLoS One. 2016 Jun 23;11(6):e0157723. doi: 10.1371/journal.pone.0157723 (PMC4919065; doi:10.1371/journal.pone.0157723)
Supplement: S5 Table — P values tests for the significance of F after 1000 permutations. Significance is highlighted. (DOCX) [file pone.0157723.s008.docx]

**S5 Table.** Variation partitioning with *Didelphis albiventris* skull shape as dependent variable and size, sex and geography as dependent variables. P values tests for the significance of F after 1000 permutations. Significance is highlighted.

| Factor | Df | R.square | Adj.R.square | F | P |
| --- | --- | --- | --- | --- | --- |
| Sex | 2 | 0.077 | 0.051 | 2.995 | **0.001** |
| Size | 1 | 0.046 | 0.033 | 3.523 | **0.004** |
| Geography | 1 | 0.006 | -0.008 | 0.440 | 0.892 |
| Sex + Size | 3 | 0.104 | 0.066 | 2.739 | **0.001** |
| Sex + Geography | 3 | 0.084 | 0.045 | 2.173 | **0.005** |
| Size + Geography | 2 | 0.052 | 0.026 | 1.970 | **0.017** |
| All | 4 | 0.111 | 0.060 | 2.188 | **0.001** |
| Sex "Pure" | 2 |  | 0.035 | 2.333 | **0.006** |
| Size "Pure" | 1 |  | 0.015 | 2.128 | **0.038** |
| Geography "Pure" | 1 |  | -0.005 | 0.583 | 0.806 |
